# Effect of probiotic supplementation on the gut microbiota diversity in healthy populations: a systematic review and meta-analysis of randomised controlled trials

**DOI:** 10.1186/s12916-025-04602-0

**Published:** 2026-01-07

**Authors:** Anna Júlia Éliás, Kincső Csepke Földvári-Nagy, Yasmin Zubeida Al-Gharati, Dániel Sándor Veres, Tamás Schnabel, Brigitta Teutsch, Bálint Erőss, Péter Hegyi, Katalin Lenti, László Földvári-Nagy

**Affiliations:** 1 Division of Health Sciences, Semmelweis University Doctoral School, Semmelweis University, Budapest, Hungary; 2 Department of Morphology and Physiology, Faculty of Health Sciences, Semmelweis University, Budapest, Hungary; 3 Centre for Translational Medicine, Semmelweis University, Budapest, Hungary; 4 Faculty of Health Sciences, Semmelweis University, Budapest, Hungary; 5 School of Life Sciences, University of Warwick, Coventry, UK; 6 Department of Biophysics and Radiation Biology, Semmelweis University, Budapest, Hungary; 7 Department of Gastroenterology, Skien Hospital, Telemark Hospital Trust, Skien, Norway; 8 Institute of Pancreatic Diseases, Semmelweis University, Budapest, Hungary; 9 Institute for Translational Medicine, Medical School, University of Pécs, Pécs, Hungary

**Keywords:** Probiotics, Meta-analysis, Gastrointestinal microbiome, Diversity, Shannon index, OUT, Chao1 index, Simpson’s index of diversity

## Abstract

**Background:**

Probiotics are widely used dietary supplements promoted to positively influence gut health and microbiota diversity, making them popular among healthy individuals. One of the purported benefits of probiotics is their ability to enhance gut microbiota diversity, a feature associated with improved resilience and overall health. However, evidence supporting this claim remains inconclusive. We aimed to investigate whether probiotics significantly modify gut microbiota diversity in healthy populations through a systematic review and meta-analysis.

**Methods:**

A systematic search of MEDLINE, Embase, and Cochrane databases was conducted on 12/04/2024, following the search strategy registered in PROSPERO (CRD42022286137). Out of 9217 identified articles, 47 met the inclusion criteria of the current review, and 22 studies with data from 1068 individual subjects were eligible for meta-analysis of changes in gut microbiota diversity assessed by diversity indices. A random-effects model was employed to estimate the means of median differences (MedD) with 95% confidence intervals (CI) due to the expected heterogeneity.

**Results:**

The quantitative synthesis revealed no statistically significant effects of probiotics on Shannon diversity (MedD = − 0.08, 95% CI [− 0.16 to 0.01]), observed operational taxonomic units (MedD = 2.19, 95% CI [− 2.20 to 6.57]), Chao1 (MedD = − 3.19, 95% CI [− 27.28 to 20.89]), or Simpson’s index of diversity (MedD = − 0.01, 95% CI [− 0.02 to 0.00]) indices compared to unsupplemented controls. Subgroup and sensitivity analyses suggest that the probiotic taxonomic family, the risk of bias, or the duration of intervention did not change our findings. Insufficient data prevented us from meta-analysing other diversity indices; however, most of the included studies reported no difference in other reported α- and ß-diversity indices between the probiotic and control groups.

**Conclusions:**

Our results indicate that probiotic supplementation does not produce statistically significant changes in gut microbiota diversity in healthy individuals. This study highlights the need for further research to determine whether specific probiotic strains or formulations may influence diversity in targeted subgroups or under specific conditions.

**Supplementary Information:**

The online version contains supplementary material available at 10.1186/s12916-025-04602-0.

## Background

The human gut microbiome, a complex ecosystem of microorganisms, plays a crucial role in various physiological functions, including digestion, immunity, and metabolism [1]. Probiotics, which are live microorganisms that can provide health benefits when used in appropriate amounts, have been extensively studied for their ability to modify the gut microbiota [2, 3]. Despite the uncertainty surrounding probiotics’ effects on the gut microbiome in healthy individuals, these supplements are often recommended to promote overall gut health and well-being. Many consumers believe that probiotics can help restore the balance and diversity of the gut microbiota, especially after disruptions caused by antibiotic use or dietary changes [4]. However, the scientific evidence supporting these claims is lacking.

Diversity refers to the variety of life forms present in a biological system [5]. In the case of the gut microbiome, diversity includes richness (the number of unique taxonomic units) and evenness (the distribution of species to each other) [6–8]. A healthy gut microbiome is characterised by high richness and evenness, with a relatively balanced proportion of various bacterial species [9–11]. Microbial diversity is believed to contribute to functional redundancy within ecosystems, meaning that a more diverse microbiota is better equipped to maintain essential functions even when perturbed. This is one reason why higher diversity is commonly associated with healthier gut states [12, 13]. It is important to note that several methods exist to measure the diversity of the gut microbiome. Standard measures include alpha diversity, which focuses on species richness and evenness within a sample, and beta diversity, which looks at compositional differences between microbial communities [6]. The metric used may affect the interpretation of the study results.

Given the widespread use of probiotics among healthy populations and the variability of existing findings, a systematic evaluation of their impact on diversity indices is warranted. The gap between public expectations and scientific evidence highlights the need for a systematic evaluation of how probiotics affect microbiota diversity, particularly since diversity metrics are commonly used as indicators of gut health in both research and clinical settings. A clearer understanding of whether probiotics can meaningfully alter diversity in healthy populations is essential for informing evidence-based recommendations and for determining whether the perceived benefits of probiotic use align with measurable ecological outcomes.

In the present systematic review and meta-analysis, we aimed to investigate whether probiotics can modify gut microbiota diversity indices in healthy populations.

## Methods

Our study was designed following Cochrane recommendations [14]. The findings are presented following the guidelines of the Preferred Reporting Items for Systematic Reviews and Meta-Analyses (PRISMA) 2020 Statement [15] as detailed in Additional file Table S1. The study protocol was pre-registered with the International Prospective Register of Systematic Reviews (PROSPERO) under the registration number CRD42022286137.

### Search strategy and selection criteria

We formulated our clinical question and defined the eligibility criteria using the PICO-S framework, encompassing Population, Intervention, Comparison, Outcome, and Study Design. The included studies met the following criteria: *Population (P)*—healthy individuals as specified in the articles; *Intervention (I)*—probiotic supplementation delivered as a supplement (e.g., capsule or powder); *Comparison (C)*—no probiotic supplementation (placebo or no intervention); *Outcome (O)*—the primary outcome was gut microbiota diversity (any reported diversity indices) at the end of the intervention (and after a follow-up period). We applied no restrictions on sex, age, or ethnicity. Only randomised controlled trials (RCTs) were included.

A systematic search was conducted across three medical databases—MEDLINE (via PubMed), Embase (via embase.com), and the Cochrane Central Register of Controlled Trials (CENTRAL)—without applying filters or restriction. There were no date, language, or any other restrictions applied. The search combined terms for population characteristics, probiotic type, and study design using Boolean operators: (normal OR general OR healthy) AND (population OR participant OR participants OR volunteer OR volunteers OR subject OR subjects OR adult OR adults OR adolescent OR adolescents OR child OR children OR infant OR infants OR newborn OR newborns OR birth cohort OR pediatric* OR elderly OR elders) AND (probiotic OR probiotic* OR bifidobac* OR lactobac* OR escherichia OR streptococcus OR saccharomyces OR bacillus OR pediococc* OR leuconostoc) AND random*.

Reference lists of included studies and relevant prior systematic reviews were also hand-searched to identify additional eligible records. The search, detailed in Additional file Table S2, was completed on 12/04/2024, aiming to identify all RCTs investigating the effects of probiotics in healthy populations. If published protocols for eligible studies were not identified, additional searches were conducted on the EU Clinical Trials Register [16] and ClinicalTrials.gov [17].

Study selection was facilitated using Rayyan, a web-based tool for systematic reviews [18], alongside EndNote X9 (Clarivate Analytics, Philadelphia, PA, USA) for reference management. Following automated and manual duplicate removal, a stepwise manual selection was performed by two independent researchers (AJÉ, KCSFN, YZA, TS). The initial screening was based on titles and abstracts, followed by full-text assessments against the eligibility criteria. Cohen’s kappa coefficient was calculated at each stage to measure inter-rater agreement, and any discrepancies were resolved through consensus [19].

Eligible studies were analysed by outcome, focusing on the effects of probiotics on gut microbiota diversity, as summarised in this analysis.

### Data collection

Two independent investigators (ÁJÉ, KCSFN) manually extracted data from each article, and their data pools were cross-checked for consistency. Discrepancies were resolved through consensus. The extracted information was recorded in a standardised data collection form. Data included study characteristics (e.g., first author, publication year, country, number of centres, and setting), sample description (e.g., sample size, sex distribution, age, and specific sample characteristics as reported), probiotic details (e.g., type, dose, and duration), and reported outcomes (diversity indices). The different indices are described in detail in Additional file Table S3 [5, 6, 8, 12, 20–42]. For results presented in graphical format, data extraction was performed using GetData Graph Digitizer software (version 2.26.0.20) [43].

### Data analysis

All statistical analyses were performed with *R* software [44] (v4.4.1) using the *meta* [45] (v7.0.0) and *metamedian* [46] (v1.1.1) packages for basic meta-analysis calculations and plots, *metafor* [47] (v4.6.0) and *dmetar* [48] (v0.1.0) package for additional influential analysis calculations and plots. A meta-analysis was performed if the evaluated outcome was reported in at least three articles. As in most cases, the medians and quartiles were given; therefore, the median differences were pooled (mean of median differences estimated) and labelled as MedD (“probiotics” minus control (“no probiotics”)) with 95% confidence intervals (CIs) for the effect size measure. As the main result, we pooled the values of Shannon, Chao1, observed operational taxonomic units (OTUs), and Simpson’s index of diversity indices after treatment, and used the inverse variance weighting method for each separately. We included only RCTs; therefore, we could assume that the characteristics before the treatment were not different in the intervention and control groups. In all cases, a sensitivity analysis was conducted using stricter inclusion criteria, excluding studies with “change” values and pooled cross-over results or unclear data reporting to ensure robustness and consistency in the findings. As we anticipated considerable between-study heterogeneity, a random-effects model was used to pool the effect sizes. We used a Hartung-Knapp adjustment [49, 50] for CIs and for prediction intervals. The maximum-likelihood estimator was applied with the Q profile method for confidence interval to estimate the heterogeneity variance measure *τ*^2^ [51]. Additionally, between-study heterogeneity was described using Cochran’s Q test and Higgins and Thompson’s *I*^2^ statistics [52] too. Forest plots were used to summarise the results graphically. Individual study confidence intervals were presented on the plot using *t*-distribution estimation. We report the results as (MedD [95% CI lower limit to 95% CI upper limit]). Additionally, we synthesised our data, forming subgroups according to the risk of bias, intervention period, and the composition of the probiotics investigated in each study.

Potential outlier publications were explored using different leave-one-out influence measures and plots following the recommendation of Harrer et al. [53]. A short description of these parameters is given in the caption of the figures. We assessed possible small-study effects using Egger’s test, adopting *p* < 0.10 as an indicative threshold in view of its low statistical power; however, this value should not be interpreted as confirmatory evidence of bias, particularly when fewer than ten studies are included [54].

Subgroup analyses were conducted to explore potential variation in probiotic effects by taxonomic family. Studies were categorised into the following subgroups based on the primary probiotic used: *Lactobacillaceae*, *Bifidobacteriaceae*, *Bacillaceae*, *Lactobacillaceae* + *Bifidobacteriaceae* (multistrain formulations combining these taxa), and mixed formulations containing multiple genera. This grouping approach followed our PROSPERO-registered protocol, which specified that subgroup analyses would be performed according to probiotic type when at least three studies per category were available. Grouping at the family level was necessary because only a few studies investigated identical strains, precluding strain-specific meta-analyses. We performed additional analyses (not defined in the PROSPERO) based on the intervention time and the risk of bias. We were not able to perform subgroup analysis based on age and sex (as predefined) as we have lack of data. For subgroup analyses, we used a fixed-effects “plural” model (aka. mixed-effects model). We assumed that all subgroups share a different *τ*^2^ value as we anticipate differences in the between-study heterogeneity, and the study number is not too small in subgroups. If at least one of the subgroups contains less than 6 studies, we assume the same *τ*^2^ at subgroups (recommended in Harrer et al. [53]). In the meta-regression analysis, a linear relationship was assumed, and the weighted least squares method was employed to estimate the regression parameters. The CI and prediction interval (PI) for the slope were calculated based on the *t*-distribution. Additionally, a Wald-type *p*-value was reported for the slope, along with the meta-regression coefficient of determination (*R*^2*^), which serves as a correlation coefficient. For a detailed description of the statistical analysis, see Supplementary Methods S1.

### Risk of bias assessment

Two authors independently assessed the risk of bias using the revised Cochrane risk-of-bias tool (RoB2) [55]. Discrepancies were resolved by consensus. The evaluation covered biases related to the randomisation process, deviations from intended interventions, missing data, outcome measurement, and the selection of reported results. Each domain was rated, and the tool automatically determined the overall risk level, categorised as low, some concerns, or high.

### Certainty assessment

The certainty of the evidence was independently evaluated by two investigators using the Grading of Recommendations, Assessment, Development, and Evaluation (GRADE) framework [56]. Any disagreements were resolved by consensus.

## Results

### Study selection

**Fig. 1:**
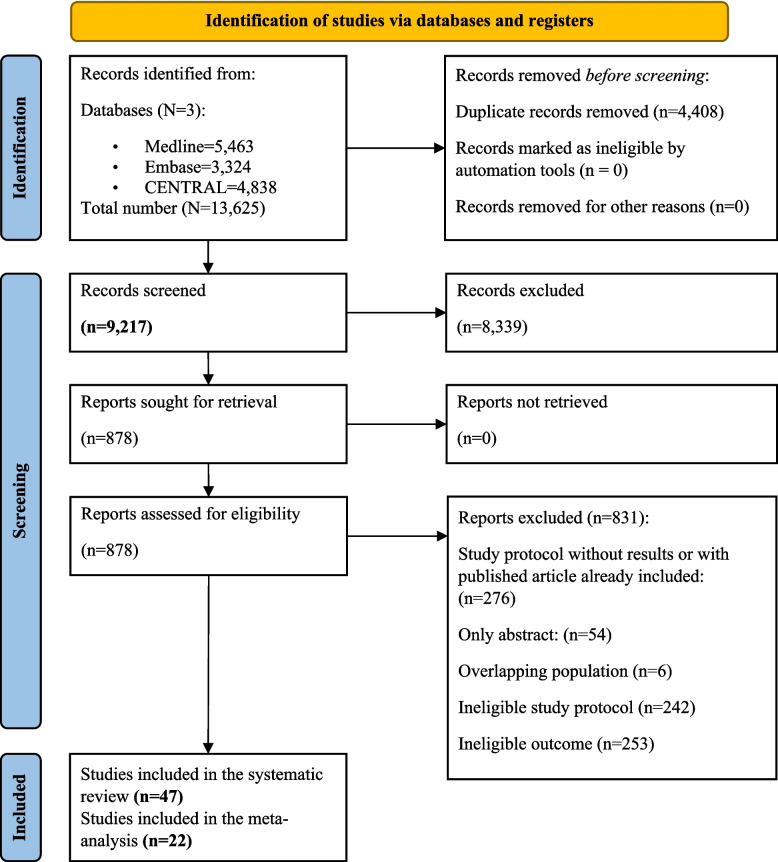
PRISMA flowchart of the selection

The results of the search and selection processes are illustrated in Fig. 1. The search yielded 13,625 records in total, corresponding to 9217 individual records after duplicate removal, which were screened for eligibility. Cohen’s kappa coefficient was calculated to assess inter-rater agreement, achieving values of 0.94 and 0.81 during the title and abstract screening phase and 0.94 and 0.98 during the full-text selection phase. Ultimately, 47 articles met the eligibility criteria for qualitative synthesis of gut microbiota diversity indices.

Study characteristics are summarised in Table 1. The review includes only non-overlapping populations based on the available information to ensure robustness. Most eligible studies focused on adult populations [57, 58, 60, 61, 63, 66–68, 70–72, 74, 75, 78, 80–89, 91–94, 96, 98–103] three investigated elderlies [76, 95, 97]. Six articles specifically examined infants [59, 62, 64, 65, 73, 79], while three studies focused on children [69, 77, 90]. All studies were randomised and placebo-controlled; six used a cross-over design [57, 61, 66, 70, 84, 87].

**Table 1:** The main characteristics of the included studies

Study	Country	Study design (No. of centres*)	Population (randomised)					Probiotic type (as reported in each study**) and dose	Reclassified probiotic nomenclature (if applicable)	Placebo type and dose	Duration (days)	Wash-out period (days) if applicable
			Number of randomised subjects (female %)	Number of subjects in probiotic group	Number of subjects in control group	Age (years - mean ± SD) in the intervention (and control) groups	Specification of the population					
Axelrod (2019) [57]	USA	Randomised, double-blind, placebo-controlled cross-over study	9 (N.D.)	5	4	Whole population: 31 ± 2.3	Healthy, trained endurance athletes	*Lactobacillus salivarius* UCC118 (2 × 10^8^ CFU/day)	*Ligilactobacillus salivarius* UCC118	200 mg corn starch with magnesium stearate	28 days	28 days
Bagga (2018) [58]	Austria	Randomised, double-blind, placebo-controlled clinical trial	30 (47)	15	15	28.27 ± 4.2 (27.25 ± 5.78 and 26.87 ± 4.97)	Healthy volunteers	*Lactobacillus casei* W56*, Lactobacillus* *acidophilus* W22, *Lactobacillus paracasei* W20,* Bifidobacterium* *lactis* W51*, Lactobacillus salivarius* W24*,* *Lactococcus lactis* W19*, Bifidobacterium lactis* W52*,* *Lactobacillus plantarum* W62 and* Bifidobacterium* *bifidum* W23 (7.5 × 10^9^ CFU/g × 3 day)	*Lacticaseibacillus casei* W56 *Lacticaseibacillus paracasei*W20 *Ligilactobacillus salivarius* W24 *Bifidobacterium animalis subsp. lactis* W19 *Lactiplantibacillus plantarum* W62	3 g maize starch and maltodextrins	28 days	NA
Bazanella (2017) [59]	Germany	Double-blind, randomised, and placebo-controlled clinical trial	97 (64)	48	49	Newborns (newborns)	Healthy infants	Control formula plus a total concentration of 10^8^ CFU/g with equal amounts of *Bifidobacterium bifidum* BF3, *Bifidobacterium breve* BR3, *Bifidobacterium longum subspecies* *infantis* BT1, and *Bifidobacterium longum* BG7	NA	Whey-based infant formula	1 year	NA
Bloemendaal (2021) [60]	The Netherlands	Exploratory analysis of a double-blind, randomised, placebo-controlled study	67 (100)	31 (MITT)	33 (MITT)	NA criteria: 18–40	Healthy female subjects	*Bifidobacterium bifidum* W23, *Bifidobacterium lactis* W51, *Bifidobacterium lactis* W52, *Lactobacillus acidophilus* W37, *Lactobacillus brevis* W63, *Lactobacillus casei* W56, *Lactobacillus salivarius* W24, *Lactococcus lactis* W19, and *Lactococcus lactis* W58 (2.5 × 10^9^ CFU/g × 2/day)	*Bifidobacterium animalis subsp. lactis* W51 and W52*Levilactobacillus brevis* W63 *Lacticaseibacillus casei* W56 *Ligilactobacillus salivarius* W24	2 g maize starch, maltodextrin, vegetable protein and a mineral mix	28 days	NA
Boesmans (2018) [61]	Belgium	Randomised, double-blind, placebo-controlled cross-over trial	30 (53)	15	15	32 range: 26–45 (28 range: 25–33)	Healthy volunteers	*Butyricicoccus pullicaecorum* 25-3 T (10^8^ CFU/day)	NA	Maltodextrin	28 days	21 days
Castanet (2020) [62]	France, Greece, Austria	Multicentre, randomised, double-blind, controlled trial (six sites from three countries)	127 (ITT *n* = 202 with not randomised reference group)	44	40	Newborns (newborns)	Healthy full-term vaginal born infants	Control formula + native bovine lactoferrin (1 g/l) and *Bifidobacterium animalis subsp lactis* CNCM I-3446 (3.7 ± 2.1 × 10^4^ CFU/g powder formula)	NA	The control formula was designed to match as closely as possible early maternal milk energy and proteins levels	28 days	NA
Chen (2021) [63]	China	Double-blinded randomised controlled trial	40 (100)	20	20	22.7 ± 1.5 (23.0 ± 1.4)	Healthy males	*Lactobacillus rhamnosus* GG*, Lactobacillus* *acidophilus, Bifidobacterium animalis* and* Bifidobacterium longum* (1.32 × 10^11^ CFU/day)	*Lacticaseibacillus rhamnosus* GG	Starch, maltodextrin and sugar	28 days	NA
Chen (2023) [64]	China	Three-arm, randomised, double-blind, placebo-controlled study	88 (mITT 86 → 57)	29	31	1.2 (0.5) (month) 1.3 (0.5)	healthy infants	*Lactobacillus salivarius* AP-32 (2.5 × 10^9^ CFU × 2/day)	*Ligilactobacillus salivarius* AP-32	0.5 g maltodextrin	4 month	NA
				28		1.3 (0.4) (months) 1.3 (0.5)		*Bifidobacterium animalis subspecies lactis* CP-9 (2.5 × 10^9^ CFU × 2/day)	NA			
De Andrés (2018) [65]	Spain	Secondary analysis of a randomised, double-blind, placebo controlled, multicentre intervention study (NI)	219 (ITT) 202 (mITT) 198 (PP)—subgroup of 92 infants	23	23	NA—only for the original population	Infants from 3 to 12 months of age, breastfed and/or formula fed	*Bifidobacterium infantis* (3 × 10^9^ CFU/day)	*Bifidobacterium longum subsp. infantis*	Potato starch	56 days	NA
	Spain			23				*Lactobacillus helveticus* R0052 (3 × 10^9^ CFU/day)	NA			
	Spain			23				*Bifidobacterium bifidum* R0071 (3 × 10^9^ CFU/day)	NA			
Ferrario (2014) [66]	Italy	Randomised, double-blind, cross-over placebo-controlled study	30 (60)	30	30	35 ± 10,7	Healthy volunteers	*Lactobacillus paracasei* DG (24 × 10^9^ CFU/day)	*Lacticaseibacillus paracasei DG*	Placebo (NI)	28 days	28 days
Freedman (2021) [67]	USA	Parallel arm, double-blind, randomised, placebo-controlled intervention study	46 (61)	25	21	36.9 ± 12.9 (34.4 ± 13.0)	Normal weight to mildly obese healthy adults	*Bacillus subtilis* strain DE111 (1 × 10^9^ CFU/day)	NA	Maltodextrin	28 days	NA
Gai (2023) [68]	China	Single-blind placebo-controlled trial	100 (analysed 94 → 66)	50	50	Only available for the analysed population 22.6 ± 1.6 (23.0 ± 2.4)	Healthy volunteers	*Lacticaseibacillus rhamnosus* strain LRa05 (1 × 10^10^ CFU/day)	NA	2.0 g maltodextrin	28 days	NA
Gan (2022) [69]	China	Randomised, single-blind, placebo-controlled, multicentre clinical trial (2)	100 (analysed 92 (50)	50	50	Only available for the analysed population 8.4 (8.1)	Children with functional constipation according to Rome III criteria	*Lactobacillus acidophilus* DDS-1 R and *Bifidobacterium animalis subsp. lactis* UABla-12TM (5 × 10^9^ CFU × 2/day)	NA	Hydroxymethyl cellulose magnesium stearate,	28 days	NA
Gargari (2016) [70]	Italy	Randomised, double-blind, cross-over, and placebo-controlled intervention study	35	35	35	NI for the randomised population	Healthy volunteers	*Bifidobacterium bifidum* Bb (3.8 × 10^9^ CFU/day)	NA	Maltodextrin, cellulose powder, dextrose, a separating agent (magnesium salts of edible fatty acid), and silica	28	28
Hanifi (2015) [71]	USA	Double-blinded, placebo-controlled, randomised trial	83 → 41 (59)	21	20	Median and range 23 (20–49) (23 (20–46))	Healthy volunteers	*Bacillus subtilis* R0179 (0.1 × 10^9^ CFU/day)	NA	Placebo (NI)	28 days	NA
			83 → 40 (48)	20		Median and range 22 (20–31) (23 (20–46))		*Bacillus subtilis* R0179 (1 × 10^9^ CFU/day)	NA			
			83 → 42 (NI)	22		Median and range NI (23 (20–46))		*Bacillus subtilis* R0179 (10 × 10^9^ CFU/day)	NA			
Hibberd (2019) [72]	Finland	PP subset of a double-blind, randomised, parallel, placebo-controlled clinical trial (4)	225 → PP 134 → 61 (72)	25	36	49.1 ± 11.9 (48.3 ± 8.6)	Overweight or obese (body mass index (BMI) 28.0–34.9) but otherwise healthy volunteers	*Bifidobacterium animalis subsp.lactis* 420™ (B420) (10^10^ CFU/day)	NA	12 g/day of microcrystalline cellulose	6 months	NA
Hiraku (2023) [73]	Japan	Placebo-controlled, double-blinded, randomised trial	111 (only available for compliant participants: 52)	57	54	Newborns (newborns)	Healthy full-term infants	*Bifidobacterium infantis* M-63 (1 × 10^9^ CFU/1.0 g of sachet)	*Bifidobacterium longum subsp. infantis* M-63	Sterilised dextrin only/1.0 g of sachet	3 months	NA
Huang (2022) [74]	China	Randomised-controlled trial	31 (100)	15	16	Analysed population 27.42 ± 3.09 (27.33 ± 2.90)	Pregnant women before 32 weeks of gestation	*Bifidobacterium longum* (0.5 × 10^7^ CFU × 4), *Lactobacillus delbrueckii supsp. bulgaricus* (0.5 × 10^6^ CFU × 4), and *Streptococcus thermophilus* (0.5 × 10^6^ CFU × 4)/day	NA	Nothing	52 ± 7.08 days	NA
Kang (2021) [75]	Korea	Randomised, double-blind, placebo-controlled, parallel-group trial	80 (88)	40	40	(Mean ± SE) 44.4 ± 2.2 (45.3 ± 1.8)	Modified Rome III functional constipation criteria fulfilling adults, otherwise healthy	Spore-forming *Bacillus coagulan*s SNZ 1969 (1.0 × 10^9^ CFU/day)	*Heyndrickxia coagulans* SNZ 1969	Maltodextrin	56 days	NA
Kim (2021) [76]	Korea	Randomised, double-blind, placebo-controlled, multicentre clinical trial (2)	63	32	31	NI for the randomised population only for analysed population *n* = 27 71.11 ± 5.02 (n = 26 72.00 ± 3.36)	Community- dwelling older adults (65 +)	*Bifidobacterium* *bifidum* BGN4 and *Bifidobacterium longum* BORI in soybean oil (1 × 10^9^ CFU/day)	NA	500 mg of soybean oil	84 days	NA
Lau (2018) [77]	Malaysia	Randomised, double-blind, parallel and placebo-controlled study	520 (52)	259	261	4.2 ± 1.3 (4.1 ± 1.3)	Healthy pre-school children aged 2–6 years	*Bifidobacterium* *longum* BB536 (5 × 10^9^ CFU)	NA	Maltodextrin (1 g)	10 months	NA
Lee (2021) [78]	Korea	Randomised, double-blind, placebo-controlled trial PP analysis	156 (NI)	78	78	NI for the randomised population, only for the analysed population *n* = 63 38.86 ± 10.89 (*n* = 59 37.63 ± 11.04)	Healthy adults aged 19 to 65 years with psychological stress and subclinical symptoms of depression or anxiety	*Lactobacillus reuteri* NK33 (2 × 10^9^ CFU × 2/day) and *Bifidobacterium adolescentis* NK98 (0.5 × 10^9^ CFU × 2/day)	*Limosilactobacillus reuteri* NK33	500 mg maltodextrin	56 days	NA
Li (2023) [79]	China	Single-centre, randomised, triple-blind placebo-controlled trial	109 (101 finished → 52%)	51	50	NI (6–24 months)	Healthy infants delivered by C-section	*Lactobacillus paracasei* N1115 (2 × 10^10^ CFU/g)	*Lacticaseibacillus paracasei* N1115	Maltodextrin	3 months	NA
López-Garcia (2023) [80]	Spain	Randomised, placebo-controlled, single-blind study	39 (51)	20	19	31.45 ± 8.28 (33.63 ± 6.96)	Healthy volunteers	*Lactiplantibacillus* *pentosus* LPG1 (1 × 10^10^ CFU/day)	*Lactiplantibacillus pentosus* LPG1	Dextrose	30 days	NA
Majeed (2023) [81]	India	Randomised, double-blind, placebo-controlled trial	30 (63)	15	15	37.67 ± 10.65 (39.50 ± 9.15)	Healthy adults	*Bacillus coagulans (Weizmannia coagulans*) microbial type culture collection 5856 (LactoSpore®) (2 × 10^9^ CFU/day)	*Heyndrickxia coagulans*	Maltodextrin	28 days	NA
Marcial (2017) [82]	USA	Randomised double-blind placebo-controlled parallel study	42 (72)	21	21	Mean and range 23 (18–36) ((21 (18–48))	Healthy adults	*Lactobacillus johnsonii* N6.2 (10^5^ CFU/day)	NA	NI	56 days	
Michael (2020) [83]	United Kingdom	Exploratory, block-randomised, parallel, double-blind, single-centre, placebo-controlled superiority study	220 (60)	110	110	45.30 ± 10.20 46.52 ± 9.93	Volunteers with a waist circumference > 89 cm (women) or > 100 cm (men); a body mass index (BMI, kg/m^2^) between 25 and 34.92	*Lactobacillus acidophilus* CUL60 (NCIMB 30,157*), Lactobacillus acidophilus* CUL21 (NCIMB 30156), *Lactobacillus plantarum* CUL66 (NCIMB 30280) *Bifidobacterium bifidum* CUL20 (NCIMB 30153) and *Bifidobacterium animalis subsp. lactis* CUL34 (NCIMB 30,172) on a base of microcrystalline cellulose (5 × 10^10^ CFU/day)	*Lactiplantibacillus plantarum* CUL66 (NCIMB 30280)	Microcrystalline cellulose	180 days	NA
Moloney (2021) [84]	Ireland	Double-blind, randomised, placebo-controlled, repeated measures, cross-over design	30 (0)	15 (first)	15 (second)	20.7 (SEM 0.28)	Male university students	*Bifidobacterium longum* AH1714 (1 × 10^9^ CFU/day)	NA	Corn starch, magnesium stearate, hypromellose, titanium dioxide	56 days	Unknown
Moore (2023) [85]	Ireland	Single-centre, double-blind, placebo-controlled, randomised controlled trial	160 (100)	80	80	all 33.6 ± (3.9)	Pregnant women	*Bifidobacterium breve* 702,258 (minimum 1 × 10^9^ CFU/day)	NA	Standard excipients	From 16 weeks gestation until 3 months postpartum	NA
Mutoh (2024) [86]	Japan	Randomised, double-blind, placebo-controlled, parallel-group clinical trial	30 (47)	15	15	46.3 ± 11.6 (47.9 ± 11.6)	Volunteers	*Bifidobacterium breve* M-16 V (2 × 10^9^ CFU/day)	NA	Maltodextrin	42	NA
Nakamura (2022) [87]	Japan	Randomised, Double-blind, controlled cross-over trial	24 (60)	12	12	47.7 ± 5.8 (all participants)	Healthy volunteers with constipation	*Bifidobacterium longum* BB536 (5.0 × 10^9^ CFU/day)	NA	Potato starch	14 days	28 days
Pagliai (2023) [88]	Italy	Randomised, double-blinded parallel controlled trial	40 (50)	20	20	51 ± 12.7 (51 ± 14.7)	Overweight or obese (body mass index (BMI) 28.0–34.9) but otherwise healthy population (BMI ≥ 25 kg/m^2^)	*Lactiplantibacillus plantarum* IMC 510® (1.5 × 10^10^ CFU/capsule)	NA	Placebo (NI)	3 months	NA
Park (2020) [89]	Korea	Double-blind, randomised, placebo-controlled	70 (66)	35	35	48.3 ± 11.6 (8.3 ± 13.2)	Healthy volunteers	*Lactobacillus plantarum* LPQ180 (2 × 400 mg/2 × 4 × 10^9^ CFU/day)	*Lactiplantibacillus plantarum* LPQ180	2 × 400 mg maltodextrin	84 days	NA
Paytuvi-Gallart (2020) [90]	Spain	Randomised, parallel, double-blind, placebo-controlled study	102 (only available for the at-the-end investigated population)	51	51	Only available for the at-the-end investigated population 2–6 years	Healthy children attending day-care	*Bacillus subtilis* DE111 (1 × 10^9^ CFU/dose)	NA	Dextrose, tapioca maltodextrin, natural flavour and l-leucine	56 days	NA
Plaza-Diaz (2015) [91]	Spain	Randomised, placebo-controlled trial	25 (36)	4	5	25.5 ± 6.9 (26.6 ± 3.9)	healthy volunteers	*Bifidobacterium breve* CNCM I-4035 (9 × 10^9^ CFU/day)	NA	NI	30 days	NA
				5		23.6 ± 4.5 (26.6 ± 3.9)		*Lactobacillus rhamnosus* CNCM I-4036 (9 × 10^9^ CFU/day)	*Lacticaseibacillus rhamnosus* CNCM I-4036			
				4		25.5 ± 4.2 (26.6 ± 3.9)		*Bifidobacterium breve* CNCM I-4035 and *Lactobacillus rhamnosus* CNCM I-4036 (9 × 10^9^ CFU/day)	*Lacticaseibacillus rhamnosus* CNCM I-4036			
				5		27,2 ± 2,1 (26.6 ± 3.9)		*Lactobacillus paracasei* CNCM I-4034 (9 × 10^9^ CFU/day)	*Lacticaseibacillus paracasei* CNCM I-4034			
Qian (2020) [92]	China	Controlled trial	Total of 36, in the groups of our interest *n* = 18 (50)	9	9	M ± SEM 55.8 ± 8.8 (51.7 ± 5.0)	Healthy volunteers typically consuming high-fat diet	*Bifidobacterium longum* (≥ 1.0 × 10^7^ CFU/g), *Lactobacillus acidophilus* (≥ 1.0 × 10^7^ CFU/g) and *Enterococcus faecalis* (≥ 1.0 × 10^7^ CFU/g)	NA	No intervention	4 months	NA
Rahayu (2021) [93]	Indonesia	Randomised, double-blind, placebo controlled study	60 (60)	30	30	44.07 ± 6.23 (44.67 ± 5.66)	Healthy overweight adults (body mass index (BMI) equal to or greater than 25)	*Lactobacillus* *plantarum* Dad-13 (2 × 10^9^ CFU/gram/sachet)	*Lactiplantibacillus plantarum* Dad-13	1 g skimmed milk powder	90 days	NA
Sánchez Macarro (2021) [94]	Spain	Randomised double-blind and controlled single-centre clinical trial	44 (0)	22	22	25.3 ± 7.2 years ((27.1 ± 8.4 years)	Caucasian, healthy male volunteers who performed aerobic physical exercise between 2 and 4 times a week	*Bifidobacterium longum* CECT 7347, *Lactobacillus casei* CECT 9104, and *Lactobacillus rhamnosus* CECT 8361 (in a ratio 1:4.5:4.5, 1 × 10^9^ total CFU/day)	*Lacticaseibacillus casei* CECT 9104 *Lacticaseibacillus rhamnosus* CECT 8361	300 mg capsules with maltodextrin and sucrose	42 days	NA
Sandionigi (2022) [95]	Italy	Placebo-controlled, randomised, double-blind, clinical trial	50 (72)	25	25	Probiotics: 63:71 ± 5:28 (female) 60:00 ± 3:32 (male)//placebo 60:00 ± 3:32 (female) 60:00 ± 3:32 (male)	Flu-vaccinated healthy elderly subjects	*Lactiplantibacillus plantarum subsp. plantarum* (formerly *Lactobacillus plantarum*) PBS067 (1 × 10^9^ CFU), *Bifidobacterium animalis subsp. lactis* BL050 (1 × 10^9^ CFU) *Bifidobacterium longum subsp. infantis* BI221 (1 × 10^9^ CFU), *Bifidobacterium longum subsp. longum* BLG240 (1 × 10^9^ CFU/day)	NA	Placebo (NI)	28 days	NA
Shi (2020) [96]	China	Prospectively randomised controlled	50 (70)	25	25	40:6 ± 11:0 (43:2 ± 12:2)	Adults with the gastrointestinal symptoms of abdominal pain, abdominal bloating, abdominal distension, or bowel habit abnormalities (constipation, diarrhoea, or mixed constipation and diarrhoea)	Medilac-S (live combined *Bacillus subtilis* and *Enterococcus* *faecium* (500 mg per time, × 3/day)	NA	No intervention	28 days	NA
Shi (2023) [97]	China	A randomised, double-blind, placebo-controlled trial	60 (58)	30	30	64.10 3.40 (64.50 ± 3.79)	Healthy elderly people aged 60–75 years,	*Bifidobacterium longum* BB68S (BB68S, CGMCC No. 14168) (5 × 10^10^ CFU/sachet)	NA	Maltodextrin	56 days	NA
Simon (2015) [98]	Germany	Double-blind, 1:1 randomised, prospective, longitudinal pilot trial	21 (52)	11	10	50 ± 6.7 (all population)	Glucose-tolerant volunteers,	*Limosilactobacillus reuteri* SD5865 (2 × 10^10^ CFU/day)	NA	NI	28 days	NA
Sohn (2022) [99]	South Korea	Randomised, double-blind controlled clinical trial	81 (60)	41	40	47.8 ± 11.7 (45.5 ± 10.0)	Healthy men and women aged 20 to 65 years with a BMI of 25–30 kg/m^2^	*Lactobacillus plantarum* K50 (4 × 10^9^ CFU/day)	*Lactiplantibacillus plantarum* K50	Microcrystalline cellulose powder,	84 days	NA
Son (2020) [100]	Korea	Randomised-controlled trial	20 (0)	10	10	Without dropouts 26.50 ± 5.01 (27.14 ± 5.93)	Bodybuilders who consumed an extremely high-protein/low-carbohydrate diet	*Lactobacillus acidophilus*, *Lacticaseibacillus casei*, *Lactobacillus helveticus*, and *Bifidobacterium bifidum* (10^12^ CFU of each/day)	NA	Corn starch	60 days	NA
Tremblay (2021) [101]	United States of America	Double-blind, randomised, parallel design study	69 (68 ended – 63%)	23	23	Median and range 22 (18–30) (27 (18–31)	Volunteers	*Lactobacillus helveticus* R0052, *Lactobacillus rhamnosus* R0011, *Lactobacillus casei* R0215, *Pediococcus acidilactici* R1001, *Bifidobacterium breve* R0070, *Bifidobacterium longum subsp. longum* BB536 *Lactobacillus* *plantarum* R1012, *Lactococcus lactis subsp. lactis* R1058 (5 × 10^9^ CFU/day)	*Lacticaseibacillus rhamnosus* R0011 *Lacticaseibacillus casei* R0215 *Lactiplantibacillus plantarum* R1012	Potato starch, magnesium stearate, and vitamin C	28 days	NA
Washburn (2022) [102]	United States of America	Randomised placebo-controlled trial	32 → 30 (50)	16	16	Analysed population 29 (25)	Self-reported healthy adults	*Bifidobacterium infantis* (1 × 10^9^ CFU/day)	*Bifidobacterium longum subsp. infantis*	Empty gelatine capsules	30 days	NA
Wischmeyer (2024) [103]	United States of America	A randomised, double-blind, placebo-controlled trial	182 (63)	91	91	NI	Exposed household contacts (individuals living with someone recently diagnosed with COVID-19)	*Lacticaseibacillus rhamnosus GG (ATCC 53103)* 10^10^ CFU/capsule (age < five, one capsule daily, age > five, two capsules daily)	NA	325 mg of microcrystalline cellulose	28 days	NA

Abbreviations: *NI* no information, *NA* not applicable, *CFU* Colony Forming Unit, *ITT* intention-to-treat, *mITT* modified intention-totreat, *PP* per-protocol, *subsp* subspecies

*If not otherwise mentioned, the studies were single centres

**There has been a major reclassification of bacterial genera, resulting in some articles using the old nomenclature while others adopt the updated names

Although we identified forty-seven eligible articles reporting the results of gut microbiota diversity, only twenty-two for the Shannon diversity index [60, 63, 67, 68, 72, 74, 75, 80, 81, 83–85, 87, 89, 94–97, 99–102], seven for the OTUs index [60, 78, 84, 85, 93, 94, 96], nine for the Chao1 index [68, 70, 75, 81, 83, 84, 93, 97, 102], and ten for the Simpson’s index of diversity [68, 74, 80, 81, 84, 85, 94, 96, 97, 100] provided data, either in acceptable numerical format or via boxplots, for including into the meta-analysis. We handled articles reporting data on the infant or children population separately to prevent the indirectness of our findings. We excluded them from the meta-analysis and included them only in the systematic review to preserve the reliability of our results. Age-related findings and their potential influence on probiotic responsiveness are further discussed in the “Age-group differences” section. Definitions of gut microbiota diversity in the included studies are summarised in Additional file Table S3.

### Study characteristics

The main characteristics of the included studies can be found in Table 1.

### The impact of probiotic supplementation on Shannon diversity index in healthy populations

**Fig. 2:**
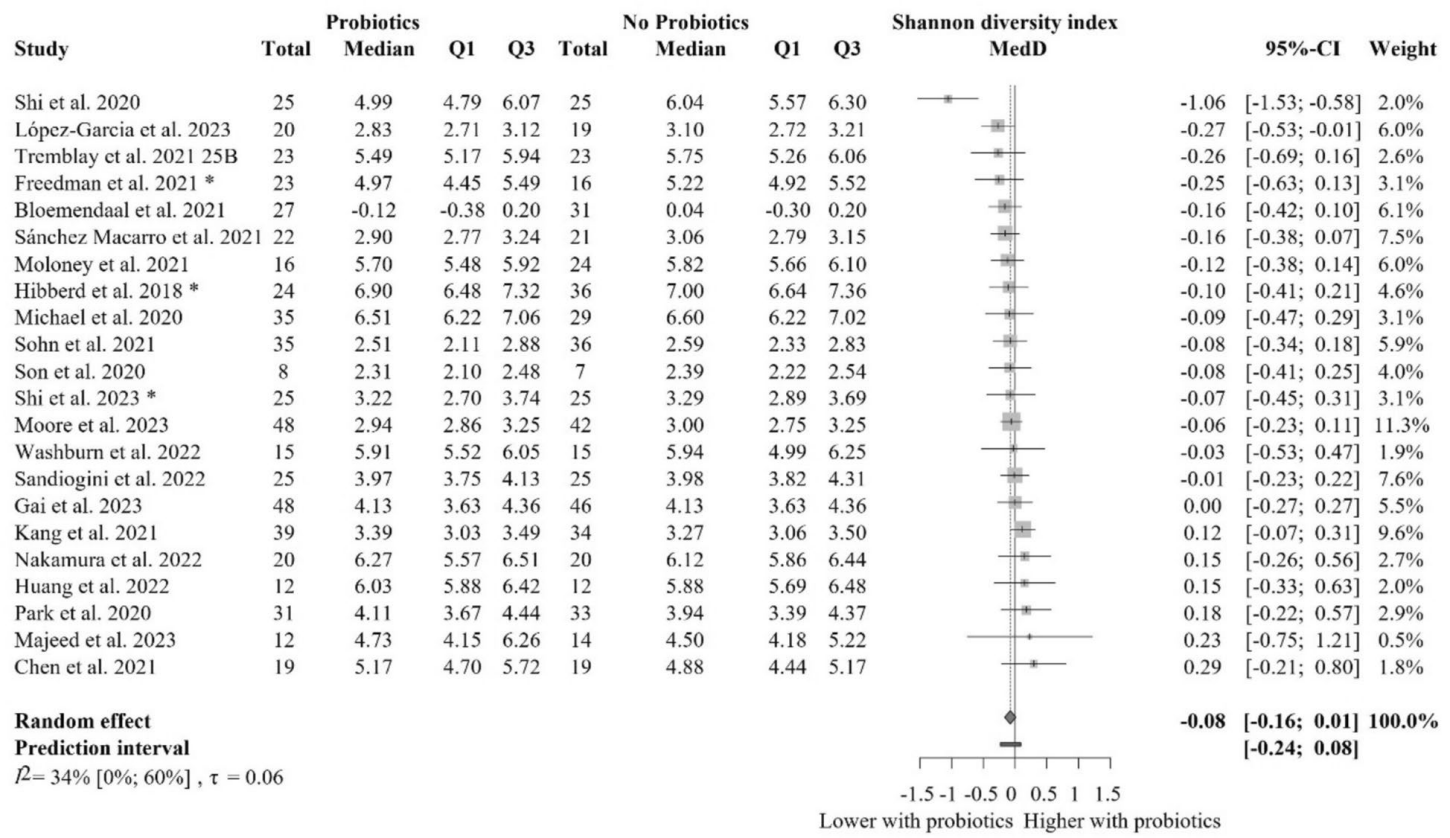
Forest plot of the Shannon diversity index: no significant difference between probiotic and control groups after treatment. Abbreviations: CI, confidence interval; MedD, mean of median differences; Q1, first quartile; Q3, third quartile. The “*” indicates that the median and q1 and q3 were estimated from the mean and standard deviation in that study

The meta-analysis of the Shannon diversity index, including twenty-two articles with 1068 individual participants [60, 63, 67, 68, 72, 74, 75, 80, 81, 83–85, 87, 89, 94–97, 99–102], is shown in Fig. 2. Shannon diversity index was not significantly or relevantly different between the intervention and control groups at the end of treatment (MedD = − 0.08 [− 0.16 to 0.01]).

**Fig. 3:**
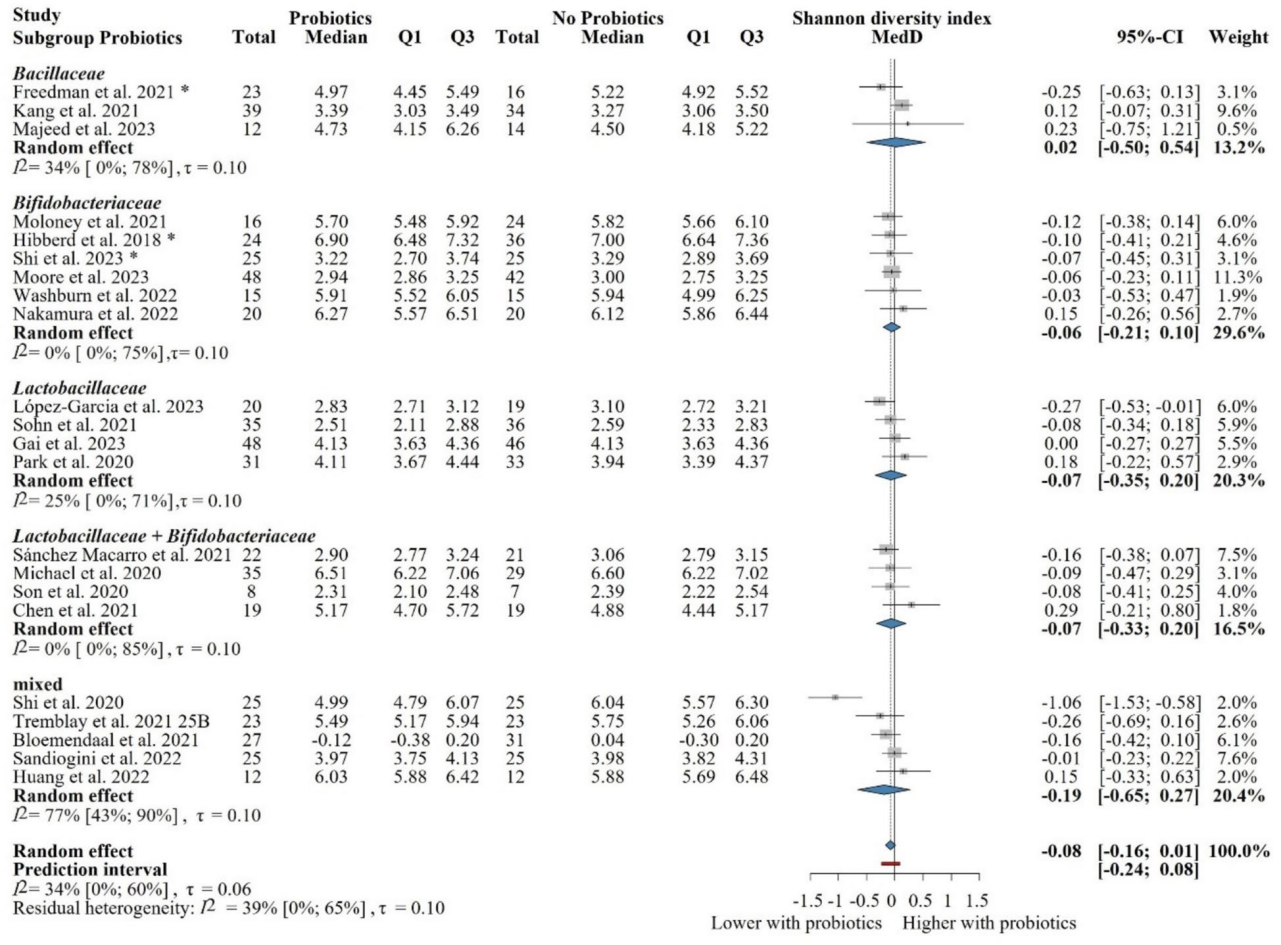
Subgroup analysis of the Shannon diversity index: no significant difference between probiotic and control groups after treatment across probiotic strain families. Abbreviations: CI, confidence interval; MedD, mean of median differences; Q1, first quartile; Q3, third quartile. The “*” indicates that the median and q1 and q3 were estimated from the mean and standard deviation in that study

We performed subgroup analysis based on the probiotic composition used in each study. Three major groups of articles were identified that investigated the effect of bacteria belonging to the family of *Lactobacillaceae*, *Bifidobacteriaceae*, and *Bacillaceae* or, additionally, a mixture of these. Neither of these groups showed any significant effect, as shown in Fig. 3.

As a sensitivity analysis, we performed a separate calculation with more restricted inclusion criteria without studies with cross-over design [84, 87] providing change data only [60] or with no clear number of participants [85]. We did not find any significant difference between the groups in this case either (Additional file Fig. S1). When analysing our data based on the risk of bias assessment (high, some concerns, or low risk of bias), we did not reveal any significant or clinically relevant difference between the intervention and control groups (Additional file Fig. S2). The meta-regression of intervention duration showed no association with effect size (slope = 0.00, 95% CI [− 0.01 to 0.02], *p* = 0.60; *R*^2^* = 0%) (Additional file Fig. S3).

### The impact of probiotic supplementation on observed OTU diversity index in healthy populations

**Fig. 4:**
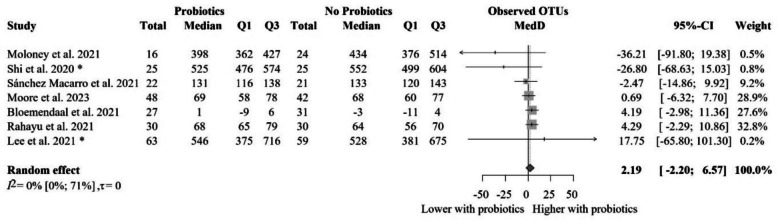
Forest plot of the observed OTUs: no significant difference between probiotic and control groups after treatment. Abbreviations: OTU, operational taxonomic unit; CI, confidence interval; MedD, mean of median differences; Q1, first quartile; Q3, third quartile. The “*” indicates that the median and q1 and q3 were estimated from the mean and standard deviation in that study

**Fig. 5:**
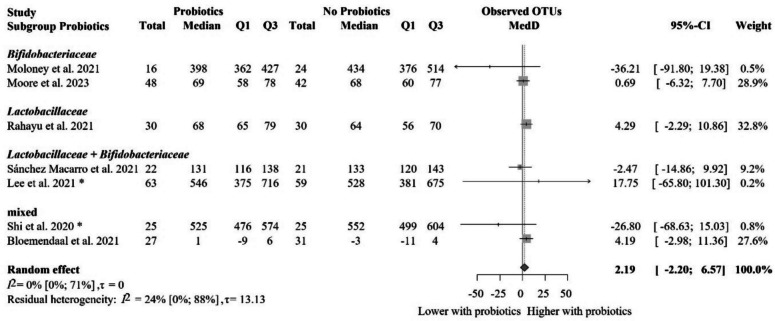
Subgroup analysis of observed OTUs: no significant difference between probiotic and control groups after treatment across probiotic strain families. Abbreviations: OTU, operational taxonomic unit; CI, confidence interval; MedD, mean of median differences; Q1, first quartile; Q3, third quartile. The “*” indicates that the median and q1 and q3 were estimated from the mean and standard deviation in that study. Note: Prediction intervals are omitted when *τ* = 0, as they coincide with the pooled confidence interval under conditions of no between-study heterogeneity

We identified seven eligible articles with 447 individual participants in total for the meta-analysis of the number of observed OTUs [60, 78, 84, 85, 93, 94, 96] (Fig. 4). The mean of median differences in the number of observed OTUs between the two groups was 2.19 (− 2.20 to 6.57), meaning that the two groups did not meaningfully differ from each other. We did not identify significant differences when removing studies with no clear data on the number of participants [85], cross-over design [84], and change results [60] (Additional file Fig. S4). Subgroups based on the probiotic composition resulted in no significant and clinically irrelevant differences (Fig. 5). Similarly, subgroup analysis based on the risk of bias did not lead to significant findings (Additional file Fig. S5). The meta-regression of intervention duration showed no association with effect size, assuming a linear relationship (slope = − 0.06, 95% CI [− 0.77 to 0.65], *p* = 0.84; *R*^2^* = 0%). These results indicate that the length of probiotic intervention did not influence gut microbiota diversity (Additional file Fig. S6).

### The impact of probiotic supplementation on the Chao1 index in healthy populations

**Fig. 6:**
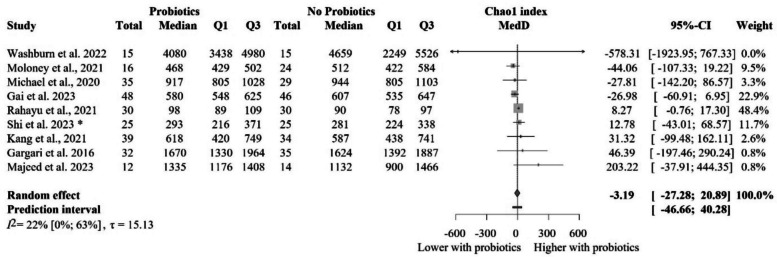
Forest plot of the Chao1 index: no significant difference between probiotic and control groups after treatment. Abbreviations: CI, confidence interval; MedD, mean of median differences; Q1, first quartile; Q3, third quartile. The “*” indicates that the median and q1 and q3 were estimated from the mean and standard deviation in that study

**Fig. 7:**
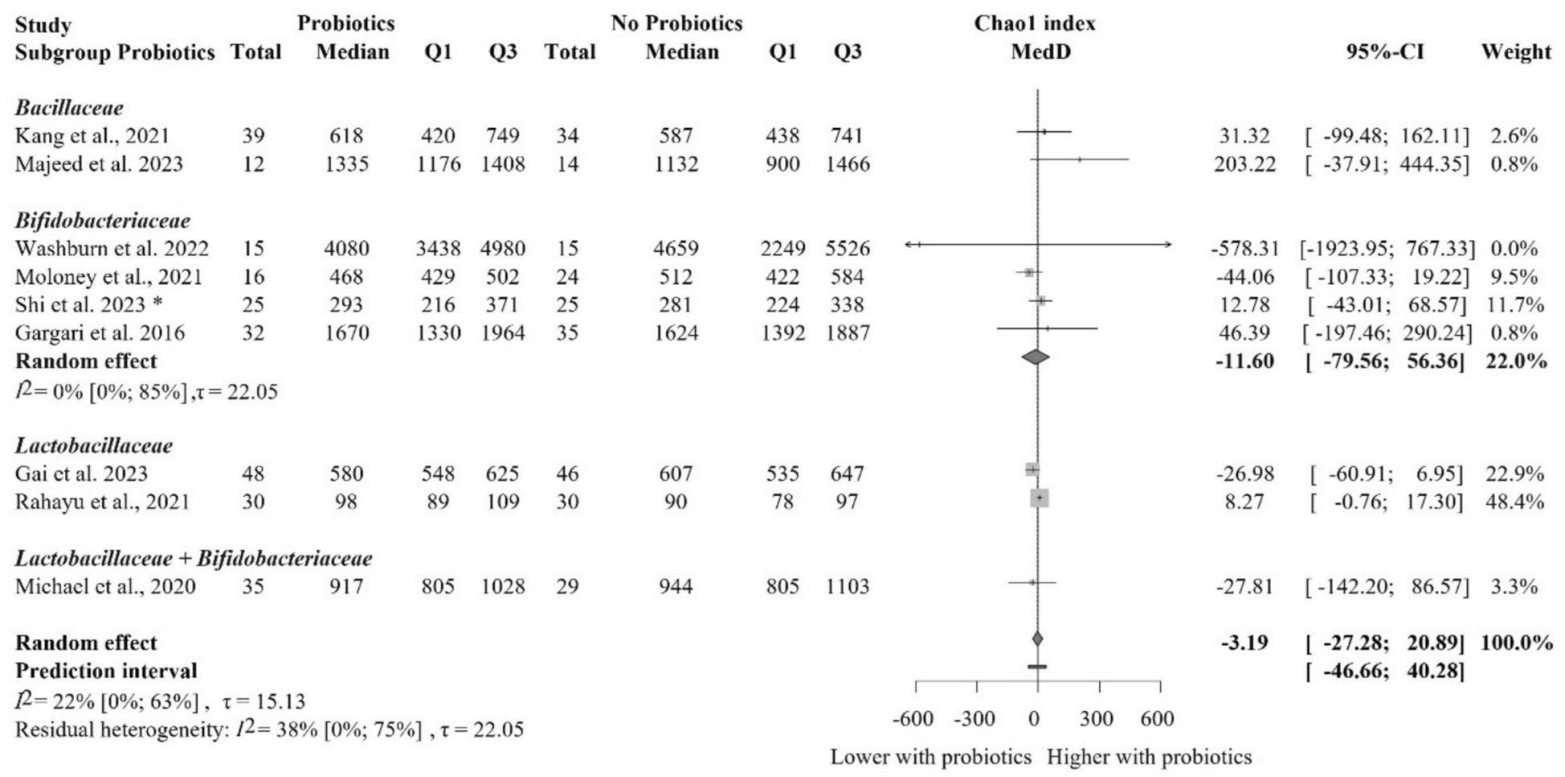
Subgroup analysis of the Chao1 index: no significant difference between probiotic and control groups after treatment across probiotic strain families. Abbreviations: CI, confidence interval; MedD, mean of median differences; Q1, first quartile; Q3, third quartile. The “*” indicates that the median and q1 and q3 were estimated from the mean and standard deviation in that study

Nine eligible articles with 456 individual participants provided the data on the Chao1 index for quantitative analysis [68, 70, 75, 81, 83, 84, 93, 97, 102] (Fig. 6). Probiotic supplementation did not result in a significantly different Chao1 index compared to the control group MedD = − 3.19 [− 27.28 to 20.89], even when we performed the subgroup analysis based on the probiotic composition (Fig. 7). These results are not clinically relevant either.

The more restricted sensitivity analysis removing studies with cross-over design [70, 84] revealed no significant or relevant difference between groups either (Additional file Fig. S7). Subgroup analyses based on the risk of bias assessment is shown in Additional file Fig. S8, with no significant differences between the groups. The meta-regression of intervention duration showed no significant association with effect size, assuming a linear relationship (slope = 1.75, 95% CI [− 4.01 to 7.51], *p* = 0.50; *R*^2^* = 41.3%). These findings suggest that probiotic intervention duration did not meaningfully affect the observed outcomes (Additional file Fig. S9).

### The impact of probiotic supplementation on the Simpson’s index of diversity in healthy populations

We performed the meta-analysis using the Simpson’s index of diversity, standardising all data to reflect higher values indicating greater diversity. However, it was not always explicitly stated in each article whether Simpson’s index of diversity (1-D) or Simpson’s index (D) was used. Based on the reported values (typically ranging from 0.8 to 0.9), it is likely that the Simpson’s index of diversity was applied rather than the traditional Simpson’s index, which measures the probability of two random samples belonging to the same species. This approach allowed consistency in interpreting higher values as greater diversity across studies.

**Fig. 8:**
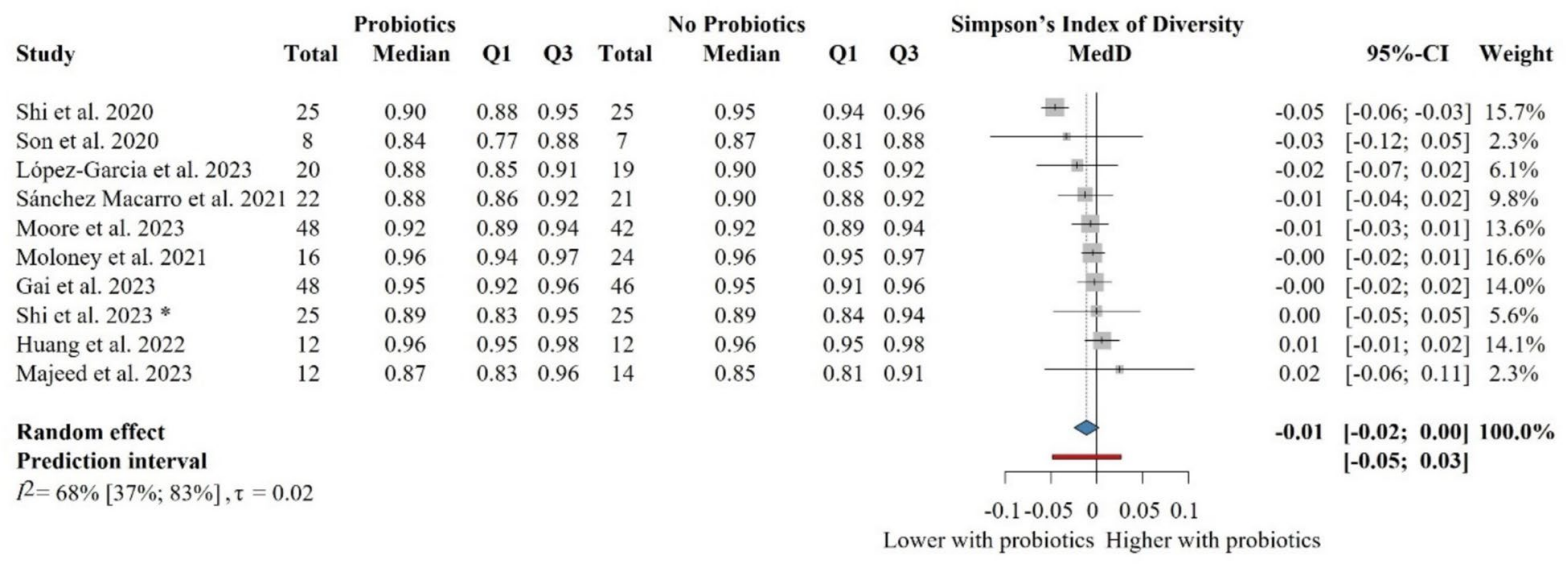
Forest plot of the Simpson’s index of diversity: no significant difference between probiotic and control groups after treatment. Abbreviations: CI, confidence interval; MedD, mean of median differences; Q1, first quartile; Q3, third quartile. The “*” indicates that the median and q1 and q3 were estimated from the mean and standard deviation in that study

**Fig. 9:**
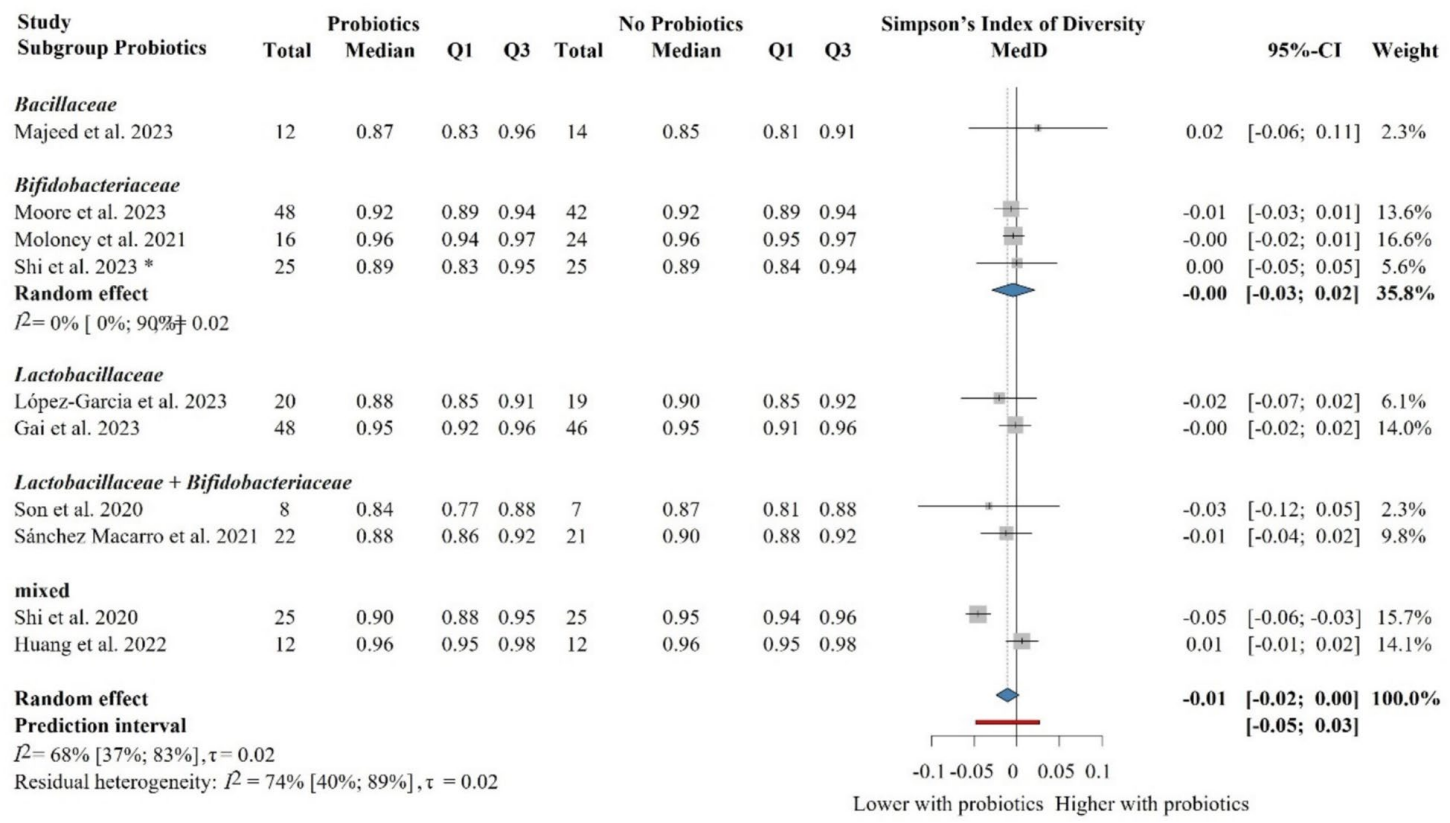
Subgroup analysis of the Simpson’s index of diversity: no significant difference between probiotic and control groups after treatment across probiotic strain families. Abbreviations: CI, confidence interval; MedD, mean of median differences; Q1, first quartile; Q3, third quartile. The “*” indicates that the median and q1 and q3 were estimated from the mean and standard deviation in that study

Ten eligible articles with 455 individual participants were included in the meta-analysis on the Simpson’s index of diversity [68, 74, 80, 81, 84, 85, 94, 96, 97, 100] (Fig. 8). The probiotic-supplemented group was not significantly or relevantly different from the control group: MedD = − 0.01 (− 0.02 to 0.00). Similarly, the subgroup analysis based on the composition of probiotic supplementation did not bring significant results in all cases (Fig. 9).

In the sensitivity analysis, we removed the study with a cross-over design [84] and the one with no precise number of participants [85], but we did not reveal any effect of probiotics either (Additional file Fig. S10). The subgroup analyses based on the risk of bias did not bring significant results either (Additional file Fig. S11). The meta-regression of intervention duration showed no association with effect size, assuming a linear relationship (slope = 0.00, 95% CI [0.00 to 0.00], *p* = 0.51; *R*^2^* = 0%). These findings indicate that probiotic intervention length did not influence gut microbiota diversity (Additional file Fig. S12).

### Small study publication bias and leave-one-out analysis

Small study publication bias assessment and leave-out analyses did not raise serious concerns and did not find influential studies (Additional file Figs. S13–20).

### The impact of probiotic supplementation on the gut microbiome α-diversity in generally healthy populations

Most of the included studies reported no significant and relevant effect or difference in α-diversity values following probiotic consumption compared to the control group. A detailed summary of the results, including specific indices assessed in each article, is presented in Additional file Table S4.

Shi et al. (2020) found a decrease in Shannon and Simpson’s index of diversity after the multi-strain probiotic intervention, while the control group microbiota remained stable. There was no difference in the number of observed OTUs in either of the groups [96]. Plaza-Diaz et al. investigated several bacterial strains, comparing them to a placebo group. In their study, treatments with *Lacticaseibacillus paracasei* CNCM I-4034 or *Lacticaseibacillus rhamnosus* CNCM I-4036 significantly increased the Shannon index at the end of the intervention. At the same time, *Bifidobacterium breve* CNCM I-4035 or the combined supplementation of *B. breve* CNCM I-4035 and *L. rhamnosus* CNCM I-4036 did not affect α-diversity. Notably, the results of the placebo group have not been mentioned in the article, and no comparison between the groups has been performed [91]. Rahayu et al. reported significant increases in Chao1 and observed OTUs in the *Lactiplantibacillus plantarum* Dad-13*-*supplemented group; however, a comparison with the placebo group was not performed [93]. According to our comparative meta-analysis, the mean of median differences was not significantly nor relevantly different in the two groups after the intervention period.

Paytuvi-Gallart investigated the children population. According to their results*, Bacillus subtilis* DE111 significantly increased both Shannon and Simpson’s index of diversity α-diversity indices but not the richness of the gut microbiota. However, no comparison with the control group was performed [90]. Interestingly, Gan et al. reported an increase in alpha diversity in the stools of the placebo group among children; however, they did not specify which of the four investigated indices was exactly affected. The probiotic group consuming a multi-strain product did not change over time [69]. Investigating the infant population, Li et al. reported increased Simpson’s index of diversity, Chao1, and ACE indices after *L. paracasei* N1115 intake. Notably, the Shannon index was significantly higher in both the probiotic and control groups after the intervention [79].

All other studies with other probiotic strains reported no significant changes or differences in α-diversity indices [57–68, 70–72, 74–76, 78, 80, 81, 83–85, 87–89, 92, 94, 95, 97–103].

### The impact of probiotic supplementation on the gut microbiome β-diversity in generally healthy populations

Β-diversity analyses largely confirmed the absence of a significant and relevant effect of probiotics on altering the overall structure of the gut microbiota, with some exceptions. Detailed results are summarised in Additional file Table S5.

Ferrario et al. revealed that treatment with *L. paracasei* DG significantly altered the overall faecal microbiota composition of participants, as demonstrated by repeated-measures ANOVA of paired distances between the probiotic and placebo treatments based on weighted UniFrac distance [66]. Plaza-Diaz et al. found that probiotic treatment altered β-diversity over time in participants taking *L. rhamnosus*, based on weighted UniFrac distance. Participants in this group tended to have more similar overall structures after the intervention. This effect was still observed after 15 days of follow-up [91]. Similarly, Wischmeyer et al. found a significant difference in β-diversity based on the Bray–Curtis distance between the probiotic-supplemented (*L. rhamnosus*) and control groups [103]. Sohn et al. reported a significant difference between the control and the *L. plantarum* K50-supplemented group based on Bray–Curtis distance [99]. According to Majeed et al., significant differences in β-diversity between placebo and *Heyndrickxia coagulans*-supplemented groups using both weighted and unweighted UniFrac distances were observed.

In the study of Gan et al., the microbiota of the children in the multi-strain probiotic group remained stable when comparing weeks in contrast to the placebo group, which displayed significant variability across the same time points based on weighted UniFrac distance [69]. On the other hand, Lau et al. reported a significant change in Bray–Curtis distance in the *Bifidobacterium longum* BB536-supplemented children after the treatment [77].

All the other studies with other probiotic strains did not report any significant change or difference in β-diversity indices [58–64, 67, 68, 70–72, 74, 75, 78, 79, 82, 83, 85–89, 92, 95, 97, 98, 101, 102].

### Risk of bias assessment

The risk of bias assessment indicated that 11 studies were judged to have low risk of bias, 9 had some concerns, and 11 were rated as high risk (see Additional File Tables S6–S7 and Additional file Figs. S21–24). The two cross-over studies were both classified as high risk of bias. Subgroup analysis based on risk of bias categories revealed no statistically significant differences between groups, suggesting that the overall findings were not materially influenced by study quality.

### Grade assessment

Based on the GRADE assessment, the quality of evidence for the meta-analyses was rated as low for the Shannon, observed OTUs, and Chao1 indices, and moderate for Simpson’s index of diversity (Additional file Table S8). The evidence was primarily downgraded for risk of bias, reflecting methodological limitations in several included trials, and for imprecision, as the pooled confidence intervals crossed the null value, indicating uncertainty in the true effect estimate (Additional file Table S8).

## Discussion

This study provides the first quantitative synthesis of randomised controlled trials examining the effect of probiotic supplementation on gut microbiota diversity in healthy populations. Overall, probiotics did not produce statistically significant changes in microbiota diversity, suggesting that diversity indices remain stable following supplementation in healthy individuals. It is important to note that the absence of significant changes in α- or β-diversity does not imply a lack of probiotic efficacy. Many probiotic effects may be mediated through transient interactions, metabolic modulation, or immunological mechanisms rather than broad compositional shifts. The present study aimed solely to assess whether such diversity changes have been consistently observed in healthy populations, without assuming that diversity itself serves as the primary marker of probiotic effectiveness. It is also important to note that microbiota diversity alone should not be considered a direct indicator of health. In this study, diversity metrics were evaluated as commonly reported microbiome parameters rather than as proxies for health status, and their interpretation should always be contextualised.

Our findings align with those of the systematic review by Kristensen et al. in 2016 [104], which similarly found minimal changes in gut microbiota composition in healthy adults, while more recent studies enabled us to provide a more comprehensive and up-to-date review. Unlike previous reviews, we conducted a meta-analysis and categorised studies by probiotic taxonomic families to partly address protocol heterogeneity.

Most included studies reported no statistically significant differences in α- or β-diversity between probiotic-supplemented and control groups though inconsistencies exist due to strain differences and age variations, highlighting the complexity of probiotic effects. These results align with our recent systematic review and meta-analysis, which demonstrated that probiotics are ineffective in preserving gut microbiota diversity even during antibiotic therapy [42]. Furthermore, probiotic supplementation has been shown to be ineffective in preventing *Clostridioides difficile* infection [105] and modifying zonulin levels in healthy populations [106].

### Strain-specific contradictions and similarities

Historically, *Lactobacillus* and *Bifidobacterium* species have the probiotic landscape due to their safety and believed health benefits [107]. The 2020 reclassification of *Lactobacillus* in 2020 into 23 genera underscored their genetic and functional diversity highlighting the need for strain-specific research [107, 108]. We updated bacterial strain names based on the NCBI Taxonomy Database for consistency [109]. Other genera, like *Bacillus*, have gained attention for their unique properties, such as spore formation and improved gastrointestinal survivability [107]. Our sub-group meta-analysis, based on taxonomic families, found no statistically significant effect on gut microbiota diversity in healthy populations. However, strain-level analysis highlighted notable differences, emphasising the importance of specificity in probiotic research.

Ferrario et al. reported that *L. paracasei* DG significantly altered β-, but not α-diversity [66], while Plaza-Diaz et al. and Li et al. observed changes in α-diversity with *L. paracasei* CNCM I-4034 and *L. paracasei N1115* but no corresponding changes in β-diversity [79, 91].

While Rahayu et al. found significant increases in Chao1 and observed OTUs with *Lactiplantibacillus plantarum* Dad-13, other studies using *L. plantarum* K50, LPQ180, or IMC 510® reported no changes in α-diversity [88, 89, 99]. This aligns with our meta-analysis finding no significant difference in the Rahayu et al. study when performing a comparison to a placebo. Notably, however, there was a significant difference in the overall composition based on Bray–Curtis distance in the study of Sohn et al. using *L. plantarum* K50 [99].

Supplementation with *Lacticaseibacillus rhamnosus* CNCM I-4036[91] and *L. rhamnosus GG* (ATCC 53103) [103] resulted in changes in α- and/or β-diversity over time, while another study using *L. rhamnosus* LRa05 did not report significant changes in either α- or β-diversity [68].

The conflicting results may be attributed to differences in variant-specific properties, doses, or study populations. On the other hand, other species investigated from the *Lactobacillaceae* family, such as *Lactiplantibacillus pentosus* [80], *Lactobacillus helveticus* [65], *Ligilactobacillus salivarius* [57, 64], *Lactobacillus johnsonii* [82], and *Limosilactobacillus reuteri* [98] showed concordant ineffectiveness in modulating microbiota diversity. These species are, however, less represented in our review.

We observed a contradictory effect in the *Bifidobacteriaceae* family, especially for *Bifidobacterium longum* BB536. Lau et al. found significant β-diversity changes (Bray–Curtis distance) in children [77], but no such effects in adults, according to Nakamura et al., were reported [87]. Similarly, another variant, *B. longum* BB68S, showed no significant changes in β-diversity in elderly participants [97]. Notably, α-diversity was not affected in the above studies, along with Moloney et al., who used *B. longum* AH1714 as probiotic supplementation [84].

This incongruency may be due to differences in specific bacterial formulations or host responses. However, other species from the taxonomic family, such as *Bifidobacterium animalis* [62, 64, 72], *Bifidobacterium bifidum* [65, 70],* Bifidobacterium breve* [85, 86, 91], and *B. longum subsp. infantis* [65, 73, 102], remained concordantly ineffective in modifying diversity indices compared to placebo across several studies in this review.

The *Bacillaceae* family also showed variable effects. Paytuvi-Gallart reported increases in Shannon and Simpson’s index of diversity indices with *Bacillus subtilis* DE111 in children [90]; however, in adults, another study using the same variant reported no significant changes in diversity metrics [67]. Similarly, *B. subtilis* R0179 was not effective, according to another study [71].

Majeed et al. observed significant changes in β-diversity with *Heyndrickxia coagulans* [81], while Kang et al. found no such effects with *H. coagulans* SNZ 1969.

Interestingly, multiple studies that used multistrain probiotic formulations have no statistically significant effect on α- and β-diversity indices [58–60, 63, 69, 74, 76, 78, 92, 94, 95, 98, 100, 101]. According to the study by Shi et al. (2020), Shannon and Simpson’s index of diversity was significantly lower than the control group after the intervention [96].

### Age group differences

Probiotic effects on diversity also varied across age groups, with children showing more pronounced responses in some cases. Li et al. reported increases in multiple α-diversity indices (Simpson’s index of diversity, Chao1, ACE) in infants supplemented with *L. paracasei* N1115 [79], while Paytuvi-Gallart found increased Shannon and Simpson’s index of diversity indices in children with *B. subtilis DE111* [90]. Moreover, Lau et al. observed significant β-diversity changes in children supplemented with *B. longum* BB536 [77]. These findings suggest that the developing microbiome in younger individuals may be more susceptible to probiotic-induced changes. On the other hand, most other studies did not reveal the modifying effect of probiotics in infants[59, 62, 64, 65, 73] or children [69].

Most adult studies reported no significant changes in α- or β-diversity indices. Similarly, studies focusing primarily on elderlies found no significant changes in diversity metrics [76, 95, 97]. This aligns with the hypothesis that a mature, stable microbiome is less responsive to probiotic interventions [110, 111].

### Implication for practice and research

The synthesis of the available literature supports applying scientific findings in everyday practice, which is critically important [112, 113]. Probiotic supplementation has a limited effect on the diversity of the gut microbiome in healthy individuals. Personalised recommendations, considering individual factors and the benefits of each strain, would be essential. In clinical practice, probiotics should be used selectively, focusing on functional benefits and targeted use in vulnerable populations. Probiotic use in healthy populations should emphasise evidence-based functional outcomes rather than microbiota diversity changes. Educating patients about the limitations and potential benefits of probiotics is essential.

Future research should standardise diversity assessment methods, consider functional and clinical outcomes alongside diversity metrics, explore strain-specific mechanisms, and evaluate long-term effects. Aligning diversity data with broader health outcomes will provide a clearer understanding of the role of probiotics in promoting gut and overall health. Combining microbiome data with metagenomics, metabolomics, and transcriptomics could provide a more comprehensive understanding of probiotic effects.

### Strengths and limitations

Our systematic review and meta-analysis provide the highest level of evidence, including only randomised controlled trials. To our knowledge, this is the first study conducting both qualitative and quantitative synthesis on this topic. We followed Cochrane’s recommendations and the PRISMA Statement with strict methodology.

Despite including all relevant studies without restrictions on microbial variables, quantitative synthesis was limited due to inconsistent reporting and methodological variability. Differences in probiotic strains and dosing regimens prevented strain-level meta-analysis, so subgroup analyses were conducted at the family level. Healthy, but still heterogeneous populations and age variations further complicate interpretation. These limitations highlight the need for standardised methodologies to clarify probiotic effects. Our meta-analysis focused solely on microbiota diversity, excluding functional effects and health outcomes.

Future research should explore these aspects to provide a comprehensive understanding of the broader effects of probiotics.

No race or ethnicity-based analyses were carried out. Although we consider such analyses to be very important, as studies have shown that there can be differences in the response of races and ethnicities to a given treatment, at the same time, the available publications only provide data in this breakdown in a very limited number of cases, which makes it impossible to carry out a comprehensive analysis.

## Conclusions

The summarised results from the currently available randomised controlled trials do not support probiotic supplementation as an effective strategy to modify gut microbiota diversity in healthy populations. Meta-analyses of the most common diversity indices, including Shannon, Chao1, observed OTUs, and Simpson’s index of diversity, revealed no significant effect of probiotics on modulating or increasing microbiota diversity. While not all reported outcomes could be analysed quantitatively, the strong overall trend across studies suggests a lack of influencing effect on both α- and β-diversity metrics.

There is a strong need for standardised normal ranges and consistent reporting of diversity metrics to support more robust and comparable analyses. A consensus for appropriate methods and clinically important outcomes is critical for further research. Studies should focus on the potential clinical relevance of probiotics in specific populations and on understanding the functional impacts of microbiota modulation.

## Supplementary Information

Additional file1: Table S1 PRISMA checklist 2020. Table S2 Search key. Table S3 Definitions of gut microbiota diversity outcomes reported in the included studies. Supplementary Methods S1 Detailed statistical description of the meta-analysis. Fig. S1 Additional sensitivity analysis for the more restricted analysis of Shannon diversity index. Fig. S2 Additional sensitivity analysis for the subgroups based on risk of bias assessment for Shannon diversity index. Fig. S3 Meta-regression analysis investigating the relationship between intervention duration and the Shannon diversity index. Fig. S4 Additional sensitivity analysis for the more restricted analysis of Observed OTUs. Fig. S5 Additional sensitivity analysis for the subgroups based on risk of bias assessment for Observed OTUs. Fig. S6 Meta-regression analysis investigating the relationship between intervention duration and the number of Observed OTUs. Fig. S7 Additional sensitivity analysis for the more restricted analysis Chao1 index. Fig. S8 Additional sensitivity analysis for the subgroups based on the risk of bias assessment Chao1 index. Fig. S9 Meta-regression analysis investigating the relationship between intervention duration and the Chao1 index. Fig. S10 Additional sensitivity analysis for the more restricted analysis of Simpson’s Index of Diversity. Fig. S11 Additional sensitivity analysis for the subgroups based on risk of bias assessment of Simpson’s Index of Diversity. Fig. S12 Meta-regression analysis investigating the relationship between intervention duration and the Simpson’s Index of Diversity. Fig. S13 Funnel plot to assess publication bias for Shannon diversity index. Fig. S14 Additional leave-one-out analysis for Shannon diversity index. Fig. S15 Funnel plot to assess publication bias for the Observed Operational Taxonomic Units. Fig. S16 Additional leave-one-out analysis for the Observed Operational Taxonomic Units. Fig. S17 Funnel plot to assess publication bias for Chao1 index. Fig. S18 Additional leave-one-out analysis for Chao1 index. Fig. S19 Funnel plot to assess publication bias for Simpson’s Index of Diversity. Fig. S20 Additional leave-one-out analysis Simpson’s Index of Diversity. Table S4 Changes in the microbiome α-diversity indices as measured after the intervention period. Table S5 Changes in the microbiome β-diversity indices as measured after the intervention period. Table S6 Risk of bias assessment for parallel design studies. Table S7 Risk of bias assessment for cross-over design studies Fig. S21 Risk of bias assessment for parallel design studies - Assignment to intervention. Fig. S22 Risk of bias assessment for parallel design studies - Adhering to intervention. Fig. S23 Risk of bias assessment for cross-over design studies - Assignment to intervention. Fig. S24 Risk of bias assessment for crossover design studies - Adhering to intervention. Table S8 GRADE assessment for the meta-analyses of Shannon, Observed OTUs, Chao1 and Simpson’s Index of Diversity indices.

## Data Availability

All datasets used in this study can be found in the full-text publications included in the systematic review and meta-analysis. The detailed study protocol including the search key can be found in the PROSPERO registration (CRD42022286137) and the Additional file of the publication.

## Abbreviations

CI: Confidence interval
GRADE: Grading of Recommendations, Assessment, Development, and Evaluation
MedD: Means of median differences
OTU: Operational taxonomic unit
RCT: Randomised controlled trial
RoB: Risk of bias
PRISMA: Preferred Reporting Items for Systematic Reviews and Meta-Analyses
PROSPERO: International Prospective Register of Systematic Reviews
