# Supplementary material for: Effect of probiotic supplementation on the gut microbiota diversity in healthy populations: a systematic review and meta-analysis of randomised controlled trials
Source: BMC Med. 2026 Jan 7;24:71. doi: 10.1186/s12916-025-04602-0 (PMC12870995; doi:10.1186/s12916-025-04602-0)
Supplement: Supplementary file 1 — Additional file1: Table S1 PRISMA checklist 2020. Table S2 Search key. Table S3 Definitions of gut microbiota diversity outcomes reported in the included studies. Supplementary Methods S1 Detailed statistical description of the meta-analysis. Fig. S1 Additional sensitivity analysis for the more restricted analysis of Shannon diversity index. Fig. S2 Additional sensitivity analysis for the subgroups based on risk of bias assessment for Shannon diversity index. Fig. S3 Meta-regression analysis investigating the relationship between intervention duration and the Shannon diversity index. Fig. S4 Additional sensitivity analysis for the more restricted analysis of Observed OTUs. Fig. S5 Additional sensitivity analysis for the subgroups based on risk of bias assessment for Observed OTUs. Fig. S6 Meta-regression analysis investigating the relationship between intervention duration and the number of Observed OTUs. Fig. S7 Additional sensitivity analysis for the more restricted analysis Chao1 index. Fig. S8 Additional sensitivity analysis for the subgroups based on the risk of bias assessment Chao1 index. Fig. S9 Meta-regression analysis investigating the relationship between intervention duration and the Chao1 index. Fig. S10 Additional sensitivity analysis for the more restricted analysis of Simpson’s Index of Diversity. Fig. S11 Additional sensitivity analysis for the subgroups based on risk of bias assessment of Simpson’s Index of Diversity. Fig. S12 Meta-regression analysis investigating the relationship between intervention duration and the Simpson’s Index of Diversity. Fig. S13 Funnel plot to assess publication bias for Shannon diversity index. Fig. S14 Additional leave-one-out analysis for Shannon diversity index. Fig. S15 Funnel plot to assess publication bias for the Observed Operational Taxonomic Units. Fig. S16 Additional leave-one-out analysis for the Observed Operational Taxonomic Units. Fig. S17 Funnel plot to assess publication bias for Chao1 index. Fig. S18 A [file 12916_2025_4602_MOESM1_ESM.pdf]

Additional file

Contents

Supplementary Methods ... 3

Table S1 PRISMA checklist 2020 ... 3

Table S2 Search key ... 7

Table S3 Definitions of gut microbiota diversity outcomes reported in the included studies. ... 11

Supplementary Methods S1 Detailed statistical description of the meta-analysis ... 14

Supplementary Results ... 16

Figs. S1-3 Analyses of the Shannon diversity index – Sensitivity analysis, subgroups by risk of bias and meta-regression ... 16

Fig. S1 Additional sensitivity analysis for the more restricted analysis of Shannon diversity index. ... 16

Fig. S2 Additional sensitivity analysis for the subgroups based on risk of bias assessment for Shannon diversity index. ... 17

Fig. S3 Meta-regression analysis investigating the relationship between intervention duration and the Shannon diversity index. ... 18

Figs. S4-6 Analyses of the Observed OTUs diversity index – Sensitivity analysis, subgroups by risk of bias and meta-regression ... 19

Fig. S4 Additional sensitivity analysis for the more restricted analysis of Observed OTUs. ... 19

Fig. S5 Additional sensitivity analysis for the subgroups based on risk of bias assessment for Observed OTUs. ... 20

Fig. S6 Meta-regression analysis investigating the relationship between intervention duration and the number of Observed OTUs ... 21

Figs. S7-9 Analyses of the Chao1 index – Sensitivity analysis, subgroups by risk of bias and meta-regression ... 22

Fig. S7 Additional sensitivity analysis for the more restricted analysis Chao1 index. ... 22

Fig. S8 Additional sensitivity analysis for the subgroups based on the risk of bias assessment Chao1 index. ... 23

Fig. S9 Meta-regression analysis investigating the relationship between intervention duration and the Chao1 index. ... 24

Figs. S10-12 Analyses of the Simpson's Index of Diversity - Sensitivity analysis, subgroups by risk of bias and meta-regression ... 25

Fig. S10 Additional sensitivity analysis for the more restricted analysis of Simpson's Index of Diversity. ... 25

Fig. S11 Additional sensitivity analysis for the subgroups based on risk of bias assessment of Simpson's Index of Diversity. ... 26

Fig. S12 Meta-regression analysis investigating the relationship between intervention duration and the Simpson's Index of Diversity 27

Figs. S13-20 Publication bias and leave-out analyses 28

Fig. S13 Funnel plot to assess publication bias for Shannon diversity index. 28

Fig. S14 Additional leave-one-out analysis for Shannon diversity index. 29

Fig. S15 Funnel plot to assess publication bias for the Observed Operational Taxonomic Units (OTUs) 30

Fig. S16 Additional leave-one-out analysis for the Observed Operational Taxonomic Units (OTUs) 31

Fig. S17 Funnel plot to assess publication bias for Chao1 index. 32

Fig. S18 Additional leave-one-out analysis for Chao1 index. 33

Fig. S19 Funnel plot to assess publication bias for Simpson's Index of Diversity. 34

Fig. S20 Additional leave-one-out analysis Simpson's Index of Diversity. 35

Table S4 Changes in the microbiome α-diversity indices as measured after the intervention period. . 36

Table S5 Changes in the microbiome β-diversity indices as measured after the intervention period. . 42

Tables S6-7 Risk of bias assessments 47

Table S6 Risk of bias assessment for parallel design studies. 47

Table S7 Risk of bias assessment for cross-over design studies 49

Figs. S21-24 Risk of bias assessments 50

Fig. S21 Risk of bias assessment for parallel design studies - Assignment to intervention (the 'intention-to-treat' effect). 50

Fig. S22 Risk of bias assessment for parallel design studies - Adhering to intervention (the 'per-protocol' effect) 51

Fig. S23 Risk of bias assessment for cross-over design studies - Assignment to intervention (the 'intention-to-treat' effect). 52

Fig. S24 Risk of bias assessment for crossover design studies - Adhering to intervention (the 'per-protocol' effect) 53

Table S8 GRADE assessment for the meta-analyses of Shannon, Observed OTUs, Chao1 and Simpson's Index of Diversity indices 54

References to Supplementary material 57

Supplementary Methods

Table S1 PRISMA checklist 2020

Section and Topic	Item #	Checklist item	Location where item is reported
**TITLE**			
Title	1	Identify the report as a systematic review.	Title page
**ABSTRACT**			
Abstract	2	See the PRISMA 2020 for Abstracts checklist.	Page 3
**INTRODUCTION**			
Rationale	3	Describe the rationale for the review in the context of existing knowledge.	Page 4-5
Objectives	4	Provide an explicit statement of the objective(s) or question(s) the review addresses.	Page 5
**METHODS**			
Eligibility criteria	5	Specify the inclusion and exclusion criteria for the review and how studies were grouped for the syntheses.	Page 5-6
Information sources	6	Specify all databases, registers, websites, organisations, reference lists and other sources searched or consulted to identify studies. Specify the date when each source was last searched or consulted.	Page 6
Search strategy	7	Present the full search strategies for all databases, registers, and websites, including any filters and limits used.	Page 5-6 and S2 Table
Selection process	8	Specify the methods used to decide whether a study met the inclusion criteria of the review, including how many reviewers screened each record and each report retrieved, whether they worked independently, and if applicable, details of automation tools used in the process.	Page 5-8 and Page 9 (Figure 1)
Data collection process	9	Specify the methods used to collect data from reports, including how many reviewers collected data from each report, whether they worked independently, any processes for obtaining or confirming data from study investigators, and if applicable, details of automation tools used in the process.	Page 6-7
Data items	10a	List and define all outcomes for which data were sought. Specify whether all results that were compatible with each outcome domain in each study were sought (e.g., for all measures, time points, analyses), and if not, the methods used to decide which results to collect.	Page 5-7
	10b	List and define all other variables for which data were sought (e.g., participant and intervention characteristics, funding sources). Describe any assumptions made about any missing or unclear information.	Page 6-7 and Page 10-13 (Table 1)
Study risk of bias assessment	11	Specify the methods used to assess risk of bias in the included studies, including details of the tool(s) used, how many reviewers assessed each study and whether they worked independently, and if applicable, details of automation tools used in the process.	Page 7 and Supplementary Methods
Effect measures	12	Specify for each outcome the effect measure(s) (e.g., risk ratio, mean difference) used in the synthesis or presentation of results.	Page 7 and

Section and Topic	Item #	Checklist item	Location where item is reported
			Supplementary Methods
Synthesis methods	13a	Describe the processes used to decide which studies were eligible for each synthesis (e.g., tabulating the study intervention characteristics and comparing against the planned groups for each synthesis (item #5)).	Page 7 and Supplementary Methods
	13b	Describe any methods required to prepare the data for presentation or synthesis, such as handling of missing summary statistics, or data conversions.	Page 7 and Supplementary Methods
	13c	Describe any methods used to tabulate or visually display results of individual studies and syntheses.	Page 7 and Supplementary Methods
	13d	Describe any methods used to synthesize results and provide a rationale for the choice(s). If meta-analysis was performed, describe the model(s), method(s) to identify the presence and extent of statistical heterogeneity, and software package(s) used.	Page 7 and Supplementary Methods
	13e	Describe any methods used to explore possible causes of heterogeneity among study results (e.g., subgroup analysis, meta-regression).	Page 7 and Supplementary Methods
	13f	Describe any sensitivity analyses conducted to assess robustness of the synthesized results.	Page 7 and Supplementary Methods
Reporting bias assessment	14	Describe any methods used to assess risk of bias due to missing results in a synthesis (arising from reporting biases).	Page 7 and Supplementary Methods
Certainty assessment	15	Describe any methods used to assess certainty (or confidence) in the body of evidence for an outcome.	Page 7
**RESULTS**			
Study selection	16a	Describe the results of the search and selection process, from the number of records identified in the search to the number of studies included in the review, ideally using a flow diagram.	Page 8-9, (Figure 1)
	16b	Cite studies that might appear to meet the inclusion criteria, but which were excluded, and explain why they were excluded.	Page 8-9, (Figure 1) and Supplementary Methods
Study characteristics	17	Cite each included study and present its characteristics.	Page 10-13 (Table 1)

Section and Topic	Item #	Checklist item	Location where item is reported
Risk of bias in studies	18	Present assessments of risk of bias for each included study.	Page 20 and S5-7 Tables and S16-18 Figure
Results of individual studies	19	For all outcomes, present, for each study: (a) summary statistics for each group (where appropriate) and (b) an effect estimate and its precision (e.g. confidence/credible interval), ideally using structured tables or plots.	Page 14-18 Figure 2-7 and S1-9 Figure and S3 Table
Results of syntheses	20a	For each synthesis, briefly summarise the characteristics and risk of bias among contributing studies.	Page 20 and S5-7 Tables and S16-18 Figure
	20b	Present results of all statistical syntheses conducted. If meta-analysis was done, present for each the summary estimate and its precision (e.g., confidence/credible interval) and measures of statistical heterogeneity. If comparing groups, describe the direction of the effect.	Page 14-18 Figure 2-7 and S1-9 Figure
	20c	Present results of all investigations of possible causes of heterogeneity among study results.	Page 18 and S10-15 Figure
	20d	Present results of all sensitivity analyses conducted to assess the robustness of the synthesized results.	Page 18 and S1-9 Figure
Reporting biases	21	Present assessments of risk of bias due to missing results (arising from reporting biases) for each synthesis assessed.	Page 20 and S5-7 Tables and S16-18 Figure
Certainty of evidence	22	Present assessments of certainty (or confidence) in the body of evidence for each outcome assessed.	Page 20 and S8 Table
**DISCUSSION**			
Discussion	23a	Provide a general interpretation of the results in the context of other evidence.	Page 20-22
	23b	Discuss any limitations of the evidence included in the review.	Page 23
	23c	Discuss any limitations of the review processes used.	Page 23
	23d	Discuss implications of the results for practice, policy, and future research.	Page 23
**OTHER INFORMATION**			
Registration and protocol	24a	Provide registration information for the review, including register name and registration number, or state that the review was not registered.	Page 5
	24b	Indicate where the review protocol can be accessed, or state that a protocol was not prepared.	Page 5

Section and Topic	Item #	Checklist item	Location where item is reported
	24c	Describe and explain any amendments to information provided at registration or in the protocol.	Page 5
Support	25	Describe sources of financial or non-financial support for the review, and the role of the funders or sponsors in the review.	Title Page (Page 2)
Competing interests	26	Declare any competing interests of review authors.	Title Page (Page 2)
Availability of data, code and other materials	27	Report which of the following are publicly available and where they can be found: template data collection forms; data extracted from included studies; data used for all analyses; analytic code; any other materials used in the review.	Title Page (Page 2)

Table S2 Search key

**Search key**	(normal OR general OR healthy) **AND** (population OR participant OR participants OR volunteer OR volunteers OR subject OR subjects OR adult OR adults OR adolescent OR adolescents OR child OR children OR infant OR infants OR newborn OR newborns OR birth cohort OR pediatric* OR elderly OR elders) **AND** (probiotic OR probiotic* OR bifidobac* OR lactobac* OR escherichia OR streptococcus OR saccharomyces OR bacillus OR pediococc* OR leuconostoc) **AND** random*
**MEDLINE (Expanded)**	("normal"[All Fields] OR "normalisation"[All Fields] OR "normalisations"[All Fields] OR "normalise"[All Fields] OR "normalised"[All Fields] OR "normalises"[All Fields] OR "normalising"[All Fields] OR "normalization"[All Fields] OR "normalizations"[All Fields] OR "normalize"[All Fields] OR "normalized"[All Fields] OR "normalizer"[All Fields] OR "normalizers"[All Fields] OR "normalizes"[All Fields] OR "normalizing"[All Fields] OR "normally"[All Fields] OR "normals"[All Fields] OR ("drugs, generic"[MeSH Terms] OR ("drugs"[All Fields] AND "generic"[All Fields]) OR "generic drugs"[All Fields] OR "generic"[All Fields] OR "family characteristics"[MeSH Terms] OR ("family"[All Fields] AND "characteristics"[All Fields]) OR "family characteristics"[All Fields] OR "generation"[All Fields] OR "generations"[All Fields] OR "general"[All Fields] OR "general s"[All Fields] OR "generalisability"[All Fields] OR "generalisable"[All Fields] OR "generalisation"[All Fields] OR "generalization, psychological"[MeSH Terms] OR ("generalization"[All Fields] AND "psychological"[All Fields]) OR "psychological generalization"[All Fields] OR "generalization"[All Fields] OR "generalisations"[All Fields] OR "generalise"[All Fields] OR "generalised"[All Fields] OR "generalises"[All Fields] OR "generalisibility"[All Fields] OR "generalising"[All Fields] OR "generalities"[All Fields] OR "generality"[All Fields] OR "generalizability"[All Fields] OR "generalizable"[All Fields] OR "generalizations"[All Fields] OR "generalize"[All Fields] OR "generalized"[All Fields] OR "generalizes"[All Fields] OR "generalizing"[All Fields] OR "generally"[All Fields] OR "generals"[All Fields] OR "generate"[All Fields] OR

	"generated"[All Fields] OR "generates"[All Fields] OR "generating"[All Fields] OR "generation s"[All Fields] OR "generational"[All Fields] OR "generative"[All Fields] OR "generatively"[All Fields] OR "generativity"[All Fields] OR "generator"[All Fields] OR "generator s"[All Fields] OR "generators"[All Fields] OR "generically"[All Fields] OR "genericity"[All Fields] OR "generics"[All Fields]) OR ("healthies"[All Fields] OR "healthy"[All Fields])) AND ("populate"[All Fields] OR "populated"[All Fields] OR "populates"[All Fields] OR "populating"[All Fields] OR "population"[MeSH Terms] OR "population"[All Fields] OR "population groups"[MeSH Terms] OR ("population"[All Fields] AND "groups"[All Fields]) OR "population groups"[All Fields] OR "populations"[All Fields] OR "population s"[All Fields] OR "populational"[All Fields] OR "populous"[All Fields] OR ("participant"[All Fields] OR "participant s"[All Fields] OR "participants"[All Fields] OR "participate"[All Fields] OR "participated"[All Fields] OR "participates"[All Fields] OR "participating"[All Fields] OR "participation"[All Fields] OR "participations"[All Fields] OR "participative"[All Fields] OR "participator"[All Fields] OR "participators"[All Fields]) OR ("participant"[All Fields] OR "participant s"[All Fields] OR "participants"[All Fields] OR "participate"[All Fields] OR "participated"[All Fields] OR "participates"[All Fields] OR "participating"[All Fields] OR "participation"[All Fields] OR "participations"[All Fields] OR "participative"[All Fields] OR "participator"[All Fields] OR "participators"[All Fields]) OR ("volunteer s"[All Fields] OR "volunteered"[All Fields] OR "volunteers"[MeSH Terms] OR "volunteers"[All Fields] OR "volunteer"[All Fields] OR "volunteering"[All Fields]) OR ("volunteer s"[All Fields] OR "volunteered"[All Fields] OR "volunteers"[MeSH Terms] OR "volunteers"[All Fields] OR "volunteer"[All Fields] OR "volunteering"[All Fields]) OR ("subject"[All Fields] OR "subject s"[All Fields] OR "subjects"[All Fields] OR "subjects s"[All Fields]) OR ("subject"[All Fields] OR "subject s"[All Fields] OR "subjects"[All Fields] OR "subjects s"[All Fields]) OR ("adult"[MeSH Terms] OR "adult"[All Fields] OR "adults"[All Fields] OR "adult s"[All Fields]) OR ("adult"[MeSH Terms] OR "adult"[All Fields] OR "adults"[All Fields] OR "adult s"[All Fields]) OR ("adolescences"[All Fields] OR

	"adolescency"[All Fields] OR "adolescent"[MeSH Terms] OR "adolescent"[All Fields] OR "adolescence"[All Fields] OR "adolescents"[All Fields] OR "adolescent s"[All Fields]) OR ("adolescences"[All Fields] OR "adolescency"[All Fields] OR "adolescent"[MeSH Terms] OR "adolescent"[All Fields] OR "adolescence"[All Fields] OR "adolescents"[All Fields] OR "adolescent s"[All Fields]) OR ("child"[MeSH Terms] OR "child"[All Fields] OR "children"[All Fields] OR "child s"[All Fields] OR "children s"[All Fields] OR "childrens"[All Fields] OR "childs"[All Fields]) OR ("child"[MeSH Terms] OR "child"[All Fields] OR "children"[All Fields] OR "child s"[All Fields] OR "children s"[All Fields] OR "childrens"[All Fields] OR "childs"[All Fields]) OR ("infant"[MeSH Terms] OR "infant"[All Fields] OR "infants"[All Fields] OR "infant s"[All Fields]) OR ("infant"[MeSH Terms] OR "infant"[All Fields] OR "infants"[All Fields] OR "infant s"[All Fields]) OR ("infant, newborn"[MeSH Terms] OR ("infant"[All Fields] AND "newborn"[All Fields]) OR "newborn infant"[All Fields] OR "newborn"[All Fields] OR "newborns"[All Fields] OR "newborn s"[All Fields]) OR ("infant, newborn"[MeSH Terms] OR ("infant"[All Fields] AND "newborn"[All Fields]) OR "newborn infant"[All Fields] OR "newborn"[All Fields] OR "newborns"[All Fields] OR "newborn s"[All Fields]) OR ("birth cohort"[MeSH Terms] OR ("birth"[All Fields] AND "cohort"[All Fields]) OR "birth cohort"[All Fields]) OR "pediatric*"[All Fields] OR ("aged"[MeSH Terms] OR "aged"[All Fields] OR "elderly"[All Fields] OR "elderlies"[All Fields] OR "elderly s"[All Fields] OR "elderlys"[All Fields]) OR ("elder s"[All Fields] OR "elders"[All Fields] OR "sambucus"[MeSH Terms] OR "sambucus"[All Fields] OR "elder"[All Fields])) AND ("probiotic s"[All Fields] OR "probiotical"[All Fields] OR "probiotics"[MeSH Terms] OR "probiotics"[All Fields] OR "probiotic"[All Fields] OR "probiotic*"[All Fields] OR "bifidobac*"[All Fields] OR "lactobac*"[All Fields] OR ("escherichia"[MeSH Terms] OR "escherichia"[All Fields] OR "escherichiae"[All Fields]) OR ("streptococcus"[MeSH Terms] OR "streptococcus"[All Fields]) OR ("saccharomyces"[MeSH Terms] OR "saccharomyces"[All Fields] OR "saccharomyce"[All Fields]) OR ("bacillu"[All Fields] OR "bacillus"[MeSH Terms]

	OR "bacillus"[All Fields]) OR "pediococc*"[All Fields] OR ("leuconostoc"[MeSH Terms] OR "leuconostoc"[All Fields] OR "leuconostocs"[All Fields])) AND "random*"[All Fields]
**EMBASE (Expanded)**	(normal OR general OR healthy) AND ('population'/exp OR population OR participant OR participants OR 'volunteer'/exp OR volunteer OR 'volunteers'/exp OR volunteers OR subject OR subjects OR 'adult'/exp OR adult OR 'adults'/exp OR adults OR 'adolescent'/exp OR adolescent OR 'adolescents'/exp OR adolescents OR 'child'/exp OR child OR 'children'/exp OR children OR 'infant'/exp OR infant OR 'infants'/exp OR infants OR 'newborn'/exp OR newborn OR newborns OR 'birth cohort'/exp OR 'birth cohort' OR (('birth'/exp OR birth) AND ('cohort'/exp OR cohort)) OR pediatric* OR 'elderly'/exp OR elderly OR elders) AND ('probiotic'/exp OR probiotic OR probiotic* OR bifidobac* OR lactobac* OR 'escherichia'/exp OR escherichia OR 'streptococcus'/exp OR streptococcus OR 'saccharomyces'/exp OR saccharomyces OR 'bacillus'/exp OR bacillus OR pediococc* OR 'leuconostoc'/exp OR leuconostoc) AND random*
**Cochrane Register of Controlled Trials (CENTRAL) (Expanded)**	(normal OR general OR healthy) AND (population OR participant OR participants OR volunteer OR volunteers OR subject OR subjects OR adult OR adults OR adolescent OR adolescents OR child OR children OR infant OR infants OR newborn OR newborns OR birth cohort OR pediatric* OR elderly OR elders) AND (probiotic OR probiotic* OR bifidobac* OR lactobac* OR escherichia OR streptococcus OR saccharomyces OR bacillus OR pediococc* OR leuconostoc) AND random*

Table S3 Definitions of gut microbiota diversity outcomes reported in the included studies.

α-diversity indices	Definition	Reference
Shannon diversity index	The Shannon diversity index shows how diverse the species in a given community are. It rises with the number of species and the evenness of their abundance. The higher the index is, the more diverse the species are in the habitat. If the index equals 0, only one species is present in the community. The index has no upper limit. According to the current data, there are no clearly defined reference values for the "ideal" Shannon diversity of the microbiome. For the definition of low diversity, the cut-off points in the literature range from 2.0 to 4.0.	[1–4]
Observed OTUs	An OTU table contains the number of sequences that are observed for each operational taxonomic unit (OTUs) in each sample. An OTU can be defined as a collection of 16S rRNA sequences that have a certain percentage of sequence divergence. Columns usually represent samples, and rows represent genera or species-specific taxonomic units (OTUs).	[4, 5]
Chao1 index	Chao1 is a nonparametric method for estimating the number of species in a community. The Chao richness estimator is based on the concept that rare species infer the most information about the number of missing species.	[4, 6]
PD whole tree / Faith Phylogenetic diversity	A quantitative measure of phylogenetic diversity, "PD", has been defined as the minimum total length of all the phylogenetic branches required to span a given set of taxa on the phylogenetic tree.	[4, 7]
Strong's dominance index	Strong's dominance index measures the maximum departure between the observed proportions and a perfectly even community.	[4, 8]
Pielou's evenness	Pielou's evenness is an index that measures diversity along with species richness. While species richness is the number of different species in a given area, evenness is the count of individuals of each species in an area. A calculated value of Pielou's evenness ranges from 0 (no evenness) to 1 (complete evenness).	[4, 9]
Sobs index	Sobs is the total number of species observed in a sample, or in a set of samples.	[4, 10]
ACE (Abundance-based coverage estimator) of species richness index	The ACE is a nonparametric method for estimating the number of species using sample coverage, which is defined as the sum of the probabilities of the observed species. By the ACE method the groups can be categorized as abundant and rare groups according to the observed frequencies.	[4, 11, 12]
Simpson index	Simpson's index is a weighted arithmetic mean of proportional abundance, used to measure the probability that two individuals randomly chosen from a sample belong to the same species. The index reflects both the richness (number of species) and evenness (distribution of individuals among species) within a sample. The value of the index (D) ranges between 0 (infinite diversity) and 1 (no diversity). To	[13–15]

	emphasize diversity, the index is often transformed into Gini-Simpson index, the Simpson's index of Diversity (1-D) which ranges from 0 (maximum homogeneity) to 1 (maximum diversity).	
Inverse Simpson index	This is the inverse of Simpson dominance and is often used to measure species diversity. It gives an estimate of the effective number of equally abundant species that would result in the same level of dominance. A higher Simpson reciprocal dominance value signifies higher species diversity or richness, with a more even distribution of individuals among species.	[15, 16]
Shannon effective count	The number of equally-common species required to give a particular value of an index is called the "effective number of species". It provides an intuitive interpretation of diversity as the equivalent number of equally abundant species in a community.	[14, 16, 17]
**β-diversity indices**	**Definition**	**Reference**
Bray-Curtis (dis)similarity index	The Bray–Curtis dissimilarity is bounded between 0 and 1, where 0 means that the two sites have the same composition (i.e., they share all the species), and 1 means that the two sites do not share any species. At sites where BC is intermediate (e.g., BC = 0.5), this index differs from other commonly used indices.	[4, 18]
Euclidean distance	When two samples are compared, Euclidean distance puts more weight on differences in species abundances than on difference in species presences. As a result, two samples not sharing any species could appear more similar (with lower Euclidean distance) than two samples which share species, but the species largely differ in their abundances	[4, 19]
(un)weighted UniFrac distance	Both weighted (quantitative) and unweighted (qualitative) variants of UniFrac are widely used in microbial ecology, where the former accounts for the abundance of observed organisms, while the latter only considers their presence or absence. The distance is calculated between pairs of samples (each sample represents an organismal community). All taxa found in one or both samples are placed on a phylogenetic tree. A branch leading to taxa from both samples is marked as "shared" and branches leading to taxa which appear only in one sample are marked as "unshared". There is a weighted version of the UniFrac metric, which accounts for the relative abundance of each of the taxa within the communities. This is commonly used in metagenomic studies, where the number of metagenomic reads can be in the tens of thousands, and it is appropriate to 'bin' these reads into operational taxonomic units, or OTUs, which can then be dealt with as taxa within the UniFrac framework.	[4, 20]
Generalized UniFrac distance	The generalized UniFrac distances unifies weighted UniFrac and unweighted UniFrac distances. It covers a series of distances ranging from weighted to unweighted UniFrac by adjusting the weight on the branches. This approach is designed to offer a robust and versatile tool for detecting a broader spectrum of biologically significant changes in microbiome composition.	[21]

Jensen-Shannon divergence	The Jensen-Shannon divergence is an asymmetric measure that quantifies the relative entropy or informational difference between two distributions. It provides a way to evaluate the distance between two data distributions, highlighting how distinct they are from one another.	[22]
Horn-Morisita distance metrics	The Horn-Morisita index evaluates the probability that individuals drawn from two separate vectors belong to different species, relative to drawing from each vector independently. It is applicable to both transformed counts and proportions.	[23]
Spearman correlation distance	The Spearman distance is based on the Spearman rank correlation coefficient, which evaluates the monotonic relationship between two variables. The Spearman distance is calculated as one minus the absolute value of the Spearman correlation coefficient, offering a measure of dissimilarity between ranked data. It is particularly useful when the data exhibit a monotonic association rather than a linear one.	

Abbreviations: *OTU:* operational taxonomic unit; *PD*: phylogenetic diversity; *ACE:* abundance-based coverage estimator; *UniFrac*: unique fraction metric

α-diversity indices reflect the diversity of a single sample, measuring species richness (number of species) and/or distribution (evenness of species). Each alpha diversity index is calculated differently, depending on factors like how the presence or absence of certain rare species is assessed and interpreted. In contrast, β-diversity indices can be used to compare different samples and communities. It can consider both the overall abundance per sample and the abundance of each taxon [24]. In simple terms, α-diversity represents a within-sample diversity, while β-diversity describes similarity or dissimilarity between samples [25–27].

Supplementary Methods S1 Detailed statistical description of the meta-analysis

As we assumed considerable between-study heterogeneity in all cases, a random-effects model was used to pool effect sizes in a frequentist framework.

In most studies, the quartiles of diversity index values could be extracted from box plots. Therefore, the effect size was expressed as the difference in medians (MedD) between groups (probiotic-treated minus control) instead of the usual mean difference. Based on the available data and our experience, we assume that the two are not significantly different. From the study MedD, we estimated the mean of median differences. Since only RCT studies were included, we used post-treatment data to estimate the difference between the treated and control groups.

Based on the available data (the symmetry of the lower and upper quartiles around the median, the similarity between the reported mean and median, and one study providing individual-level data), as well as our experience, it is a reasonable assumption that the distribution of diversity indices follows a normal distribution. Under this assumption, we estimated the differences in medians and their standard errors for each study using the quantile estimation method of the metamedian package [28], based on the available data (quartiles, as well as mean and standard deviation values). For the Lopez-Garcia study [29], individual-level data could be extracted from the plot, and the standard error was estimated using a bootstrap method.

We used the inverse variance weighting method for pooling MedDs. To estimate the heterogeneity variance measure τ2, the restricted maximum-likelihood estimator with the Q profile method for confidence interval, Veroniki et al. [30] was applied.

We used a Hartung-Knapp adjustment [31, 32] for CIs (if it is more conservative than the classical one, as recommended by Jackson et al.[33] as a hybrid method) and for prediction intervals. In case of subgroup (e.g., categorical moderator, as intervention type and categorized intervention time) analysis, we used a fixed-effects "plural" model (aka. mixed-effects model). We assumed that all subgroups share a different τ. Although for practical reasons, if at least one

of the subgroups contains fewer than 6 studies, we use an assumption of the same τ across subgroups (recommended in Harrer et al. [34]).

In the case of meta-regression (e.g., continuous moderator, as time of intervention) analysis, a linear relation was assumed. A weighted least square method was used. The confidence interval and prediction interval estimate for the slope was based on the t-distribution. A Wald-type p-value for the slope and the meta-regression coefficient of determination (R^{2*}) correlation coefficient was also given.

Forest plots were used to graphically summarize the results. For meta-regression, bubble plots were used. The square or dot sizes on the plots refer to the weight in the random effects meta-analysis. The t-distribution-based method is used for the CI of MedD calculation in individual studies.

Supplementary Results

Figs. S1-3 Analyses of the Shannon diversity index – Sensitivity analysis, subgroups by risk of bias and meta-regression

As a sensitivity analysis, we performed a separate calculation with more restricted inclusion criteria without studies performed with cross-over design [35, 36] providing change data only [37] or with not no clear number of participants [38] (Fig. S1) (MedD = –0.08 [–0.20 to 0.04]).

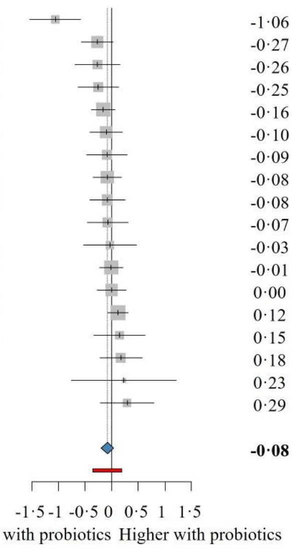

Study<br>Sensitivity analyis	Probiotics<br>Total	 Median	 Q1	 Q3	No Probiotics<br>Total	 Median	 Q1	 Q3	MedD	95%-CI	Weight
Shi et al. 2020	25	4·99	4·79	6·07	25	6·04	5·57	6·30	-1·06	[-1·53; -0·58]	3·3%
López-Garcia et al. 2023	20	2·83	2·71	3·12	19	3·10	2·72	3·21	-0·27	[-0·57; 0·03]	6·7%
Tremblay et al. 2021 25B	23	5·49	5·17	5·94	23	5·75	5·26	6·06	-0·26	[-0·69; 0·16]	4·1%
Freedman et al. 2021 *	23	4·97	4·45	5·49	16	5·22	4·92	5·52	-0·25	[-0·63; 0·13]	4·7%
Sánchez Macarro et al. 2021	22	2·90	2·77	3·24	21	3·06	2·79	3·15	-0·16	[-0·38; 0·07]	9·2%
Hibberd et al. 2018 *	24	6·90	6·48	7·32	36	7·00	6·64	7·36	-0·10	[-0·41; 0·21]	6·5%
Michael et al. 2020	35	6·51	6·22	7·06	29	6·60	6·22	7·02	-0·09	[-0·47; 0·29]	4·8%
Sohn et al. 2021	35	2·51	2·11	2·88	36	2·59	2·33	2·83	-0·08	[-0·34; 0·18]	7·8%
Son et al. 2020	8	2·31	2·10	2·48	7	2·39	2·22	2·54	-0·08	[-0·41; 0·25]	5·9%
Shi et al. 2023 *	25	3·22	2·70	3·74	25	3·29	2·89	3·69	-0·07	[-0·45; 0·31]	4·8%
Washburn et al. 2022	15	5·91	5·52	6·05	15	5·94	4·99	6·25	-0·03	[-0·53; 0·47]	3·1%
Sandiogini et al. 2022	25	3·97	3·75	4·13	25	3·98	3·82	4·31	-0·01	[-0·23; 0·22]	9·3%
Gai et al. 2023	48	4·13	3·63	4·36	46	4·13	3·63	4·36	0·00	[-0·27; 0·27]	7·4%
Kang et al. 2021	39	3·39	3·03	3·49	34	3·27	3·06	3·50	0·12	[-0·07; 0·31]	10·7%
Huang et al. 2022	12	6·03	5·88	6·42	12	5·88	5·69	6·48	0·15	[-0·33; 0·63]	3·3%
Park et al. 2020	31	4·11	3·67	4·44	33	3·94	3·39	4·37	0·18	[-0·22; 0·57]	4·5%
Majeed et al. 2023	12	4·73	4·15	6·26	14	4·50	4·18	5·22	0·23	[-0·75; 1·21]	0·9%
Chen et al. 2021	19	5·17	4·70	5·72	19	4·88	4·44	5·17	0·29	[-0·21; 0·80]	3·0%
**Random effect**									**-0·08**	**[-0·20; 0·04]**	**100·0%**
**Prediction interval**										**[-0·35; 0·19]**	
I^2= 42% [0%; 67%], τ = 0·11											

Fig. S1 Additional sensitivity analysis for the more restricted analysis of Shannon diversity index.

Abbreviations: CI: confidence interval; MedD: mean of median differences. Q1: first quartile; Q3: third quartile.

The “*” indicates that the median and q1, q3 are estimated from mean and standard deviation in that study.

Furthermore, we analysed our data as subgroups based on the results of risk of bias assessment (high, some concerns, or low risk of bias). These results did not reveal any significant or clinically relevant difference between the intervention and control groups either. High risk of bias: MedD = –0.18 [–0.60 to 0.24]; Some concerns: MedD = –0.09 [–0.20 to 0.02]; Low risk of bias: MedD = –0.01 [–0.14 to 0.11] (Fig. S2).

Study<br>Subgroup Risk of Bias	Total	Probiotics<br>Median	Q1	Q3	Total	No Probiotics<br>Median	Q1	Q3	MedD	95%-CI	Weight
**High**											
Shi et al. 2020	25	4·99	4·79	6·07	25	6·04	5·57	6·30	-1·06	[-1·53; -0·58]	2·0%
Moloney et al. 2021	16	5·70	5·48	5·92	24	5·82	5·66	6·10	-0·12	[-0·38; 0·14]	6·0%
Michael et al. 2020	35	6·51	6·22	7·06	29	6·60	6·22	7·02	-0·09	[-0·47; 0·29]	3·1%
Son et al. 2020	8	2·31	2·10	2·48	7	2·39	2·22	2·54	-0·08	[-0·41; 0·25]	4·0%
Gai et al. 2023	48	4·13	3·63	4·36	46	4·13	3·63	4·36	0·00	[-0·27; 0·27]	5·5%
Huang et al. 2022	12	6·03	5·88	6·42	12	5·88	5·69	6·48	0·15	[-0·33; 0·63]	2·0%
**Random effect**									**-0·18**	**[-0·60; 0·24]**	**22·7%**
I^2= 70% [30%; 87%] ,τ= 0·32											
**Low**											
Freedman et al. 2021 *	23	4·97	4·45	5·49	16	5·22	4·92	5·52	-0·25	[-0·63; 0·13]	3·1%
Sánchez Macarro et al. 2021	22	2·90	2·77	3·24	21	3·06	2·79	3·15	-0·16	[-0·38; 0·07]	7·5%
Hibberd et al. 2018 *	24	6·90	6·48	7·32	36	7·00	6·64	7·36	-0·10	[-0·41; 0·21]	4·6%
Shi et al. 2023 *	25	3·22	2·70	3·74	25	3·29	2·89	3·69	-0·07	[-0·45; 0·31]	3·1%
Washburn et al. 2022	15	5·91	5·52	6·05	15	5·94	4·99	6·25	-0·03	[-0·53; 0·47]	1·9%
Kang et al. 2021	39	3·39	3·03	3·49	34	3·27	3·06	3·50	0·12	[-0·07; 0·31]	9·6%
Nakamura et al. 2022	20	6·27	5·57	6·51	20	6·12	5·86	6·44	0·15	[-0·26; 0·56]	2·7%
Majeed et al. 2023	12	4·73	4·15	6·26	14	4·50	4·18	5·22	0·23	[-0·75; 1·21]	0·5%
Chen et al. 2021	19	5·17	4·70	5·72	19	4·88	4·44	5·17	0·29	[-0·21; 0·80]	1·8%
**Random effect**									**-0·01**	**[-0·14; 0·11]**	**34·8%**
**Prediction interval**										**[-0·23; 0·20]**	
I^2= 0% [0%; 65%] ,τ = 0·07											
**Some concerns**											
López-Garcia et al. 2023	20	2·83	2·71	3·12	19	3·10	2·72	3·21	-0·27	[-0·53; -0·01]	6·0%
Tremblay et al. 2021 25B	23	5·49	5·17	5·94	23	5·75	5·26	6·06	-0·26	[-0·69; 0·16]	2·6%
Bloemendaal et al. 2021	27	-0·12	-0·38	0·20	31	0·04	-0·30	0·20	-0·16	[-0·42; 0·10]	6·1%
Sohn et al. 2021	35	2·51	2·11	2·88	36	2·59	2·33	2·83	-0·08	[-0·34; 0·18]	5·9%
Moore et al. 2023	48	2·94	2·86	3·25	42	3·00	2·75	3·25	-0·06	[-0·23; 0·11]	11·3%
Sandiogini et al. 2022	25	3·97	3·75	4·13	25	3·98	3·82	4·31	-0·01	[-0·23; 0·22]	7·6%
Park et al. 2020	31	4·11	3·67	4·44	33	3·94	3·39	4·37	0·18	[-0·22; 0·57]	2·9%
**Random effect**									**-0·09**	**[-0·20; 0·02]**	**42·5%**
I^2= 0% [0%; 71%] ,τ = < 0·01											
**Random effect**									**-0·08**	**[-0·16; 0·01]**	**100·0%**
**Prediction interval**										**[-0·24; 0·08]**	
I^2= 34% [0%; 60%] ,τ = 0·06											
Test for subgroup differences: p = 0·43											

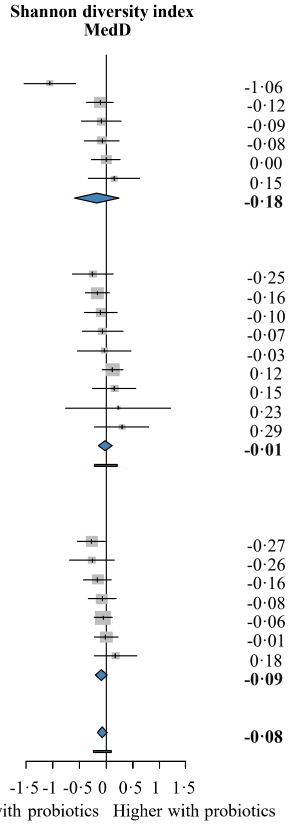

Fig. S2 Additional sensitivity analysis for the subgroups based on risk of bias assessment for Shannon diversity index.

Abbreviations: CI: confidence interval; MedD: mean of median differences. Q1: first quartile; Q3: third quartile.

The “*” indicates that the median and q1, q3 are estimated from mean and standard deviation in that study.

Using the mixed-effects meta-regression model, we tested whether the duration of probiotic intervention significantly affected gut microbiota diversity compared to the control group. Assuming a linear relation, the estimated slope is 0, 95% CI [–0.01 to 0.02] [MedD value / weeks] (p-value: 0.5961). The R^{2*} value is 0%. The results indicated no significant association between intervention time and diversity outcomes, suggesting that the length of probiotic use did not meaningfully influence the observed effects (Fig. S3).

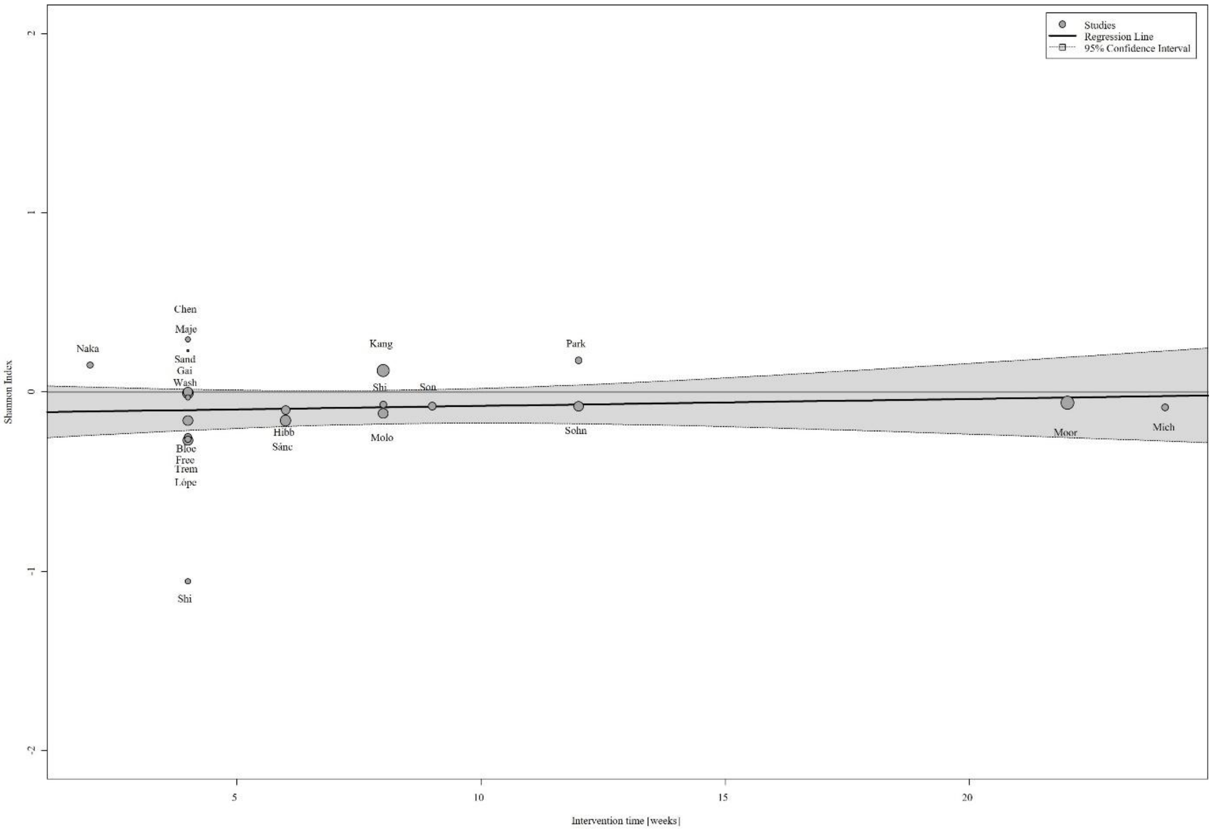

Fig. S3 Meta-regression analysis investigating the relationship between intervention duration and the Shannon diversity index.

Figs. S4-6 Analyses of the Observed OTUs diversity index – Sensitivity analysis, subgroups by risk of bias and meta-regression

We did not identify significant difference when removing studies with not clear data on the number of participants [38], cross-over design [35] and change results [37] (Fig. S4).

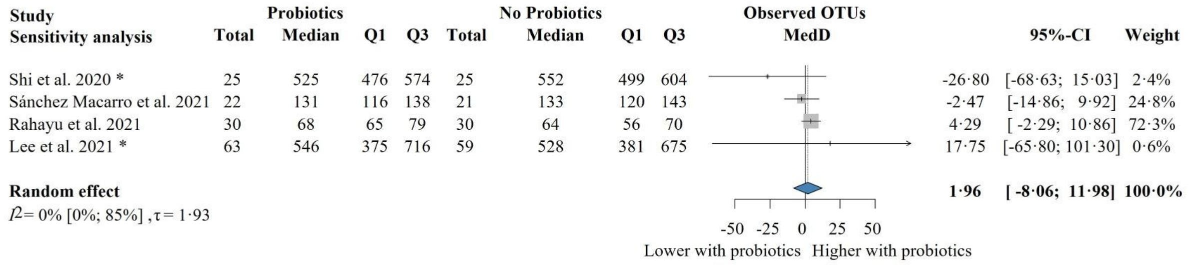

Fig. S4 Additional sensitivity analysis for the more restricted analysis of Observed OTUs.

Abbreviations: OTU: Operational Taxonomic Unit; CI: confidence interval; MedD: mean of median differences. Q1: first quartile; Q3: third quartile.

The “*” indicates that the median and q1, q3 are estimated from mean and standard deviation in that study.

Subgroup analysis based on the risk of bias assessment led to similar insignificant findings (Fig. S5).

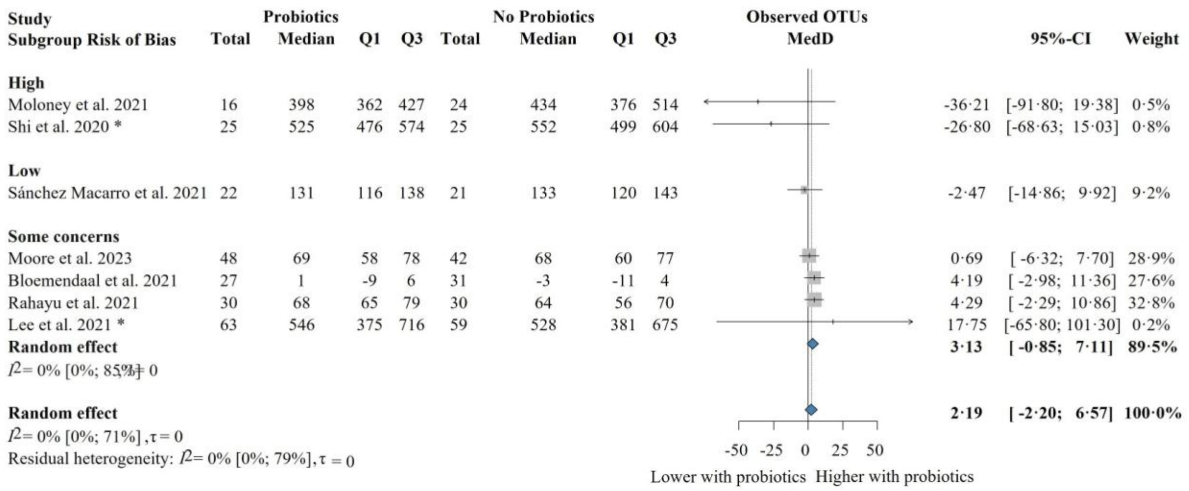

Fig. S5 Additional sensitivity analysis for the subgroups based on risk of bias assessment for Observed OTUs.

Abbreviations: CI: confidence interval; MedD: mean of median differences. Q1: first quartile; Q3: third quartile.

The "*" indicates that the median and q1, q3 are estimated from mean and standard deviation in that study.

Using the mixed-effects meta-regression model, we tested whether the duration of probiotic intervention significantly affected gut microbiota diversity compared to the control group. Assuming a linear relation, the estimated slope is –0.06, 95% CI [–0.77 to 0.65] [MedD value / weeks] (p-value: 0.8367). The R^{2*} value is 0%. The results indicated no significant association between intervention time and diversity outcomes, suggesting that the length of probiotic use did not meaningfully influence the observed effects (Fig. S6).

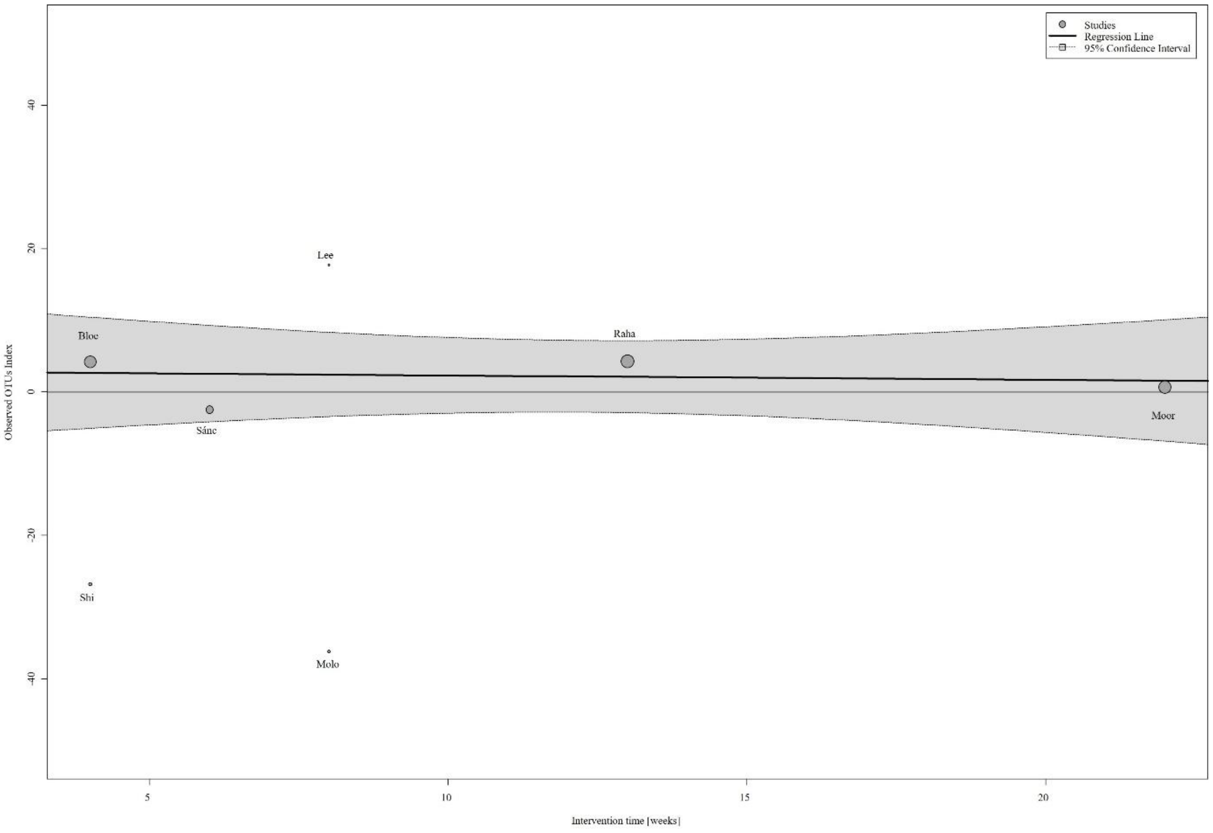

Fig. S6 Meta-regression analysis investigating the relationship between intervention duration and the number of Observed OTUs.

Figs. S7-9 Analyses of the Chao1 index – Sensitivity analysis, subgroups by risk of bias and meta-regression

The more restricted sensitivity analysis removing studies with cross-over design [35, 39] revealed no significant or relevant difference between groups either (Fig. S7) (MedD = 1.02 [–25.00 to 27.03]).

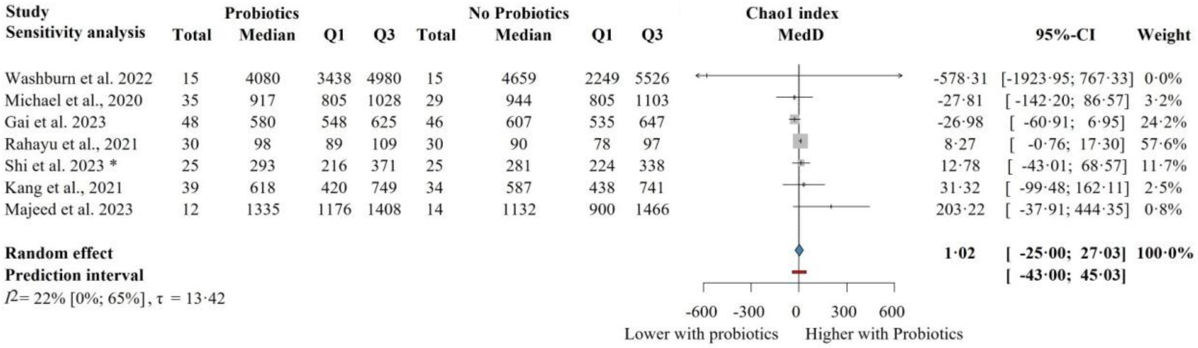

Fig. S7 Additional sensitivity analysis for the more restricted analysis Chao1 index.

Abbreviations: CI: confidence interval; MedD: mean of median differences. Q1: first quartile; Q3: third quartile.

The "*" indicates that the median and q1, q3 are estimated from mean and standard deviation in that study.

The results of the additional subgroup analyses based on the risk of bias assessment are shown in Fig. S8, with no significant differences between the groups.

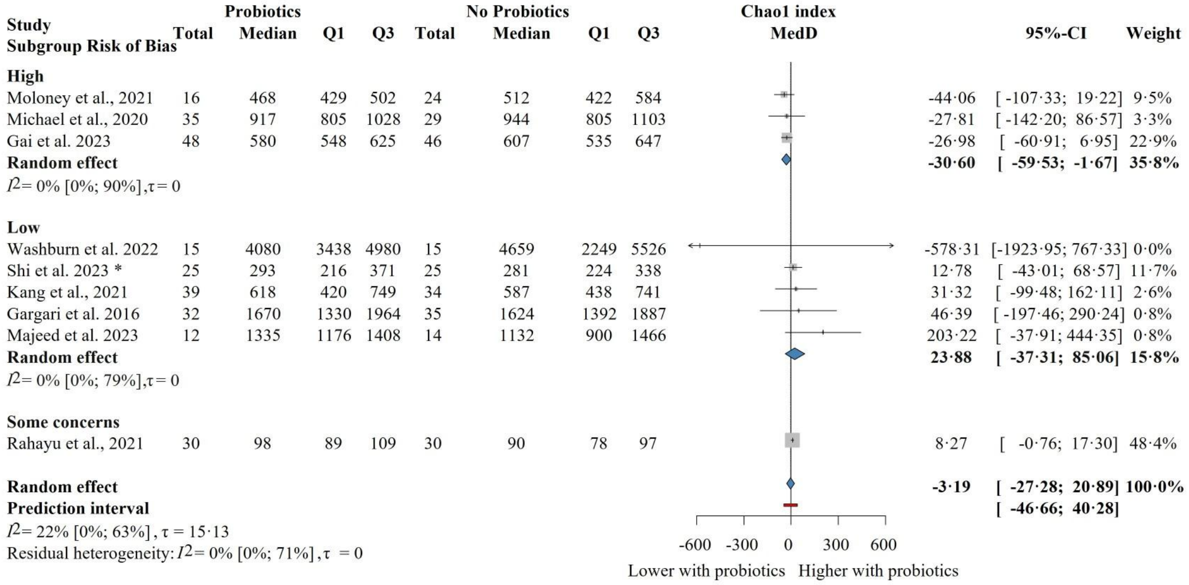

Fig. S8 Additional sensitivity analysis for the subgroups based on the risk of bias assessment Chao1 index.

Abbreviations: CI: confidence interval; MedD: mean of median differences. Q1: first quartile; Q3: third quartile.

The "*" indicates that the median and q1, q3 are estimated from mean and standard deviation in that study.

Using the mixed-effects meta-regression model, we tested whether the duration of probiotic intervention significantly affected gut microbiota diversity compared to the control group. Assuming a linear relation, the estimated slope 1.75, 95% CI [–4.01 to 7.51] [MD value / weeks] (p-value: 0.4957). The R^{2*} value is 41.3%. The results indicated no significant association between intervention time and diversity outcomes, suggesting that the length of probiotic use did not meaningfully influence the observed effects (Fig. S9).

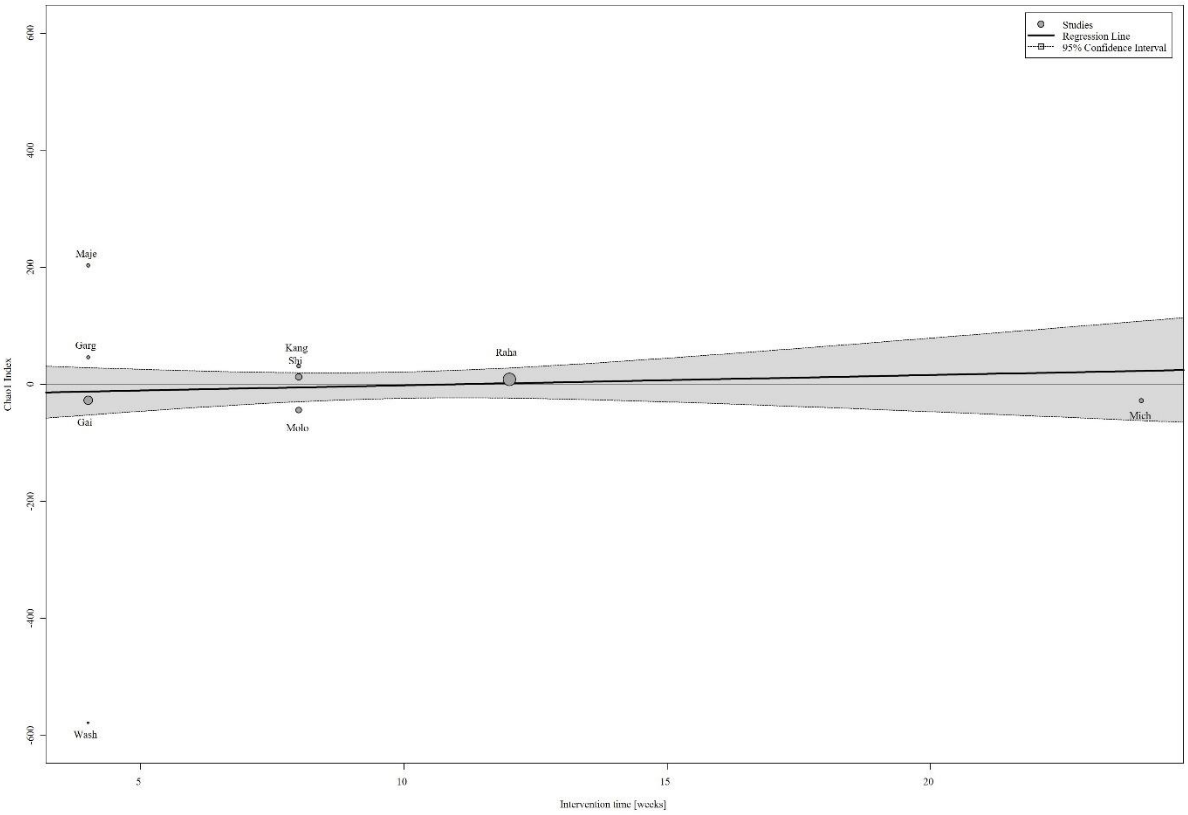

Fig. S9 Meta-regression analysis investigating the relationship between intervention duration and the Chao1 index.

Figs. S10-12 Analyses of the Simpson's Index of Diversity - Sensitivity analysis, subgroups by risk of bias and meta-regression

In the sensitivity analysis we removed the study with cross-over design [35] and the one with no clear number of participants [38], but we did not reveal any effect of probiotics (Fig. S10).

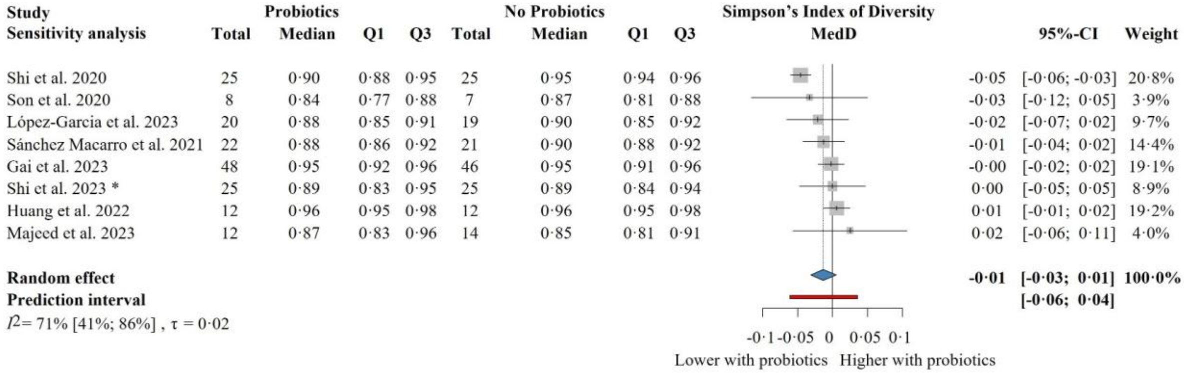

Fig. S10 Additional sensitivity analysis for the more restricted analysis of Simpson's Index of Diversity.

Abbreviations: CI: confidence interval; MedD: mean of median differences. Q1: first quartile; Q3: third quartile.

The "*" indicates that the median and q1, q3 are estimated from mean and standard deviation in that study.

The subgroup analyses based on the risk of bias assessment did not reveal significant differences between probiotic and control groups (Fig. S11).

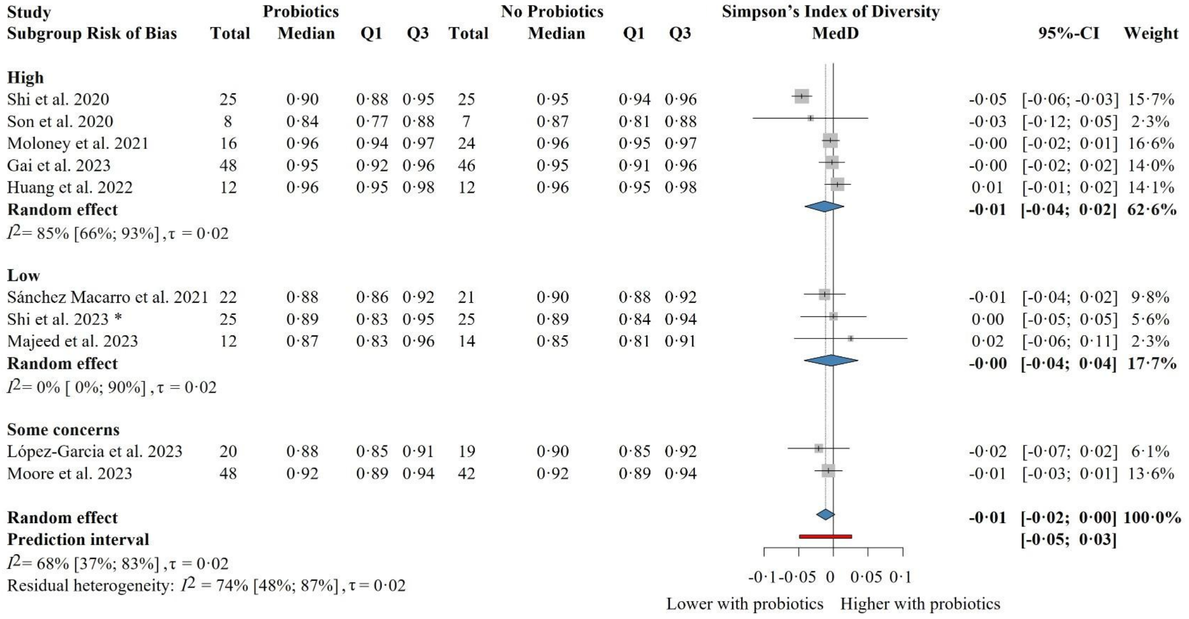

Fig. S11 Additional sensitivity analysis for the subgroups based on risk of bias assessment of Simpson's Index of Diversity.

Abbreviations: CI: confidence interval; MedD: mean of median differences. Q1: first quartile; Q3: third quartile.

The "*" indicates that the median and q1, q3 are estimated from mean and standard deviation in that study.

Using the mixed-effects meta-regression model, we tested whether the duration of probiotic intervention significantly affected gut microbiota diversity compared to the control group. Assuming a linear relation, the estimated slope is 0, 95% CI [0 to 0] [MedD value / weeks] (p-value: 0.513). The R^{2*} value is 0%. The results indicated no significant association between intervention time and diversity outcomes, suggesting that the length of probiotic use did not meaningfully influence the observed effects (Fig. S12).

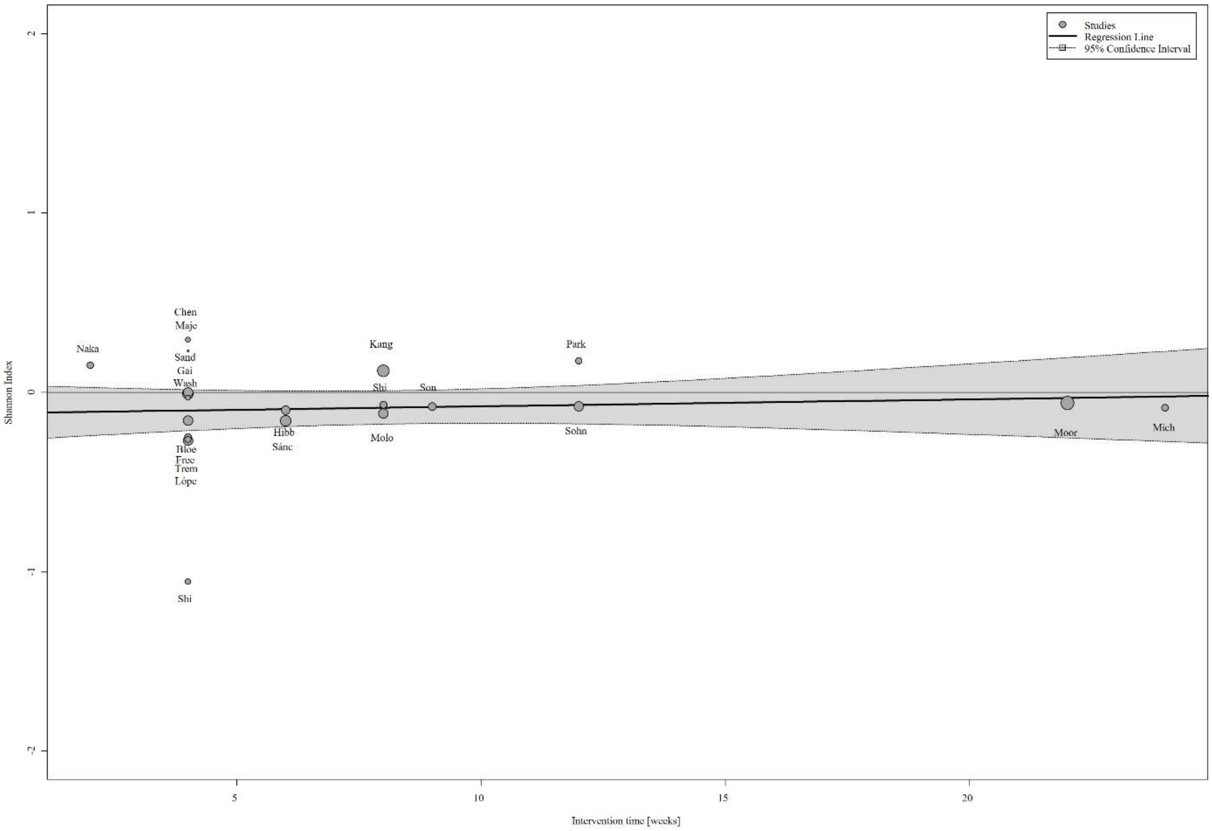

Fig. S12 Meta-regression analysis investigating the relationship between intervention duration and the Simpson’s Index of Diversity

Figs. S13-20 Publication bias and leave-out analyses

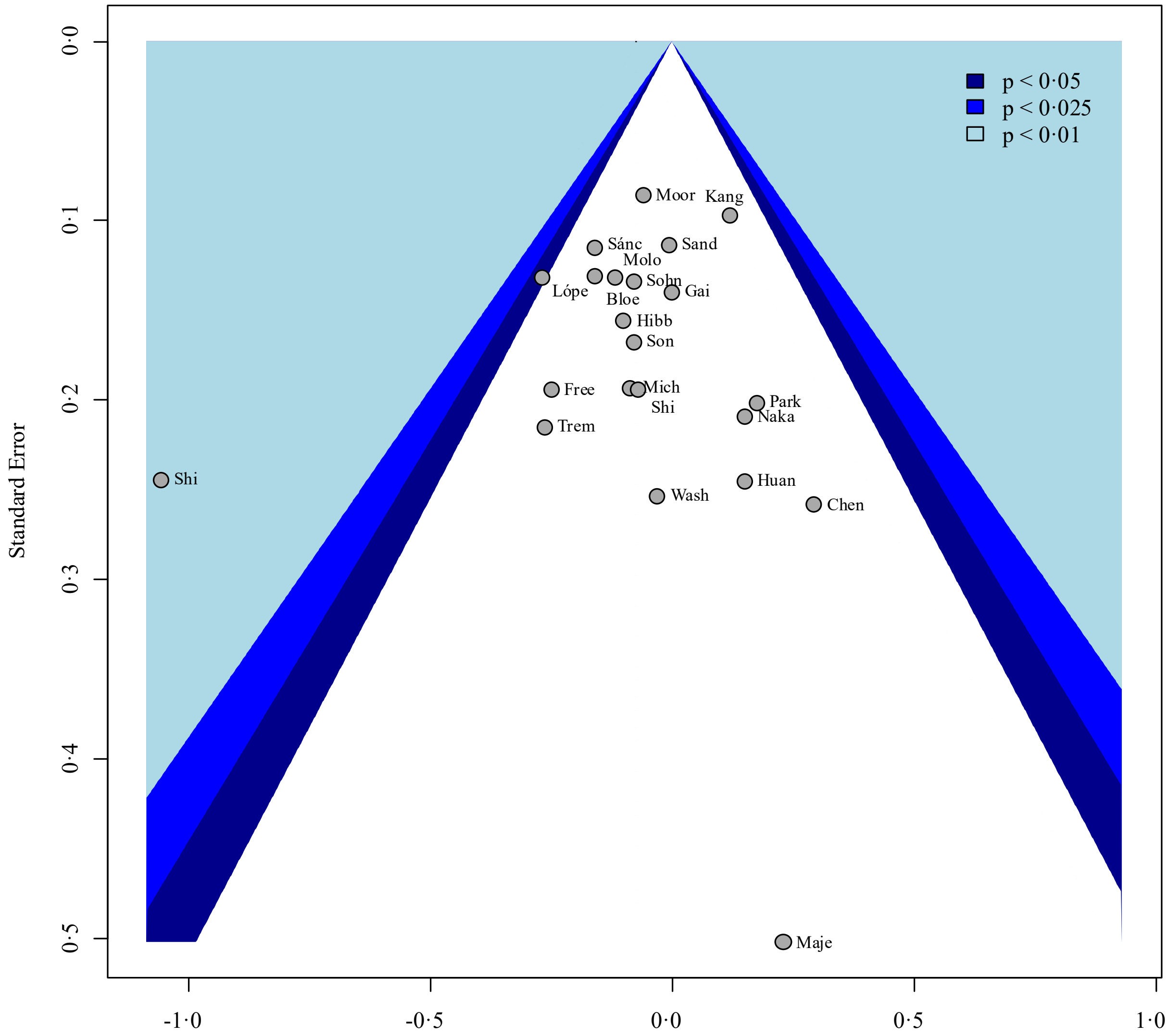

Fig. S13 Funnel plot to assess publication bias for Shannon diversity index.

The points on the funnel plots for assessing publication bias represent the different studies. It shows the residuals on the x-axis against their corresponding standard errors. Here we could assess the small study bias: at the bottom of the funnel if the studies are distributed not symmetrically and out of the funnel, it indicates potential publication bias. Egger's test p-value is 0.6887.

In the leave-one-out analysis, the Kang et al. 2021 [40] study can be considered a statistically influential study. However, the analysis indicates that it does not clinically relevantly affect the point estimate of the effect size or its confidence interval (Fig. S14).

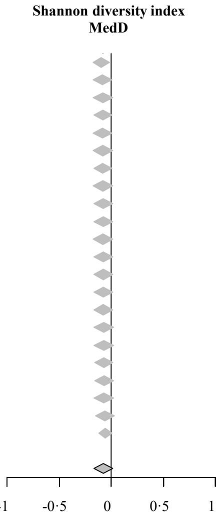

Study	Influential	effect size	95% CI	I2	Std residual	Dffits	Cook's dist·	Covariance ratio	Hat value
Omitting Kang et al. 2021	*	-0·10	[-0·18; -0·01]	0·27	2·09	0·66	0·30	0·89	0·10
Omitting Park et al. 2020		-0·08	[-0·17; 0·00]	0·34	1·21	0·21	0·04	1·03	0·03
Omitting Sandiogini et al. 2022		-0·08	[-0·18; 0·01]	0·36	0·53	0·18	0·04	1·19	0·08
Omitting Chen et al. 2021		-0·08	[-0·17; 0·00]	0·32	1·40	0·19	0·03	1·01	0·02
Omitting Nakamura et al. 2022		-0·08	[-0·17; 0·01]	0·34	1·05	0·18	0·03	1·04	0·03
Omitting Gai et al. 2023		-0·08	[-0·17; 0·01]	0·36	0·50	0·15	0·02	1·14	0·05
Omitting Huang et al. 2022		-0·08	[-0·17; 0·01]	0·35	0·90	0·14	0·02	1·04	0·02
Omitting Moore et al. 2023		-0·08	[-0·18; 0·02]	0·37	0·15	0·09	0·01	1·30	0·11
Omitting Majeed et al. 2023		-0·08	[-0·17; 0·01]	0·36	0·61	0·05	0·00	1·01	0·01
Omitting Washburn et al. 2022		-0·08	[-0·17; 0·01]	0·37	0·18	0·04	0·00	1·05	0·02
Omitting Sohn et al. 2021		-0·08	[-0·17; 0·02]	0·37	-0·01	0·03	0·00	1·17	0·06
Omitting Shi et al. 2023 *		-0·08	[-0·17; 0·01]	0·37	0·03	0·02	0·00	1·09	0·03
Omitting Son et al. 2020		-0·08	[-0·17; 0·02]	0·37	-0·01	0·02	0·00	1·11	0·04
Omitting Michael et al. 2020		-0·08	[-0·17; 0·01]	0·37	-0·05	0·01	0·00	1·09	0·03
Omitting Hibberd et al. 2018 *		-0·08	[-0·17; 0·02]	0·37	-0·13	-0·00	0·00	1·13	0·05
Omitting Moloney et al. 2021		-0·07	[-0·17; 0·02]	0·37	-0·28	-0·04	0·00	1·16	0·06
Omitting Bloemendaal et al. 2021		-0·07	[-0·16; 0·02]	0·36	-0·57	-0·13	0·02	1·13	0·06
Omitting Tremblay et al. 2021 25B		-0·07	[-0·16; 0·02]	0·35	-0·85	-0·14	0·02	1·03	0·03
Omitting Freedman et al. 2021 *		-0·07	[-0·16; 0·02]	0·35	-0·86	-0·15	0·02	1·04	0·03
Omitting Sánchez Macarro et al. 2021		-0·07	[-0·16; 0·02]	0·36	-0·63	-0·16	0·03	1·14	0·07
Omitting López-Garcia et al. 2023		-0·06	[-0·15; 0·02]	0·32	-1·46	-0·42	0·16	0·94	0·06
Omitting Shi et al. 2020		-0·06	[-0·12; 0·01]	0·00	-4·06	-0·60	0·34	0·81	0·02
**Random effect** I^2 = 0% [0%; 46%]		**-0·08**	**[-0·16; 0·01]**						

Fig. S14 Additional leave-one-out analysis for Shannon diversity index.

Cases which are considered as possible influential with respect to any of the shown measures are marked with "*" at column "*Influential*". Note that the chosen cut-offs are (somewhat) arbitrary based on *dmetar* package. "*Effect size*": the pooled effect size without the given study. "*95% CI*": the 95% confidence interval of the pooled effect size without the given study. "I^2": the Higgins&Thomson I^2 heterogeneity value without the given study. "*Std residual*": the studentized residuals. It shows the deleted residual divided by its estimated standard deviation. "*Dffits*": the difference in fits. It quantifies the number of standard deviations that the fitted value changes without the given study. (Typical threshold is $3 * \sqrt{(p/(k-p))}$, where p is the number of model coefficients and k is the number of cases). "*Cook's dist.*": Cook's distance. It depends on both the residual and leverage of the omitted study. (Typical threshold value is 2). "*Covariance ratio*": the covariance ratio. It shows the change in the determinant of the covariance matrix of the effect size. (Typical threshold value is 1). "*Hat value*": the value of the hat matrix without the given study. (Typical threshold is $3*p/k$)

Abbreviations: MedD: mean of median differences; CI: confidence interval.

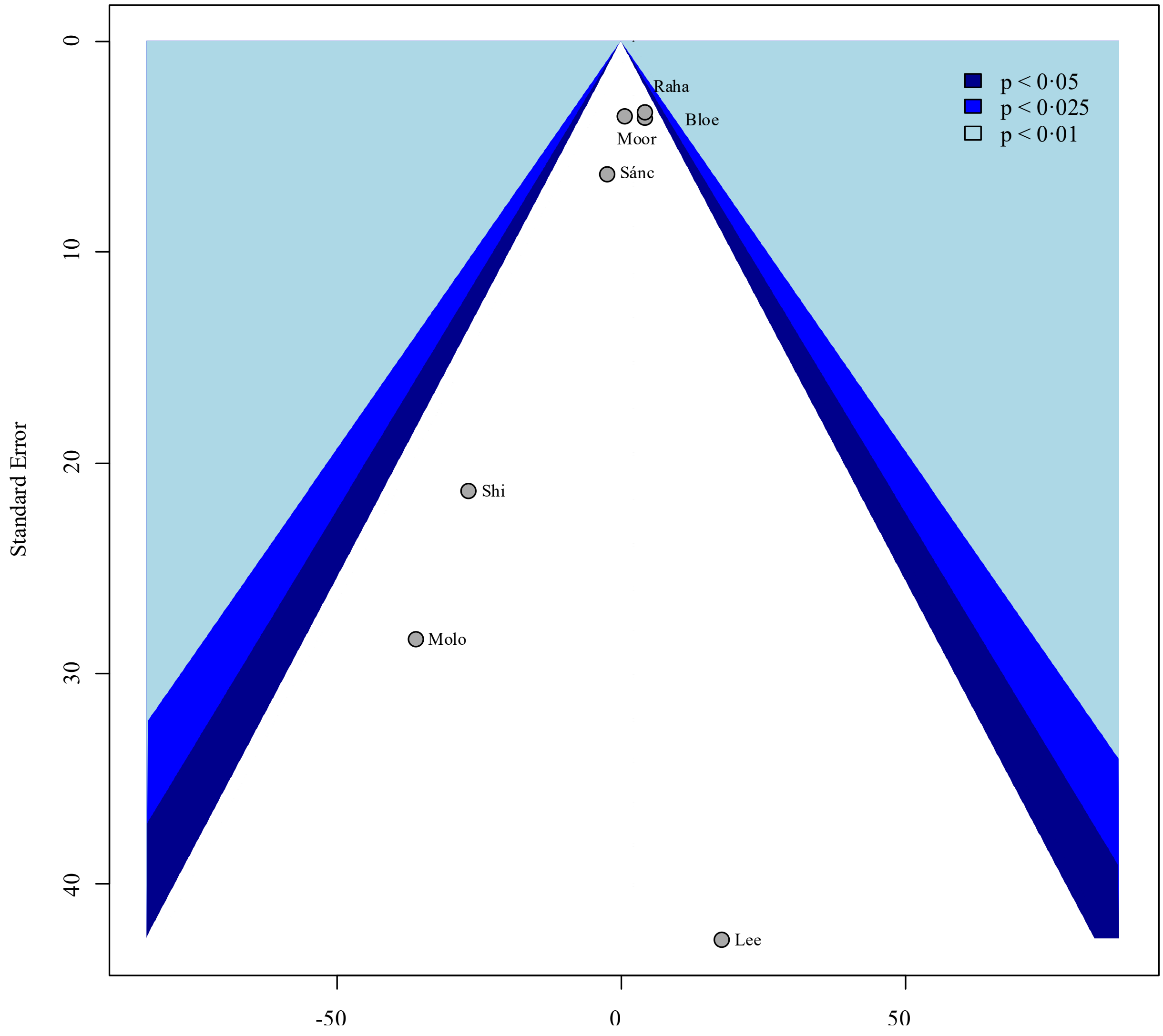

Fig. S15 Funnel plot to assess publication bias for the Observed Operational Taxonomic Units (OTUs).

The points on the funnel plots for assessing publication bias represent the different studies. It shows the residuals on the x-axis against their corresponding standard errors. Here we could assess the small study bias: at the bottom of the funnel if the studies are distributed not symmetrically and out of the funnel, it indicates potential publication bias. Egger's test p-value is 0.1093.

In the leave-one-out analysis, the Shi et al. 2023 [41], Moloney et al. 2021 [35], and Gai et al. 2023 [42] studies can be considered a statistically influential study. However, the analysis indicates that they do not clinically relevantly affect the point estimate of the effect size or its confidence interval (Fig. S16).

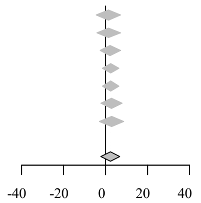

Study	Observed OTUs MedD	Influential	effect size	95% CI	I2	Std residual	Dffits	Cook's dist·	Covariance ratio	Hat value
Omitting Rahayu et al. 2021			1·16	[-4·64; 6·97]	0·00	0·76	0·53	0·28	1·49	0·33
Omitting Bloemendaal et al. 2021			1·42	[-4·27; 7·12]	0·00	0·64	0·40	0·16	1·38	0·28
Omitting Lee et al. 2021 *			2·16	[-2·83; 7·14]	0·02	0·37	0·02	0·00	1·00	0·00
Omitting Moloney et al. 2021			2·37	[-1·71; 6·44]	0·00	-1·36	-0·09	0·01	1·00	0·00
Omitting Shi et al. 2020 *			2·43	[-1·64; 6·49]	0·00	-1·36	-0·12	0·02	1·01	0·01
Omitting Sánchez Macarro et al. 2021			2·66	[-2·32; 7·65]	0·00	-0·77	-0·25	0·06	1·10	0·09
Omitting Moore et al. 2023			2·80	[-3·05; 8·64]	0·00	-0·50	-0·32	0·10	1·41	0·29
**Random effect**			**2·19**	**[-2·20; 6·57]**						
I^2 = 0% [0%; 71%]										

Fig. S16 Additional leave-one-out analysis for the Observed Operational Taxonomic Units (OTUs).

Cases which are considered as possible influential with respect to any of the shown measures are marked with "*" at column "*Influential*". Note that the chosen cut-offs are (somewhat) arbitrary based on *dmetar* package. "*Effect size*": the pooled effect size without the given study. "*95% CI*": the 95% confidence interval of the pooled effect size without the given study. "I^2": the Higgins&Thomson I^2 heterogeneity value without the given study. "*Std residual*": the studentized residuals. It shows the deleted residual divided by its estimated standard deviation. "*Dffits*": the difference in fits. It quantifies the number of standard deviations that the fitted value changes without the given study. (Typical threshold is $3* \sqrt{(p/(k-p))}$, where p is the number of model coefficients and k is the number of cases). "*Cook's dist.*": Cook's distance. It depends on both the residual and leverage of the omitted study. (Typical threshold value is 2). "*Covariance ratio*": the covariance ratio. It shows the change in the determinant of the covariance matrix of the effect size. (Typical threshold value is 1). "*Hat value*": the value of the hat matrix without the given study. (Typical threshold is $3*p/k$)

Abbreviations: MedD: mean of median differences; CI: confidence interval.

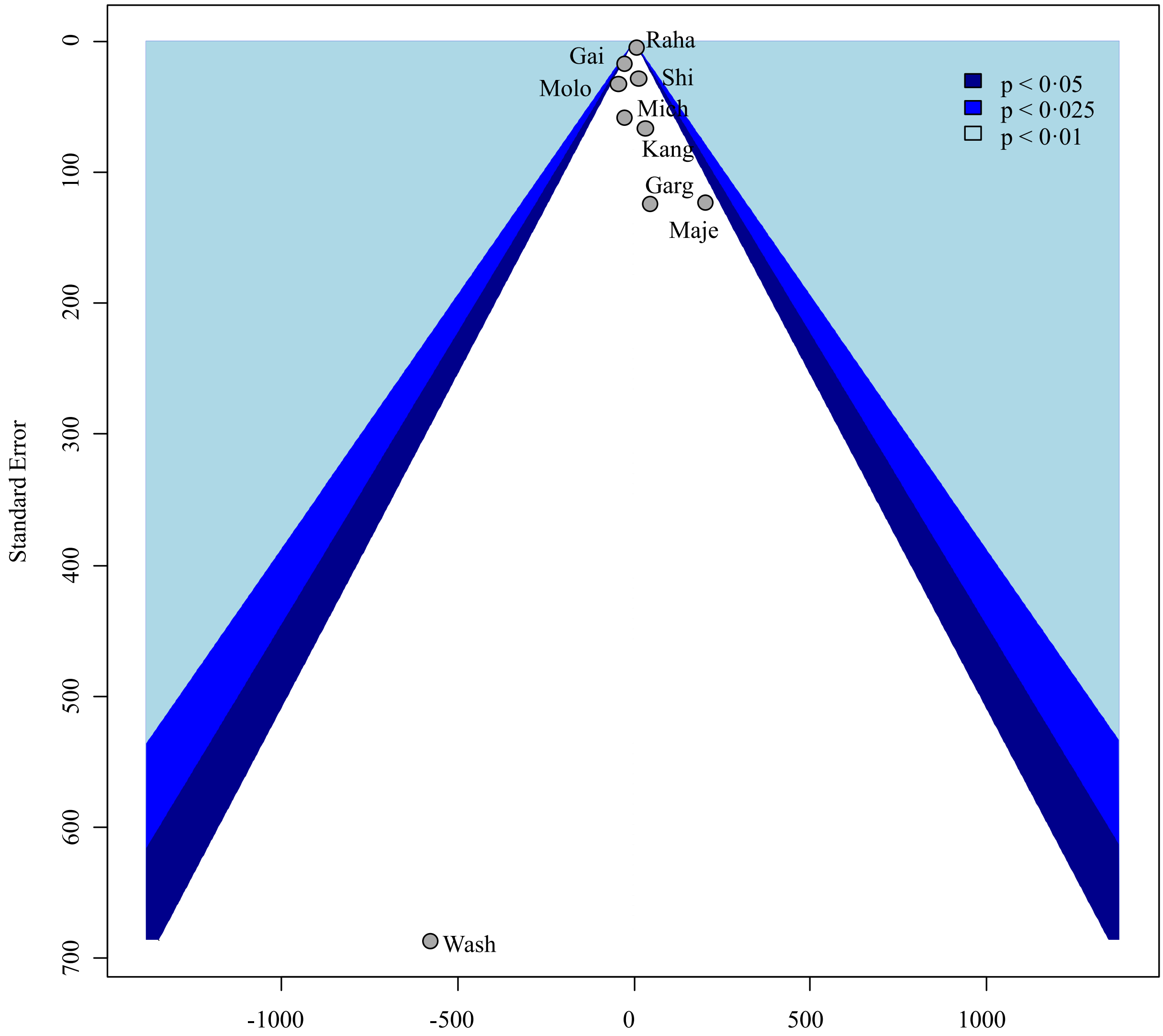

Fig. S17 Funnel plot to assess publication bias for Chao1 index.

The points on the funnel plots for assessing publication bias represent the different studies. It shows the residuals on the x-axis against their corresponding standard errors. Here we could assess the small study bias: at the bottom of the funnel if the studies are distributed not symmetrically and out of the funnel, it indicates potential publication bias. Egger's test p-value is 0.5996.

In the leave-one-out analysis, the Shi et al. 2023 [41], Moloney et al. 2021 [35], and Gai et al. 2023 [42] studies can be considered a statistically influential study. However, the analysis indicates that they do not clinically relevantly affect the point estimate of the effect size or its confidence interval (Fig. S18).

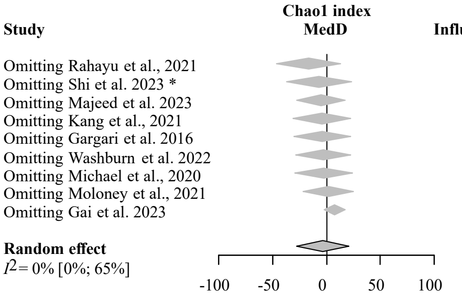

Study	Influential	effect size	95% CI	I2	Std residual	Dffits	Cook's dist·	Covariance ratio	Hat value
Omitting Rahayu et al., 2021	*	-16·58	[-46·26; 13·11]	0·00	1·84	4·17	1·48	1·34	0·48
Omitting Shi et al. 2023 *		-6·54	[-36·55; 23·46]	0·31	0·53	0·29	0·09	1·45	0·12
Omitting Majeed et al. 2023		-5·06	[-27·15; 17·03]	0·08	1·67	0·17	0·03	1·05	0·01
Omitting Kang et al., 2021		-4·46	[-31·37; 22·46]	0·30	0·51	0·11	0·01	1·10	0·03
Omitting Gargari et al. 2016		-3·69	[-30·14; 22·75]	0·31	0·40	0·05	0·00	1·03	0·01
Omitting Washburn et al. 2022		-3·03	[-28·08; 22·02]	0·26	-0·84	-0·02	0·00	1·00	0·00
Omitting Michael et al., 2020		-2·46	[-29·32; 24·40]	0·29	-0·41	-0·07	0·00	1·07	0·03
Omitting Moloney et al., 2021		1·41	[-21·77; 24·60]	0·10	-1·25	-0·43	0·18	0·92	0·10
Omitting Gai et al. 2023	*	7·55	[-2·62; 17·72]	0·00	-1·93	-1·30	0·95	0·17	0·23
**Random effect** I^2= 0% [0%; 65%]		**-3.19**	**[-27·28; 20·89]**						

Fig. S18 Additional leave-one-out analysis for Chao1 index.

Cases which are considered as possible influential with respect to any of the shown measures are marked with "*" at column "*Influential*". Note that the chosen cut-offs are (somewhat) arbitrary based on *dmetar* package. "*Effect size*": the pooled effect size without the given study. "*95% CI*": the 95% confidence interval of the pooled effect size without the given study. "I^2": the Higgins&Thomson I^2 heterogeneity value without the given study. "*Std residual*": the studentized residuals. It shows the deleted residual divided by its estimated standard deviation. "*Dffits*": the difference in fits. It quantifies the number of standard deviations that the fitted value changes without the given study. (Typical threshold is $3 * \sqrt{(p/(k-p))}$, where *p* is the number of model coefficients and *k* is the number of cases). "*Cook's dist.*": Cook's distance. It depends on both the residual and leverage of the omitted study. (Typical threshold value is 2). "*Covariance ratio*": the covariance ratio. It shows the change in the determinant of the covariance matrix of the effect size. (Typical threshold value is 1). "*Hat value*": the value of the hat matrix without the given study. (Typical threshold is $3*p/k$)

Abbreviations: MedD: mean of median differences; CI: confidence interval.

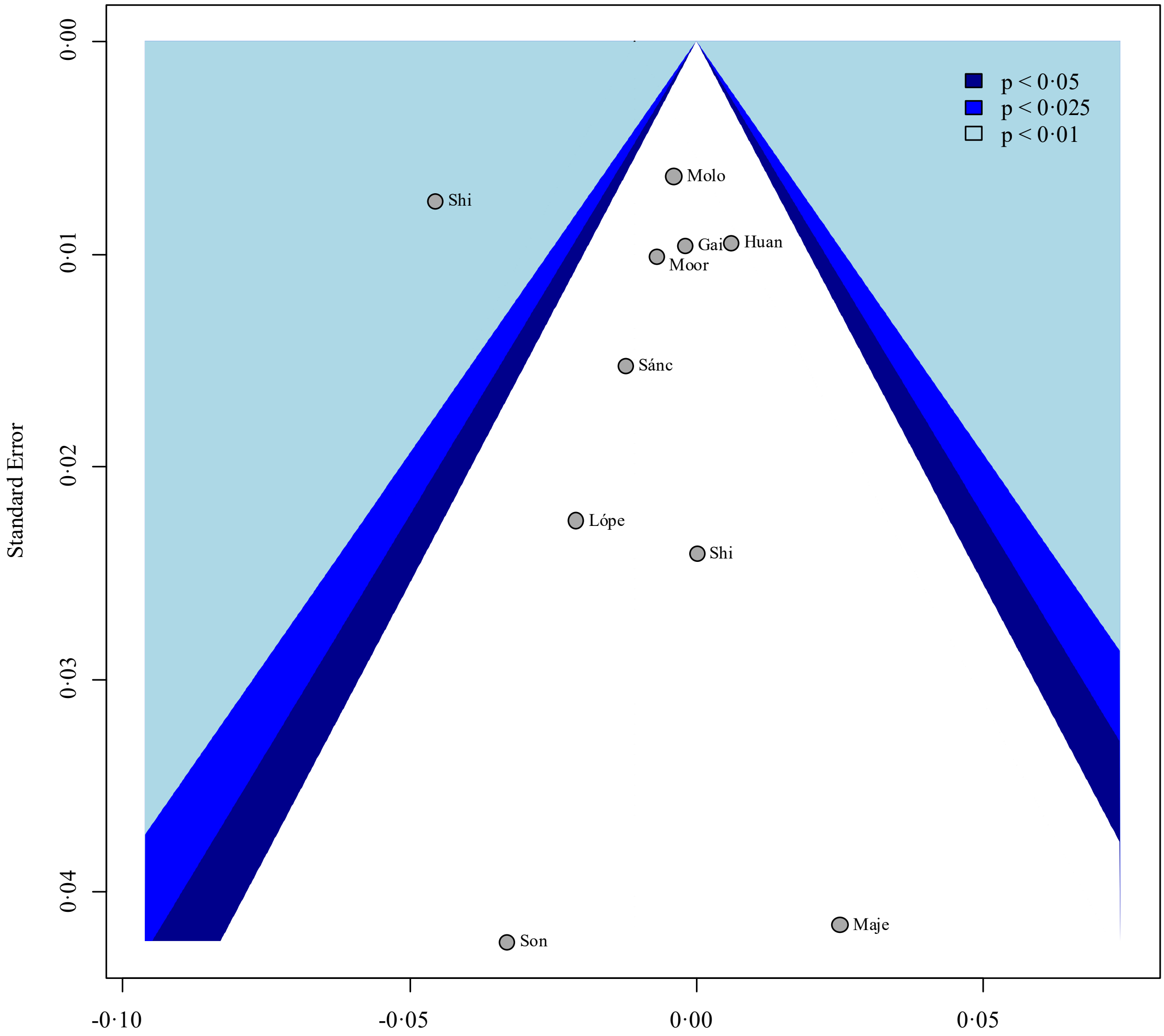

Fig. S19 Funnel plot to assess publication bias for Simpson's Index of Diversity.

The points on the funnel plots for assessing publication bias represent the different studies. It shows the residuals on the x-axis against their corresponding standard errors. Here we could assess the small study bias: at the bottom of the funnel if the studies are distributed not symmetrically and out of the funnel, it indicates potential publication bias. Egger's test p-value is 0.7448.

In the leave-one-out analysis the Shi et al. 2023 [43] study can be considered a statistically influential study based on the I² value and the covariance ratio. However, the analysis indicates that it does not clinically relevantly affect the point estimate of the effect size or its confidence interval (Fig. S20).

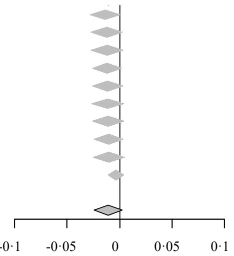

Study	Simpson's Index of Diversity MedD	Influential	effect size	95% CI	I2	Std residual	Dffits	Cook's dist·	Covariance ratio	Hat value
Omitting Huang et al. 2022			-0·01	[-0·03; 0·00]	0·66	1·01	0·41	0·17	1·15	0·14
Omitting Gai et al. 2023			-0·01	[-0·03; 0·00]	0·70	0·50	0·19	0·04	1·26	0·14
Omitting Moloney et al. 2021			-0·01	[-0·03; 0·00]	0·68	0·42	0·17	0·03	1·33	0·17
Omitting Majeed et al. 2023			-0·01	[-0·03; 0·00]	0·70	0·82	0·13	0·02	1·03	0·02
Omitting Shi et al. 2023 *			-0·01	[-0·03; 0·00]	0·71	0·39	0·09	0·01	1·12	0·06
Omitting Moore et al. 2023			-0·01	[-0·03; 0·00]	0·71	0·21	0·07	0·01	1·29	0·14
Omitting Sánchez Macarro et al. 2021			-0·01	[-0·03; 0·00]	0·71	-0·08	-0·04	0·00	1·23	0·10
Omitting Son et al. 2020			-0·01	[-0·02; 0·00]	0·71	-0·50	-0·08	0·01	1·05	0·02
Omitting López-Garcia et al. 2023			-0·01	[-0·02; 0·00]	0·71	-0·38	-0·11	0·01	1·14	0·06
Omitting Shi et al. 2020		*	-0·00	[-0·01; 0·00]	0·00	-4·98	-2·52	1·23	0·33	0·16
**Random effect** I^2 = 0% [0%; 62%]			**-0·01**	**[-0·02; 0·00]**						

Fig. S20 Additional leave-one-out analysis Simpson's Index of Diversity.

Cases which are considered as possible influential with respect to any of the shown measures are marked with "*" at column "*Influential*". Note that the chosen cut-offs are (somewhat) arbitrary based on *dmetar* package. "*Effect size*": the pooled effect size without the given study. "*95% CI*": the 95% confidence interval of the pooled effect size without the given study. "I^2": the Higgins&Thomson I^2 heterogeneity value without the given study. "*Std residual*": the studentized residuals. It shows the deleted residual divided by its estimated standard deviation. "*Dffits*": the difference in fits. It quantifies the number of standard deviations that the fitted value changes without the given study. (Typical threshold is $3* \sqrt{(p/(k-p))}$, where p is the number of model coefficients and k is the number of cases). "*Cook's dist.*": Cook's distance. It depends on both the residual and leverage of the omitted study. (Typical threshold value is 2). "*Covariance ratio*": the covariance ratio. It shows the change in the determinant of the covariance matrix of the effect size. (Typical threshold value is 1). "*Hat value*": the value of the hat matrix without the given study. (Typical threshold is $3*p/k$)

Abbreviations: MedD: mean of median differences; CI: confidence interval.

Table S4 Changes in the microbiome α-diversity indices as measured after the intervention period.

Study	Shannon diversity index	Chao1 index	Observed OTUs / Richness	Pielou's evenness	Simpson's Index of Diversity	Inverse Simpson index	Strong's dominance index	Sobs index	ACE index	Faiths Phylogenetic Diversity	Shannon effective count	Not specified
Axelrod (2019)	■	■			■				■			
Bagga (2018)						■						
Bazanella (2017)			■								■	
Bloemendaal (2021)	■		■							■		
Boesmans (2018)		■		■								
Castanet (2020)												■
Chen (2021)	■											
Chen (2023)	■											

De Andrés (2018) I. *B. infantis* R0033												
De Andrés (2018) II. *L. helveticus* R0052												
De Andrés (2018) III. *B. bifidum* R0071												
Ferrario (2014)												
Freedman (2021)												
Gai (2023)												
Gan (2022) [a]												
Gargari (2016)												
Hanifi (2015) any of the groups												
Hibberd (2019)												

Huang (2022)	■				■							
Kang (2021)	■	■										
Kim (2021)	■		■	■						■		
Lee (2021)			■									
Li (2023)	■	▒			▒				▒			
López-Garcia (2023)	■	■			■		■					
Majeed (2023)	■	■			■							
Michael (2020)	■	■										
Moloney (2021)	■	■	■		■					■		
Moore (2023)	■		■		■							

Nakamura (2022)												
Pagliai (2023) [b]												
Park (2020)												
Paytuvi-Gallart (2020)												
Plaza-Diaz (2015) *L. rhamnosus* CNCM I-4036 c												
Plaza-Diaz (2015) *L. paracasei* CNCM I-4034 c												
Plaza-Diaz (2015) *B. breve* CNCM I-4035 c												
Plaza-Diaz (2015) *B. breve* CNCM I-4035 and *L. rhamnosus* CNCM I-4036 c												
Qian (2020)												

Rahayu (2021) [d]												
Sánchez Macarro (2021)												
Sandionigi (2022)												
Shi (2020)												
Shi (2023)												
Simon (2015)												
Sohn (2022)												
Son (2020)												
Tremblay (2021) 5B CFU												
Tremblay (2021) 25B CFU												

Wahburn (2022)												
Wischmeyer (2024)												

	no significant difference in α-diversity between groups
	favors probiotics (higher α-diversity in the intervention group compared to baseline and/or to placebo)
	favors placebo (higher α-diversity in the control group compared to baseline and/or to probiotics)
	not applicable*

Definitions for each outcome are detailed in Table S3.

[a] Gan et al. reported an increase in alpha diversity in the stools of the placebo group; however, did not specify which index was exactly affected.

[b] Instead of Observed OTUs, Observed Amplicon Sequence Variants were reported.

[c] No information about the placebo group.

[d] Rahayu et al. reported significant increases in the probiotic group; however, a comparison with the placebo group was not performed. According to our comparative meta-analysis the mean of median differences were not significantly different in the two groups after the intervention period.

*If a study did not investigate a specific outcome, “not applicable” is indicated.

Abbreviations: OTU: operational taxonomic unit; ACE: Abundance-based coverage estimator

Table S5 Changes in the microbiome β-diversity indices as measured after the intervention period.

Study	Bray-Curtis (dis)similarity index	Euclidean distance	Weighted UniFrac distance	Unweighted UniFrac distance	Generalized UniFrac distance	Jensen-Shannon divergence	Morisita-Horn distance metrics.	Spearman correlation distance	Not clearly specified
Bagga (2018)	■								
Bazanella (2017)					■				
Bloemendaal (2021)			■						
Boesmans (2018)	■								
Castanet (2020)									■
Chen (2021)	■								
Chen (2023)	■								
Ferrario (2014)			▥						

Freedman 2021									
Gai (2023)									
Gan (2022)									
Gargari (2016)									
Hanifi (2015) *any of the groups									
Hibberd (2019)									
Huang (2022)									
Kang (2021)									
Lau (2018)									
Lee (2021)									

Li (2023)									
Majeed (2023)									
Marcial (2017)									
Michael (2020)									
Moore (2023)									
Mutoh (2024)									
Nakamura (2022)									
Pagliai (2023)									
Park (2020)									
Paytuvi-Gallart (2020)									

Plaza Diaz (2015) L.rhamnosus group [a]									
Plaza-Diaz (2015) L. paracasei CNCM I-4034 [a]									
Plaza-Diaz (2015) B. breve CNCM I-4035 [a]									
Plaza-Diaz (2015) B. breve CNCM I-4035 and L. rhamnosus CNCM I-4036 [a]									
Qian (2020)									
Sandionigi(2022)									
Shi (2020)									
Shi (2023)									
Simon (2015)									

Sohn (2022)	there is a significant difference between the groups								
Tremblay (2021) 5B CFU									no significant difference in β-diversity between groups
Tremblay (2021) 25B CFU									
Washburn (2022)	no significant difference in β-diversity between groups								
Wischmeyer (2024)	there is a significant difference between the groups								

	no significant difference in β-diversity between groups
	there is a significant change in the probiotic group compared to baseline
	there is a significant change in the placebo group compared to baseline
	there is a significant difference between the groups
	not applicable*

Definitions for each outcome are detailed in Table S3.

[a] No information about placebo.

*If a study did not investigate a specific outcome, "not applicable" is indicated.

Abbreviations: UniFrac: unique fraction metric

Tables S6-7 Risk of bias assessments

Table S6 Risk of bias assessment for parallel design studies.

Risk of Bias assessment_Parallel		D1	D2	D3	D4	D5	Overall
Study	Aim	Randomization process	Deviations from the intended interventions	Missing outcome data	Measurement of the outcome	Selection of the reported result	
Bagga (2018)	assignment to intervention (the 'intention-to-treat' effect)	Low	Some concerns	High	Low	Low	High
Bazanella (2017)	adhering to intervention (the 'per-protocol' effect)	Low	Low	Low	Low	Low	Low
Bloemendaal (2021)	assignment to intervention (the 'intention-to-treat' effect)	Low	Some concerns	Low	Low	Some concerns	Some concerns
Castanet (2020)	adhering to intervention (the 'per-protocol' effect)	Low	Some concerns	Low	Low	Low	Some concerns
Chen (2021)	adhering to intervention (the 'per-protocol' effect)	Low	Low	Low	Low	Low	Low
Chen (2023)	assignment to intervention (the 'intention-to-treat' effect)	Low	Some concerns	Low	Low	Low	Some concerns
De Andrés (2018)	adhering to intervention (the 'per-protocol' effect)	Low	Low	Low	Low	Low	Low
Freedman (2021)	adhering to intervention (the 'per-protocol' effect)	Low	Low	Low	Low	Low	Low
Gai (2023)	adhering to intervention (the 'per-protocol' effect)	Low	High	Low	Low	Some concerns	High
Hibberd (2019)	adhering to intervention (the 'per-protocol' effect)	Low	Low	Low	Low	Low	Low
Huang (2022)	adhering to intervention (the 'per-protocol' effect)	Some concerns	High	Low	Low	Some concerns	High
Kang (2021)	assignment to intervention (the 'intention-to-treat' effect)	Low	Low	Low	Low	Low	Low

Lee (2021)	adhering to intervention (the 'per-protocol' effect)	Low	Low	Low	Low	Low	Some concerns
Li (2023)	adhering to intervention (the 'per-protocol' effect)	Low	Low	Low	Low	Low	Low
López-Garcia (2023)	assignment to intervention (the 'intention-to-treat' effect)	Low	Some concerns	Low	Low	Some concerns	Some concerns
Majeed (2023)	adhering to intervention (the 'per-protocol' effect)	Low	Low	Low	Low	Low	Low
Michael (2020)	adhering to intervention (the 'per-protocol' effect)	Low	High	Low	Low	Low	High
Moore (2023)	assignment to intervention (the 'intention-to-treat' effect)	Low	Some concerns	Low	Low	Low	Some concerns
Park (2020)	assignment to intervention (the 'intention-to-treat' effect)	Low	Some concerns	Low	Low	Low	Some concerns
Paytuví-Gallart (2020)	adhering to intervention (the 'per-protocol' effect)	Low	High	Low	Low	Low	High
Rahayu (2021)	assignment to intervention (the 'intention-to-treat' effect)	Low	Some concerns	Low	Low	Low	Some concerns
Sánchez Macarro (2021)	adhering to intervention (the 'per-protocol' effect)	Low	Low	Low	Low	Low	Low
Sandionigi (2022)	assignment to intervention (the 'intention-to-treat' effect)	Low	Low	Low	Low	Some concerns	Some concerns
Shi (2023)	assignment to intervention (the 'intention-to-treat' effect)	Low	Low	Low	Low	Low	Low
Shi (2020)	assignment to intervention (the 'intention-to-treat' effect)	High	Some concerns	Low	Low	Some concerns	High
Sohn (2022)	assignment to intervention (the 'intention-to-treat' effect)	Low	Some concerns	Low	Low	Low	Some concerns
Son (2020)	adhering to intervention (the 'per-protocol' effect)	Low	High	Low	Low	Some concerns	High
Tremblay (2021)	assignment to intervention (the 'intention-to-treat' effect)	Low	Some concerns	Low	Low	Low	Some concerns
Washburn (2022)	adhering to intervention (the 'per-protocol' effect)	Low	Low	Low	Low	Low	Low

Table S7 Risk of bias assessment for cross-over design studies

**Risk of Bias assessment_Cross-over**		**D1**	**DS**	**D2**	**D3**	**D4**	**D5**	**Overall**
**Study**	**Aim**	Randomization process	Bias arising from period and carryover effects	Deviations from the intended interventions	Missing outcome data	Measurement of the outcome	Selection of the reported result	
Axelrod (2019)	assignment to intervention (the 'intention-to-treat' effect)	Low	Some concerns	Some concerns	Low	Low	Some concerns	High
Moloney (2021)	assignment to intervention (the 'intention-to-treat' effect)	Low	Some concerns	High	Low	Low	Some concerns	High

Figs. S21-24 Risk of bias assessments

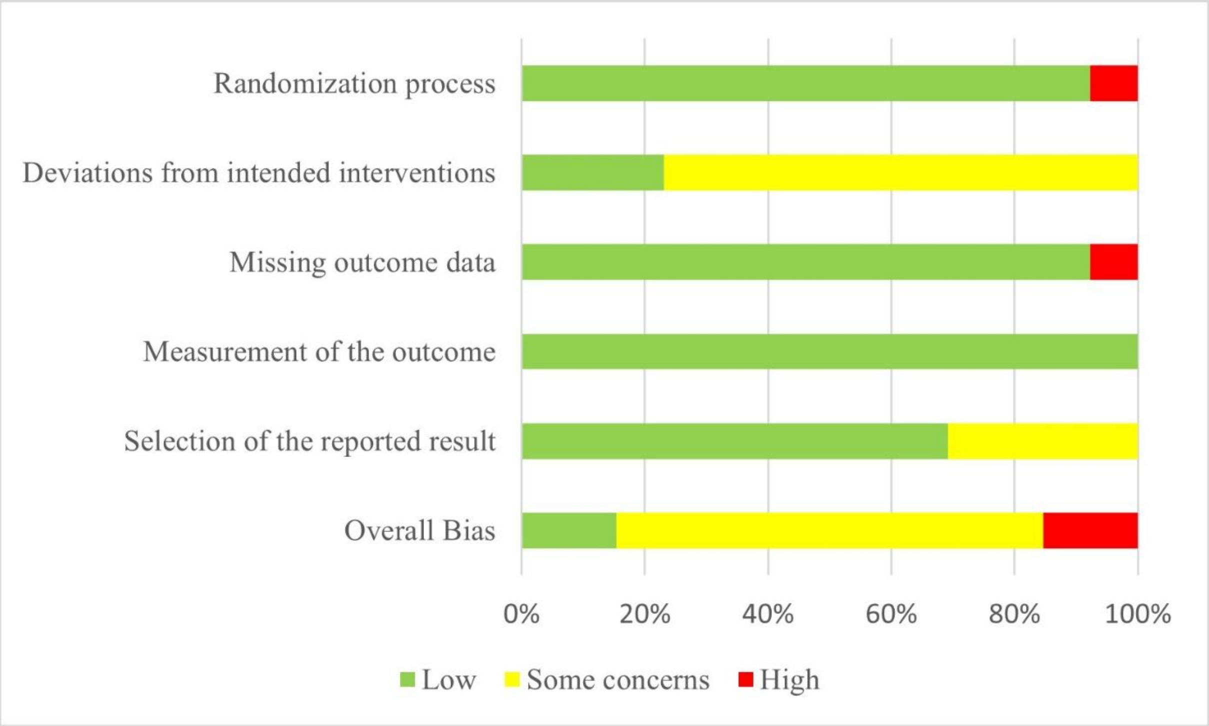

Fig. S21 Risk of bias assessment for parallel design studies - Assignment to intervention (the 'intention-to-treat' effect).

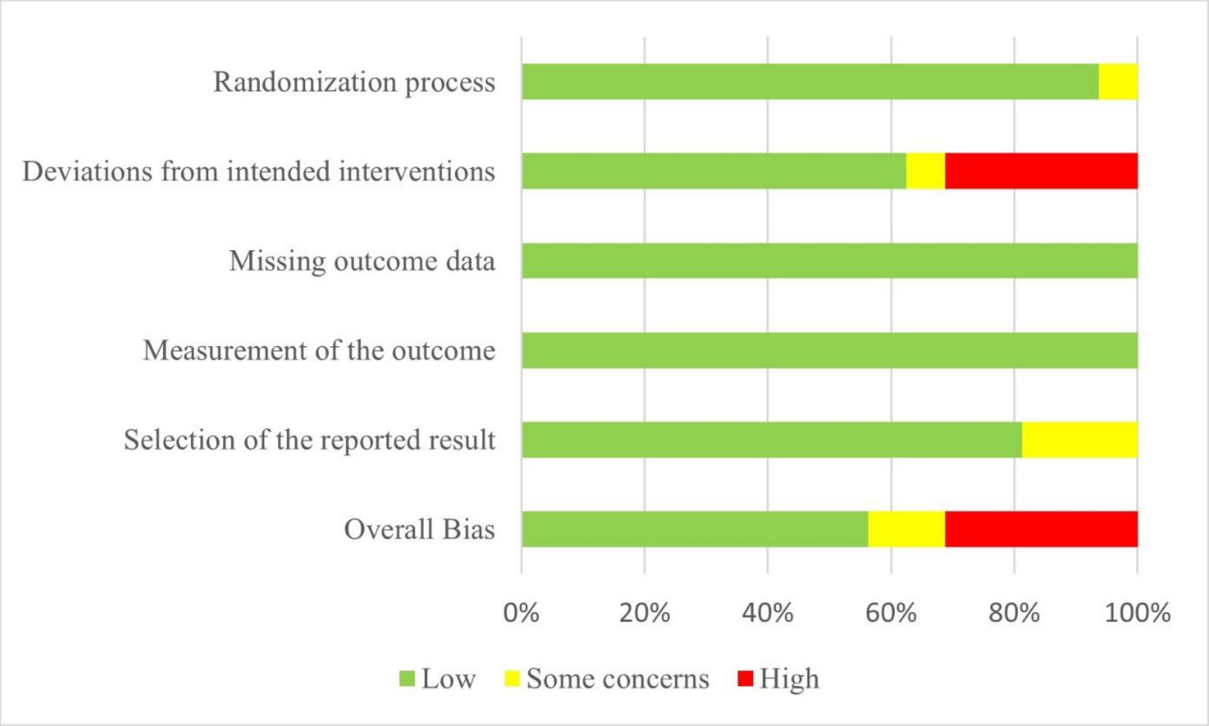

Fig. S22 Risk of bias assessment for parallel design studies - Adhering to intervention (the 'per-protocol' effect).

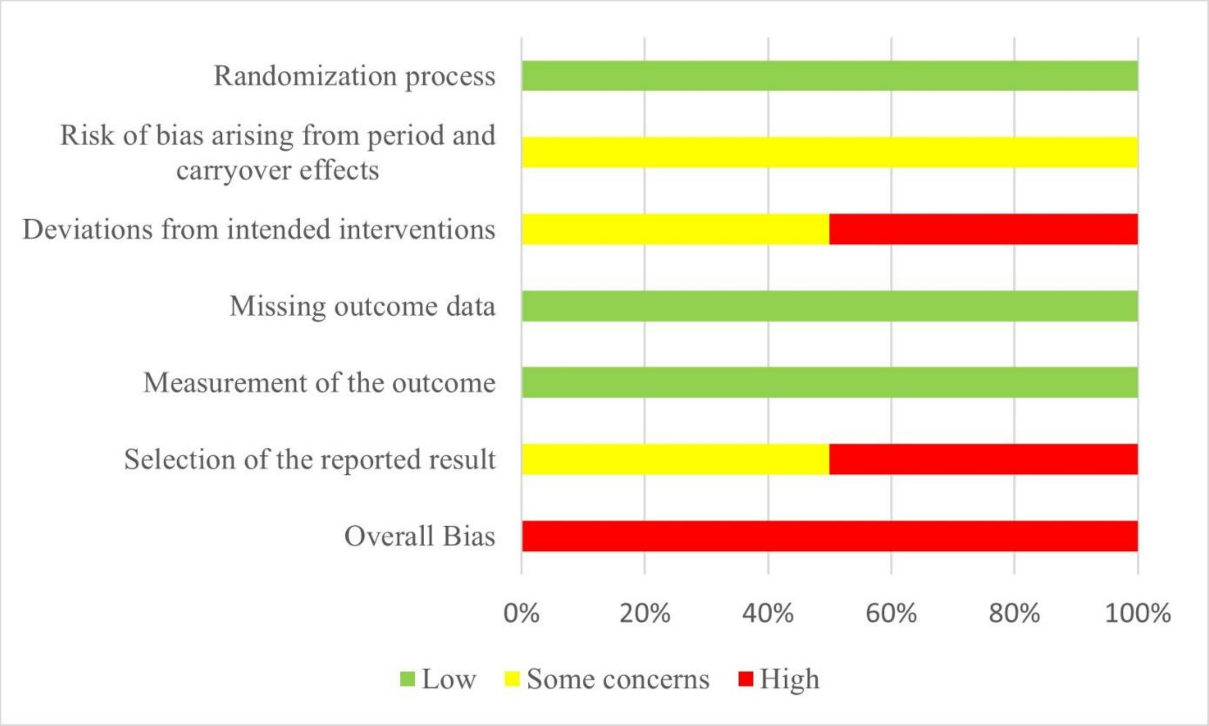

Fig. S23 Risk of bias assessment for cross-over design studies - Assignment to intervention (the 'intention-to-treat' effect).

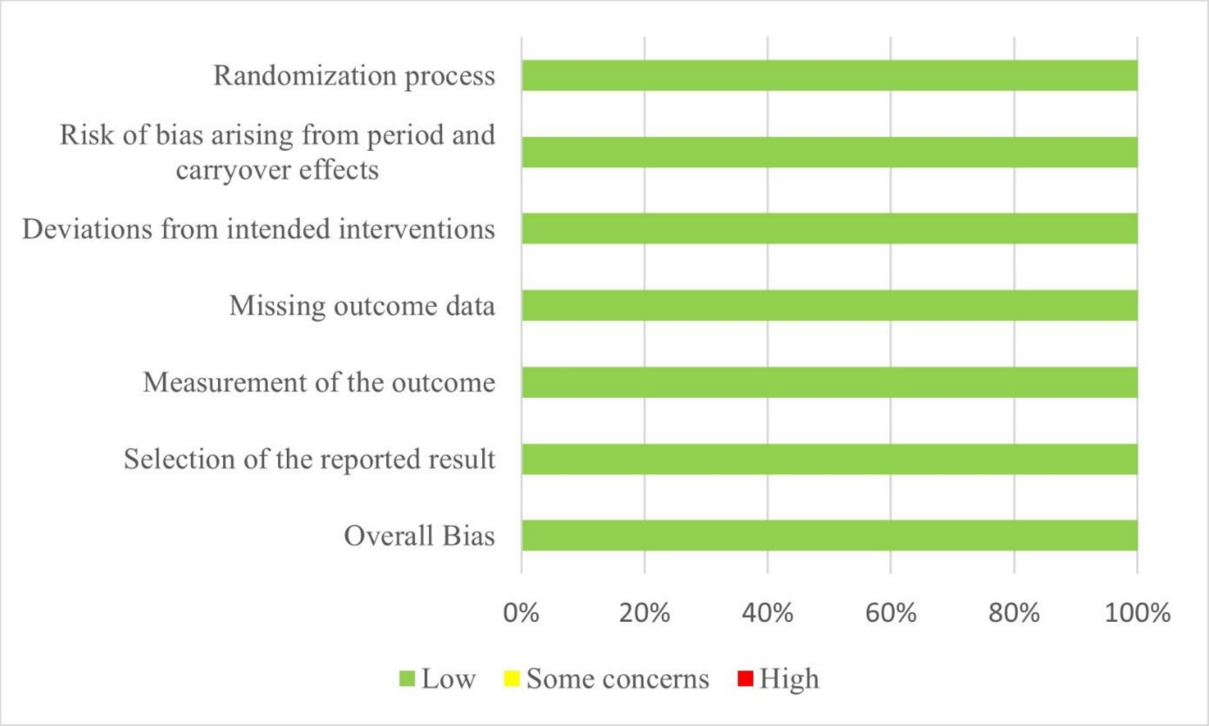

Fig. S24 Risk of bias assessment for crossover design studies - Adhering to intervention (the 'per-protocol' effect).

Table S8 GRADE assessment for the meta-analyses of Shannon, Observed OTUs, Chao1 and Simpson's Index of Diversity indices.

Certainty assessment							№ of patients		Effect		Certainty	Importance
№ of studies	Study design	Risk of bias	Inconsistency	Indirectness	Imprecision	Other considerations	probiotics	control	Relative (95% CI)	Absolute (95% CI)		
**Shannon diversity index (follow-up: range 2 weeks to 6 months; assessed with: 16S rRNA sequencing)**												
22	randomised trials	serious[a]	not serious	not serious	serious[b]	none	552	552	-	MedD **0.08 lower** (0.16 lower to 0.01 higher)	⊕⊕◯◯ Low[a,b]	IMPORTANT
**Observed OTUs (follow-up: range 4 weeks to 22 weeks; assessed with: 16S rRNA sequencing)**												
7	randomised trials	serious[a]	not serious	not serious	serious[c]	none	231	232	-	MedD **2.19 higher** (2.2 lower to 6.57 higher)	⊕⊕◯◯ Low[a,c]	IMPORTANT
**Chao1 index (follow-up: range 4 weeks to 24 weeks; assessed with: 16S rRNA sequencing)**												

Certainty assessment							№ of patients		Effect		Certainty	Importance
№ of studies	Study design	Risk of bias	Inconsistency	Indirectness	Imprecision	Other considerations	probiotics	control	Relative (95% CI)	Absolute (95% CI)		
9	randomised trials	serious[a]	not serious	not serious	serious[d]	none	252	252	-	MedD **3.19 lower** (27.28 lower to 20.89 higher)	⊕⊕◯◯ Low[a,d]	IMPORTANT
**Simpson's Index of Diversity (follow-up: range 4 weeks to 22 weeks; assessed with: 16S rRNA sequencing)**												
10	randomised trials	serious[a]	not serious	not serious	not serious	none	236	235	-	MedD **0.01 lower** (0.02 lower to 0)	⊕⊕⊕◯ Moderate[a]	IMPORTANT

CI: confidence interval **MedD**: mean of median differences

Explanations

a. Although subgroup analysis did not demonstrate a difference in effect by risk of bias level, a substantial proportion of studies contributing to the evidence were classified as high or some concerns risk of bias, which may still downgrade the confidence in the estimate due to potential unmeasured sources of bias.

b. The total sample size exceeds standard optimal information size benchmarks for continuous outcomes, so sample size alone is sufficient. However, the pooled 95% confidence interval for the effect estimate is [-0.16; 0.01], which crosses the null value and includes both possible benefit and no effect. The prediction interval further demonstrates uncertainty, as it encompasses clinically relevant directions. As a result, the certainty of evidence is downgraded by one level for imprecision.

c. The total sample size for the outcome is limited, and the 95% confidence interval for the pooled effect estimate (-2.20 to 6.57) is wide, including both the possibility of a clinically important increase or decrease as well as no effect. Despite consistent effects across studies (I^2 = 0%), the insufficient information size and broad confidence interval warrant downgrading the certainty of evidence by one level for imprecision.

d. The total sample size for the Chao1 index meta-analysis is limited, and the pooled confidence interval (-27.28 to 20.89) is wide, encompassing both possible harm, benefit, and no effect. The prediction interval is similarly broad, suggesting substantial uncertainty about the true effect. As a result, the certainty of evidence is downgraded for imprecision.

References to Supplementary material

1. Taur Y, Jenq RR, Perales MA, Littmann ER, Morjaria S, Ling L, No D, Gobourne A, Viale A, Dahi PB, et al. The effects of intestinal tract bacterial diversity on mortality following allogeneic hematopoietic stem cell transplantation. Blood 2014;124:1174–82.
2. Rain R, Czernia D, Bowater J. Shannon diversity index calculator [Internet]. Omni Calculator. 2022. Available from: https://www.omnicalculator.com/ecology/shannon-index
3. Lankelma JM, van Vught LA, Belzer C, Schultz MJ, van der Poll T, de Vos WM, Wiersinga WJ. Critically ill patients demonstrate large interpersonal variation in intestinal microbiota dysregulation: a pilot study. Intensive Care Med Springer Berlin Heidelberg; 2017;43:59–68.
4. Éliás AJ, Barna V, Patoni C, Demeter D, Veres DS, Bunduc S, Erőss B, Hegyi P, Földvári-Nagy L, Lenti K. Probiotic supplementation during antibiotic treatment is unjustified in maintaining the gut microbiome diversity: a systematic review and meta-analysis. BMC Med BioMed Central Ltd; 2023;21.
5. Blaxter M, Mann J, Chapman T, Thomas F, Whitton C, Floyd R, Abebe E. Defining operational taxonomic units using DNA barcode data. Philosophical Transactions of the Royal Society B: Biological Sciences 2005;360:1935–43.
6. Chao A. Nonparametric Estimation of the Number of Classes in a Population. Scandinavian Journal of Statistics 1984;11:265–70.
7. Faith DP. Conservation evaluation and phylogenetic diversity. Biol Conserv 1992;61:1–10.
8. Strong WL. Assessing species abundance unevenness within and between plant communities. Community Ecology 2002;3:237–46.
9. Pielou EC. The measurement of diversity in different types of biological collections. J Theor Biol 1966;13:131–44.
10. Gotelli NJ, Colwell RK. Estimating species richness. Biological Diversity. Frontiers in Measurement and Assessment, Oxford University Press, United Kingdom; 2011. p. 39–54.
11. Chao A, Lee SM. Estimating the number of classes via sample coverage. J Am Stat Assoc 1992;87:210–7.
12. Chao A, Yang MCK. Stopping rules and estimation for recapture debugging with unequal failure rates. Biometrika 1993;80:193–201.
13. Simpson EH. Measurement of Diversity. Nature 1949;163:688.
14. Jost L. What do we mean by diversity?: The path towards quantification. Metode Universitat de Valencia; 2019;2019:55–61.
15. Kitikidou K, Milios E, Stampoulidis A, Pipinis E, Radoglou K. Using Biodiversity Indices Effectively: Considerations for Forest Management. Ecologies Multidisciplinary Digital Publishing Institute (MDPI); 2024;5:42–51.
16. Jost L. Entropy and diversity. Oikos. 2006. p. 363–75.

17. Hill M 0. DIVERSITY AND EVENNESS: A UNIFYING NOTATION AND ITS CONSEQUENCES 1.

18. Bray JR, Curtis JT, Roger J. This content downloaded from 147.8.31.43 on Mon. Source: Ecological Monographs 1957;27:325–49.

19. Zeleny D. Ecological Resemblance Notes [Internet]. 1966. p. 1–10. Available from: en:similarity https://www.davidzeleny.net/anadat-r/doku.php/en:similarity

20. Lozupone CA, Hamady M, Kelley ST, Knight R. Quantitative and qualitative β diversity measures lead to different insights into factors that structure microbial communities. Appl Environ Microbiol 2007;73:1576–85.

21. Chen J, Bittinger K, Charlson ES, Hoffmann C, Lewis J, Wu GD, Collman RG, Bushman FD, Li H. Associating microbiome composition with environmental covariates using generalized UniFrac distances. Bioinformatics 2012;28:2106–13.

22. Nielsen F. On the Jensen-Shannon symmetrization of distances relying on abstract means. Entropy MDPI AG; 2019;21.

23. Morisita M. Measuring of interspecific association and similarity between communities. Memoirs of the Faculty of Science, Kyushu University 1959;3:65–80.

24. Andermann T, Antonelli A, Barrett RL, Silvestro D. Estimating Alpha, Beta, and Gamma Diversity Through Deep Learning. Front Plant Sci 2022;13.

25. Lozupone CA, Stombaugh JI, Gordon JI, Jansson JK, Knight R. Diversity, stability and resilience of the human gut microbiota. Nature 2012;489:220–30.

26. Roswell M, Dushoff J, Winfree R. A conceptual guide to measuring species diversity. Oikos 2021;130:321–38.

27. Xu S, Böttcher L, Chou T. Diversity in biology: Definitions, quantification and models. Phys Biol 2020;17.

28. McGrath S, Zhao XF, Ozturk O, Katzenschlager S, Steele R, Benedetti A. metamedian: An R package for meta-analyzing studies reporting medians. Res Synth Methods John Wiley and Sons Ltd; 2024;15:332–46.

29. López-García E, Benítez-Cabello A, Arenas-de Larriva AP, Gutierrez-Mariscal FM, Pérez-Martínez P, Yubero-Serrano EM, Garrido-Fernández A, Arroyo-López FN. Oral intake of Lactiplantibacillus pentosus LPG1 Produces a Beneficial Regulation of Gut Microbiota in Healthy Persons: A Randomised, Placebo-Controlled, Single-Blind Trial. Nutrients MDPI; 2023;15.

30. Veroniki AA, Jackson D, Viechtbauer W, Bender R, Bowden J, Knapp G, Kuss O, Higgins JP, Langan D, Salanti G. Methods to estimate the between-study variance and its uncertainty in meta-analysis. Res Synth Methods 2016;7:55–79.

31. Inthout J, Ioannidis JP, Borm GF. The Hartung-Knapp-Sidik-Jonkman method for random effects meta-analysis is straightforward and considerably outperforms the standard DerSimonian-Laird method. BMC Med Res Methodol 2014;14:1–12.

32. Knapp G, Hartung J. Improved tests for a random effects meta-regression with a single covariate. Stat Med 2003;22:2693–710.

33. Jackson D, Law M, Rücker G, Schwarzer G. The Hartung-Knapp modification for random-effects meta-analysis: A useful refinement but are there any residual concerns? Stat Med John Wiley and Sons Ltd; 2017;36:3923–34.

34. Harrer M, Cuijpers P, Furukawa TA, Ebert DD. Doing Meta-Analysis with R: A Hands-On Guide. 1st ed. Boca Raton, London: Chapman and Hall/CRC Press; 2021.

35. Moloney GM, Long-Smith CM, Murphy A, Dorland D, Hojabri SF, Ramirez LO, Marin DC, Bastiaanssen TFS, Cusack AM, Berding K, et al. Improvements in sleep indices during exam stress due to consumption of a Bifidobacterium longum. Brain Behav Immun Health Elsevier Inc.; 2021;10.

36. Nakamura Y, Suzuki S, Murakami S, Nishimoto Y, Higashi K, Watarai N, Umetsu J, Ishii C, Ito Y, Mori Y, et al. Integrated gut microbiome and metabolome analyses identified fecal biomarkers for bowel movement regulation by Bifidobacterium longum BB536 supplementation: A RCT. Comput Struct Biotechnol J Elsevier B.V.; 2022;20:5847–58.

37. Bloemendaal M, Szopinska-Tokov J, Belzer C, Boverhoff D, Papalini S, Michels F, van Hemert S, Arias Vasquez A, Aarts E. Probiotics-induced changes in gut microbial composition and its effects on cognitive performance after stress: exploratory analyses. Transl Psychiatry Springer Nature; 2021;11.

38. Moore RL, Feehily C, Killeen SL, Yelverton CA, Geraghty AA, Walsh CJ, O'Neill IJ, Nielsan IB, Lawton EM, Sanchez-Gallardo R, et al. Ability of Bifidobacterium breve 702258 to transfer from mother to infant: the MicrobeMom randomized controlled trial. Am J Obstet Gynecol MFM Elsevier Inc.; 2023;5.

39. Gargari G, Taverniti V, Balzaretti S, Ferrario C, Gardana C, Simonetti P, Guglielmetti S. Consumption of a bifidobacterium bifidum strain for 4 weeks modulates dominant intestinal bacterial taxa and fecal butyrate in healthy adults. Appl Environ Microbiol American Society for Microbiology; 2016;82:5850–9.

40. Kang S, Park MY, Brooks I, Lee J, Kim SH, Kim JY, Oh B, Kim JW, Kwon O. Spore-forming Bacillus coagulans SNZ 1969 improved intestinal motility and constipation perception mediated by microbial alterations in healthy adults with mild intermittent constipation: A randomized controlled trial. Food Research International Elsevier Ltd; 2021;146.

41. Shi S, Zhang Q, Sang Y, Ge S, Wang Q, Wang R, He J. Probiotic Bifidobacterium longum BB68S Improves Cognitive Functions in Healthy Older Adults: A Randomized, Double-Blind, Placebo-Controlled Trial. Nutrients MDPI; 2023;15.

42. Gai Z, Dong Y, Xu F, Zhang J, Yang Y, Wang Y. Changes in the gut microbiota composition of healthy young volunteers after administration of Lacticaseibacillus rhamnosus LRa05: A placebo-controlled study. Front Nutr Frontiers Media S.A.; 2023;10.

43. Shi J, Gao F, Zhang J. Effect of Combined Live Probiotics Alleviating the Gastrointestinal Symptoms of Functional Bowel Disorders. Gastroenterol Res Pract Hindawi Limited; 2020;2020.
